# Antivirals for post-exposure prophylaxis of influenza: a systematic review and network meta-analysis

**DOI:** 10.1016/S0140-6736(24)01357-6

**Published:** 2024-08-24

**Authors:** Yunli Zhao, Ya Gao, Gordon Guyatt, Timothy M Uyeki, Ping Liu, Ming Liu, Yanjiao Shen, Xiaoyan Chen, Shuyue Luo, Xingsheng Li, Rongzhong Huang, Qiukui Hao

**Affiliations:** a Department of Gerontology, The Second Affiliated Hospital of Chongqing Medical University, Chongqing, China; b Chongqing Municipality Clinical Research Center for Geriatrics, The Second Affiliated Hospital of Chongqing Medical University, Chongqing, China; c Precision Medicine Center, The Second Affiliated Hospital of Chongqing Medical University, Chongqing, China; d Evidence-Based Medicine Center, School of Basic Medical Sciences, Lanzhou University, Lanzhou, China; e Department of Health Research Methods, Evidence, and Impact, McMaster University, Hamilton, ON, Canada; f School of Rehabilitation Science, McMaster University, Hamilton, ON, Canada; g Influenza Division, US Centers for Disease Control and Prevention, Atlanta, GA, USA; h Department of Neurology, The First Affiliated Hospital of Chongqing Medical University, Chongqing, China; i Innovation Institute for Integration of Medicine and Engineering, Chinese Evidence-Based Medicine Center, West China Hospital, Sichuan University, Chengdu, China; j The Center of Gerontology and Geriatrics (National Clinical Research Center for Geriatrics), West China Hospital, Sichuan University, Chengdu, China

## Abstract

**Background:**

Antiviral post-exposure prophylaxis with neuraminidase inhibitors can reduce the incidence of influenza and the risk of symptomatic influenza, but the efficacy of the other classes of antiviral remains unclear. To support an update of WHO influenza guidelines, this systematic review and network meta-analysis evaluated antiviral drugs for post-exposure prophylaxis of influenza.

**Methods:**

We systematically searched MEDLINE, Embase, Cochrane Central Register of Controlled Trials, Cumulative Index to Nursing and Allied Health Literature, Global Health, Epistemonikos, and ClinicalTrials.gov for randomised controlled trials published up to Sept 20, 2023 that evaluated the efficacy and safety of antivirals compared with another antiviral or placebo or standard care for prevention of influenza. Pairs of reviewers independently screened studies, extracted data, and assessed the risk of bias. We performed network meta-analyses with frequentist random effects model and assessed the certainty of evidence using the GRADE (Grading of Recommendations Assessment, Development and Evaluation) approach. The outcomes of interest were symptomatic or asymptomatic infection, admission to hospital, all-cause mortality, adverse events related to antivirals, and serious adverse events. This study is registered with PROSPERO, CRD42023466450.

**Findings:**

Of 11 845 records identified by our search, 33 trials of six antivirals (zanamivir, oseltamivir, laninamivir, baloxavir, amantadine, and rimantadine) that enrolled 19 096 individuals (mean age 6·75–81·15 years) were included in this systematic review and network meta-analysis. Most of the studies were rated as having a low risk of bias. Zanamivir, oseltamivir, laninamivir, and baloxavir probably achieve important reductions in symptomatic influenza in individuals at high risk of severe disease (zanamivir: risk ratio 0·35, 95% CI 0·25–0·50; oseltamivir: 0·40, 0·26–0·62; laninamivir: 0·43, 0·30–0·63; baloxavir: 0·43, 0·23–0·79; moderate certainty) when given promptly (eg, within 48 h) after exposure to seasonal influenza. These antivirals probably do not achieve important reductions in symptomatic influenza in individuals at low risk of severe disease when given promptly after exposure to seasonal influenza (moderate certainty). Zanamivir, oseltamivir, laninamivir, and baloxavir might achieve important reductions in symptomatic zoonotic influenza in individuals exposed to novel influenza A viruses associated with severe disease in infected humans when given promptly after exposure (low certainty). Oseltamivir, laninamivir, baloxavir, and amantadine probably decrease the risk of all influenza (symptomatic and asymptomatic infection; moderate certainty). Zanamivir, oseltamivir, laninamivir, and baloxavir probably have little or no effect on prevention of asymptomatic influenza virus infection or all-cause mortality (high or moderate certainty). Oseltamivir probably has little or no effect on admission to hospital (moderate certainty). All six antivirals do not significantly increase the incidence of drug-related adverse events or serious adverse events, although the certainty of evidence varies.

**Interpretation:**

Post-exposure prophylaxis with zanamivir, oseltamivir, laninamivir, or baloxavir probably decreases the risk of symptomatic seasonal influenza in individuals at high risk for severe disease after exposure to seasonal influenza viruses. Post-exposure prophylaxis with zanamivir, oseltamivir, laninamivir, or baloxavir might reduce the risk of symptomatic zoonotic influenza after exposure to novel influenza A viruses associated with severe disease in infected humans.

**Funding:**

World Health Organization.

## Introduction

Influenza is an acute respiratory viral illness characterised often by sudden onset of dry cough, nasal congestion, and sore throat, with or without fever and myalgia, headache, and weakness.^1^ These symptoms might be accompanied by pulmonary and extrapulmonary complications, such as neurological, cardiac and renal dysfunction.^2^, ^3^ Influenza viruses cause annual seasonal epidemics and rare, unpredictable pandemics.^4^ All age groups can be affected by influenza, but young children (aged <2 years), pregnant women, older adults (aged ≥65 years), and individuals with chronic diseases are at a higher risk of severe disease. Although most people recover from influenza without medical attention, WHO estimates that 3–5 million cases of severe illness and up to 650 000 influenza-related deaths occur annually worldwide.^4^

**Research in context**

**Evidence before this study**

Antivirals can be used to prevent influenza in people who have been in close contact with people or animals infected with influenza viruses. Although previous systematic reviews have found that antivirals (oseltamivir or zanamivir) are effective in preventing symptomatic influenza, these reviews assessed selected antivirals and did not rate the quality of evidence or consider the importance of effects in their interpretation. Additionally, a randomised controlled trial of baloxavir for influenza post-exposure prophylaxis was not included in previous reviews.

**Added value of this study**

This systematic review and network meta-analysis of randomised controlled trials evaluating antiviral post-exposure prophylaxis for influenza was performed to support a WHO guideline development panel in formulating recommendations on the use of antivirals for influenza. We present analyses of the efficacy of antiviral post-exposure prophylaxis to prevent symptomatic influenza for high-risk or low-risk populations and to prevent symptomatic zoonotic influenza. We found evidence of moderate certainty that zanamivir, oseltamivir, laninamivir, and baloxavir probably reduce the risk of symptomatic seasonal influenza in individuals at high risk when administered promptly after exposure (eg, within 48 h) after exposure, but probably have little or no important effect in low-risk populations. Rimantadine probably has little or no effect on symptomatic seasonal influenza A virus infection (moderate certainty). Zanamivir, oseltamivir, laninamivir, and baloxavir might decrease the risk of symptomatic zoonotic influenza (low certainty). We found little evidence that amantadine prevents influenza A virus infection. We found no important impact on adverse events with any of these antivirals.

**Implications of all the available evidence**

The findings of this systematic review and network meta-analysis support use of zanamivir, oseltamivir, laninamivir, or baloxavir for post-exposure prophylaxis of seasonal influenza in individuals at high risk of severe influenza, and also provide some support for the use of these antivirals for post-exposure prophylaxis of zoonotic influenza. The findings do not support using these antivirals among low-risk populations for post-exposure prophylaxis of seasonal influenza and do not support the use of amantadine or rimantadine for preventing symptomatic influenza A virus infection.

Annual vaccination is recommended to prevent influenza, especially for those at a higher risk of severe disease.^1^ However, influenza vaccine effectiveness can vary by virus strain, population and year, and ranged from 10% to 60% in the USA between 2004 and 2022.^5^ When an influenza vaccine is unavailable or ineffective due to antigenic drift, viral immune evasion, or waning immunity, pre-exposure and post-exposure antiviral prophylaxis can be important for influenza prevention, particularly in individuals at increased risk of severe influenza complications.^6^, ^7^, ^8^

Previous systematic reviews have shown that antiviral post-exposure prophylaxis with neuraminidase inhibitors can reduce the incidence of influenza^9^ and the risk of symptomatic influenza in individuals and households.^10^, ^11^ However, none of these reviews addressed all available antivirals from the different classes of drugs with different mechanisms of action, and no reviews assessed the quality of the supportive evidence. Moreover, previous systematic reviews did not include all randomised controlled trials of antivirals for prevention of influenza, including one large trial that applied a novel antiviral, baloxavir, with a unique mechanism of action.^12^ The role of baloxavir in prophylaxis, especially compared with other antivirals such as oseltamivir and zanamivir, is not well understood. To address these evidence gaps and support an update of the WHO influenza guidelines,^13^ we conducted a systematic review and network meta-analysis to assess the efficacy and safety of all available approved antivirals for post-exposure prophylaxis of influenza.

## Methods

### Search strategy and selection criteria

To identify all randomised controlled trials of antiviral prophylaxis compared with placebo, standard care, or another antiviral for prevention of influenza, we conducted a comprehensive search of Embase, MEDLINE, Cochrane Central Registry of Controlled Trials, Cumulative Index to Nursing and Allied Health Literature, Global Health, Epistemonikos, and ClincalTrials.gov from database inception up to Sept 20, 2023. We collaborated with an experienced medical librarian to refine our search strategy for each database. No restrictions on language were applied. Details of the search strategy are presented in the appendix (pp 7–14). Additionally, we searched the reference lists of included studies and relevant systematic reviews to identify additional potentially eligible studies.

We included randomised controlled trials of direct-acting antivirals for prevention of influenza, including but not limited to neuraminidase inhibitors, viral polymerase complex inhibitors, cap-dependent endonuclease inhibitors, and matrix protein 2 ion channel inhibitors, compared with placebo or standard care alone or to another antiviral in individuals exposed to influenza viruses. Eligible randomised controlled trials diagnosed influenza virus infection in respiratory specimens by RT-PCR, rapid antigen test, or immunofluorescence assay.

Reviewers (YZ, YG, PL, ML, YS, XC, and SL), working in pairs, independently performed study selection, including screening titles and abstracts, and evaluated full-text eligibility of potentially eligible studies using standardised forms. Reviewers resolved disagreements by discussion or, when necessary, by consultation with a third reviewer.

This protocol for systematic review and network meta-analysis adheres to the PRISMA-P statement and is registered with PROSPERO (CRD42023466450). The coauthors of this study worked with the independent WHO guidelines panel to identify potential patient-important outcomes, pre-set subgroup analyses, and establish minimal important difference (MID) values.

### Data analysis

For each eligible study, two reviewers (YZ, YG, PL, ML, YS, XC, and SL), working in pairs, independently extracted the following data: study characteristics, patient characteristics, antiviral characteristics, specific influenza testing used to confirm influenza, follow-up time, and all potentially important patient outcomes. Patient outcomes assessed in this study included data on events or time to symptom onset, asymptomatic or symptomatic infection, duration of symptoms, admission to hospital, length of hospitalisation, progression to invasive mechanical ventilation, admission to an intensive care unit (ICU), length of mechanical ventilation, progression of disease severity, emergence of antiviral resistance, all-cause mortality, adverse events related to antivirals, and serious adverse events.

For included trials that used individual randomisation, reviewers independently assessed the risk of bias using the modified Cochrane risk of bias instrument (appendix p 15).^14^, ^15^ For trials that used cluster randomisation, reviewers independently assessed the risk of bias using the Cochrane risk of bias instrument 2 (appendix p 15).^16^

To compare the efficacy and safety of different antivirals for post-exposure prophylaxis of influenza, we performed network meta-analyses using the netmeta package of R version 4.0.2.^17^ We used frequentist random-effects network meta-analyses to estimate the relative effect of all interventions, a design-by-treatment interaction model (global test) to assess the coherence assumption for the entire network, and node-splitting methods to assess the local incoherence between direct and indirect estimates in every closed loop of evidence.^18^ The frequentist random-effects model accounts for both within-study and between-study variance. This approach generally provides more conservative estimates of the relative effects of various antivirals in the network meta-analysis than the Bayesian model when synthesising data from studies with certain heterogeneity.

We performed pairwise meta-analysis using the meta and metafor packages of R version 4.0.2. We applied the Hartung–Knapp–Sidik–Jonkman random-effects model to synthesise the data for pairwise meta-analyses when fewer than 20 studies included; otherwise, we used the DerSimonian and Laird random-effects model.^19^

For binary outcomes, we calculated risk ratios (RRs) and the associated 95% CIs and assessed the risk differences by applying the median in the control group from eligible randomised controlled trials as the baseline mean.^20^ When the control event rate was less than 1%, we calculated the pooled absolute risk difference and its CI directly. For the cluster-randomised trials that failed to conduct appropriate analyses and did not report their effective sample sizes, we used the intracluster correlation coefficient (ICC=0·02 in main analyses and ICC=0·10 as one of the sensitivity analyses) and the number of clusters to recalculate the design effects and the number of events.^16^ We reported the adjusted sample sizes and numbers of events in the meta-analyses. For any zero number cell, we added 0·5 to the cell with no event.^21^

We assessed heterogeneity between studies with the use of restricted maximum likelihood models to calculate τ^2^ and *I*^2^,^22^ and by visually inspecting the forest plots for differences in magnitude. We conducted within-trial comparisons when data were available and, if not available, between-trial comparisons if there were at least two studies in each subgroup. If there was a potential subgroup effect (p<0·10), we used the Instrument for the Credibility of Effect Modification Analyses (ICEMAN) tool to assess the credibility of the subgroup effect.^23^ Our planned subgroup analyses included influenza virus type, age, exposure status, source of infection, influenza vaccination status, and disease severity. Our prior hypotheses and the anticipated direction of effects are described in the appendix (p 16).

When there were ten or more eligible studies, we used funnel plots, Egger's test (for continuous variables), and Harbord's test (for discontinuous variables)^24^ to assess publication bias.

We used the Grading of Recommendations Assessment, Development and Evaluation (GRADE) approach to assess the certainty of evidence for network meta-analyses.^25^, ^26^ We rated the overall certainty of evidence in absolute effects. When using RR as the relative effect measure, we calculated absolute effects using the relative effect estimates and the baseline risk estimates. We used the median rate in the placebo or standard care group of participants in eligible randomised controlled trials as the baseline risk. For the development of symptomatic zoonotic influenza after exposure to animals or people infected with novel influenza A viruses associated with severe disease and high mortality in infected humans (eg, avian influenza A[H5N1], A[H5N6], and A[H7N9] viruses), the WHO guidelines panel estimated the baseline risk of symptomatic zoonotic influenza to be 3%, with 80% of patients with symptomatic zoonotic influenza requiring hospital admission, and 30% mortality. The methods for rating the certainty of evidence are described in the appendix (p 17).

To assess imprecision for each outcome, we collaborated with the WHO guideline panel to establish MID values as the thresholds for assessing important patient outcomes. The panel established MID thresholds of 0·3% for all-cause mortality, 1·5% for hospitalisation, 1% for drug-related adverse events, and 0·5% for serious drug-related adverse events. For antiviral prophylaxis of laboratory-confirmed symptomatic seasonal influenza, the MID threshold was 5·5% for low-risk populations and 3% for high-risk populations. The panel defined the high-risk population based on the 2022 WHO influenza guidelines (appendix p 18);^13^ it included individuals with chronic medical conditions (eg, immunosuppression or obesity) and certain age groups such as young children (age <5 years) and older adults (age ≥65 years). Low-risk populations included healthy individuals without chronic medical conditions or major risk factors for severe influenza.

### Role of the funding source

The funder of the study had no role in study design, data collection, data analysis, data interpretation, or writing of the report.

## Results

**Figure 1:**
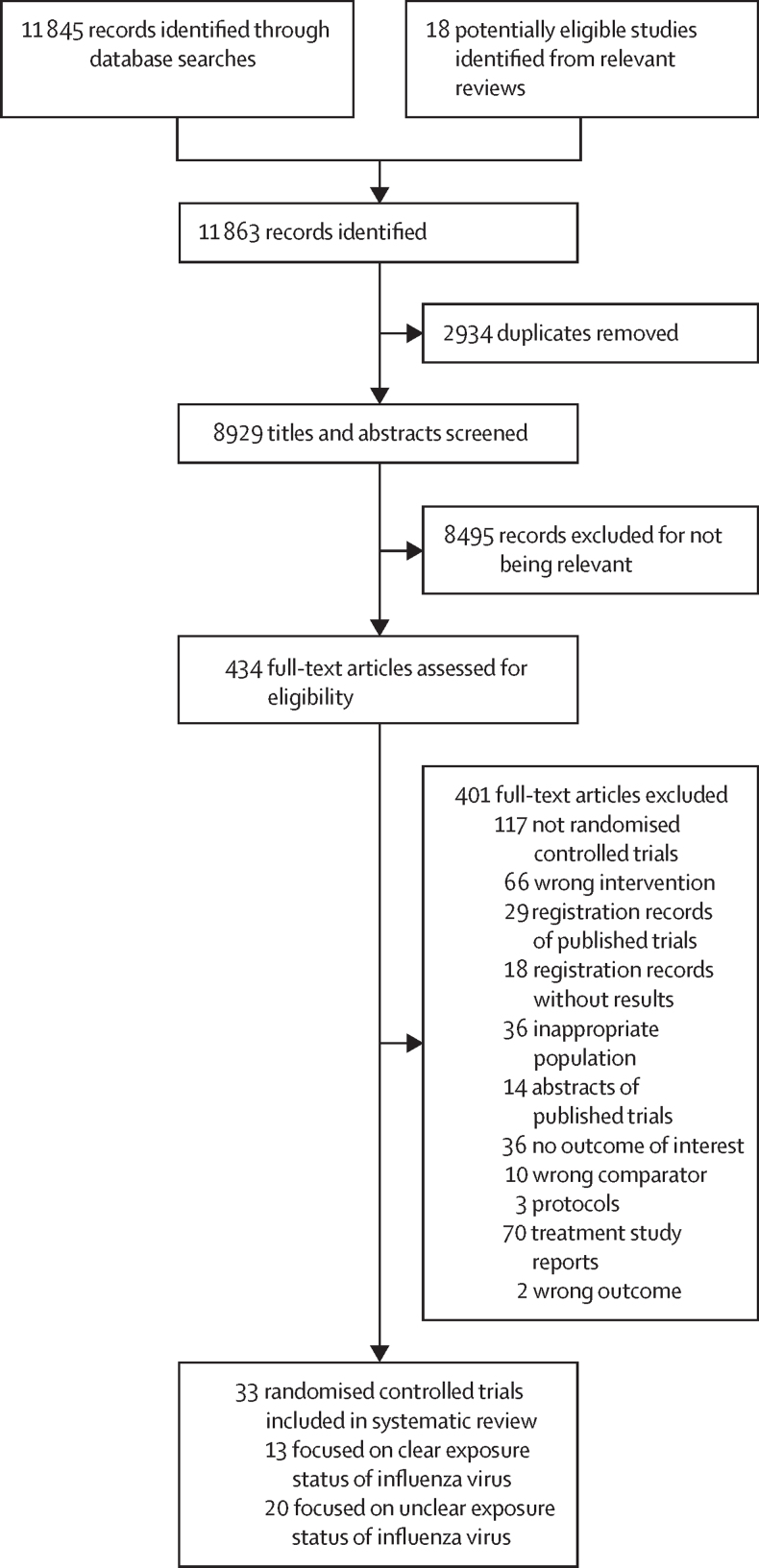
Study selection

We identified 11 845 publications through database searches and 18 publications from relevant reviews, of which 434 studies were potentially eligible during the screening of titles and abstracts, and 33 studies were eligible on full-text review for inclusion in the systematic review (figure 1).^12^, ^27^, ^28^, ^29^, ^30^, ^31^, ^32^, ^33^, ^34^, ^35^, ^36^, ^37^, ^38^, ^39^, ^40^, ^41^, ^42^, ^43^, ^44^, ^45^, ^46^, ^47^, ^48^, ^49^, ^50^, ^51^, ^52^, ^53^, ^54^, ^55^, ^56^, ^57^, ^58^ These studies included a total of 19 096 individuals, with mean ages of 6·75–81·15 years. Among the eligible studies, 13 trials assessed antivirals for post-exposure prophylaxis against seasonal or pandemic influenza for populations with clear definition of exposure to influenza virus (eg, close contact with patients with laboratory-confirmed influenza or influenza-like illness),^12^, ^29^, ^34^, ^35^, ^36^, ^37^, ^38^, ^42^, ^43^, ^46^, ^48^, ^51^, ^55^ and 20 trials assessed antiviral prophylaxis for populations with unclear definition of exposure status or pre-exposure prophylaxis against influenza (eg, influenza season or outbreak in community or nursing home).^27^, ^28^, ^30^, ^31^, ^32^, ^33^, ^39^, ^40^, ^41^, ^44^, ^45^, ^47^, ^49^, ^50^, ^52^, ^53^, ^54^, ^56^, ^57^, ^58^ No trials were identified that assessed antivirals for prevention of human-to-human or animal-to-human transmission of novel influenza A viruses (zoonotic influenza). Of the 33 eligible trials, seven used a cluster (household or nursing home) as the randomisation unit^29^, ^34^, ^35^, ^46^, ^48^, ^54^, ^55^ and 26 randomly assigned individual participants.^12^, ^27^, ^28^, ^30^, ^31^, ^32^, ^33^, ^36^, ^37^, ^38^, ^39^, ^40^, ^41^, ^42^, ^43^, ^44^, ^45^, ^47^, ^49^, ^50^, ^51^, ^52^, ^53^, ^56^, ^57^, ^58^ Most trials used the following dosages of antivirals for post-exposure prophylaxis of influenza: zanamivir inhalation 10 mg or 20 mg once daily,^27^, ^35^, ^40^, ^41^, ^43^, ^48^, ^49^ oseltamivir 75 mg orally once or twice daily,^33^, ^39^, ^44^, ^45^, ^46^, ^55^ laninamivir inhalation 20 mg or 40 mg once daily,^37^, ^38^, ^51^ and baloxavir orally (single dose) with weight-based dosing (weight <10 kg: 1 mg/kg; 10 to <20 kg: 10 mg; 20 to <40 kg: 20 mg; ≥40 kg: 40 mg).^12^ Characteristics of the included studies are presented in the appendix (pp 19–27).

The included randomised controlled trials addressed six antivirals for prophylaxis of influenza: zanamivir, oseltamivir, laninamivir, baloxavir, amantadine, and rimantadine. Most of the studies were rated as having a low risk of bias. Risk of bias assessments for each trial are presented in the appendix (pp 28–35).

Of 13 trials that assessed the efficacy of antivirals for post-exposure prophylaxis against seasonal or pandemic influenza, ten trials studied antiviral prophylaxis of individuals started within 48 h of exposure to a symptomatic index patient,^12^, ^35^, ^37^, ^38^, ^42^, ^43^, ^46^, ^48^, ^51^, ^55^ one trial studied antiviral prophylaxis started as soon as possible after exposure to a symptomatic person with influenza,^34^ one trial assessed antiviral prophylaxis started up to 4 days after exposure,^36^ and one trial lacked clarity on the timing of antiviral prophylaxis after exposure to influenza.^29^ Among these trials of antiviral post-exposure prophylaxis of exposed individuals, eight trials also administered antiviral treatment to symptomatic index patients.^12^, ^34^, ^35^, ^37^, ^38^, ^46^, ^48^, ^51^ In four trials the index patients did not receive antiviral treatment,^42^, ^43^, ^48^, ^55^ and one trial did not specify whether index patients received antiviral treatment.^29^ The duration of antiviral post-exposure prophylaxis of exposed participants in the included trials ranged from 1 day^12^, ^51^ to 10 days.^29^, ^35^, ^46^, ^48^, ^53^ For 20 trials that assessed pre-exposure antiviral prophylaxis, the trials usually initiated antiviral prophylaxis during influenza season or during an institutional influenza outbreak for 14 days^27^, ^54^, ^57^ to 56 days.^47^

**Figure 2:**
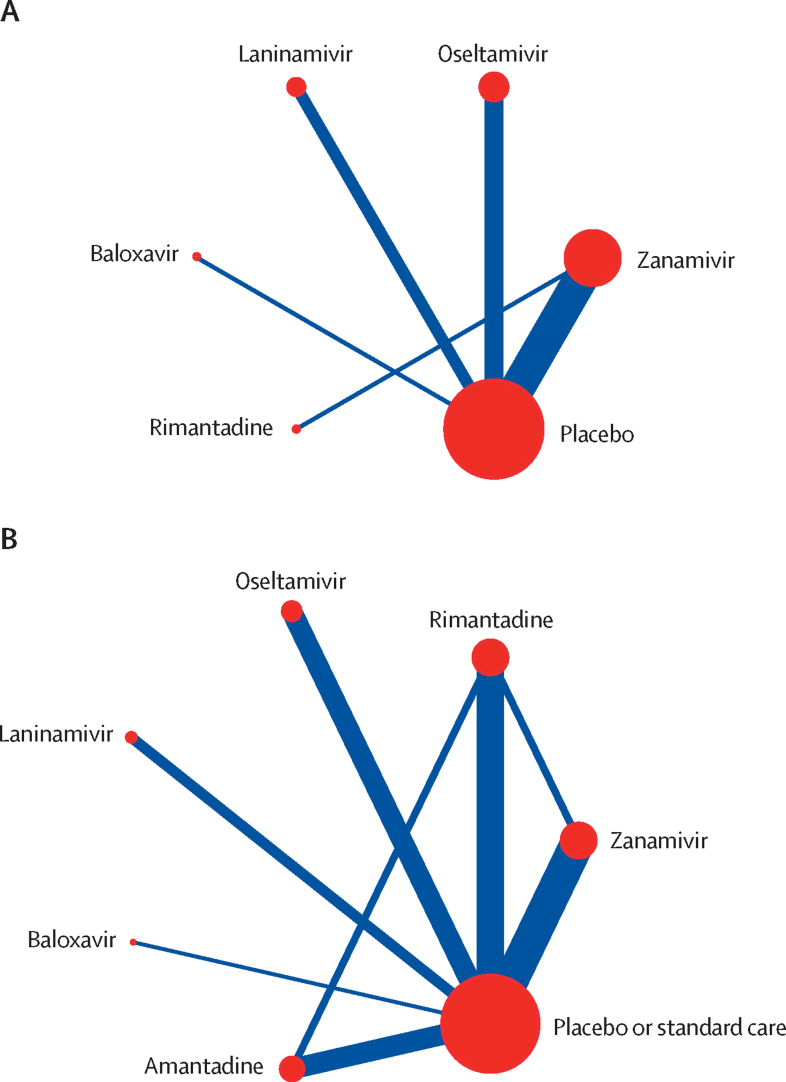
Network plots of trials included in meta-analyses (A) Trials evaluating laboratory-confirmed symptomatic influenza. (B) Trials evaluating laboratory-confirmed influenza. The size of the circle represents the number of participants. The connecting lines represent direct comparisons. The width of the line represents the number of trials.

Network plots of trials evaluating laboratory-confirmed symptomatic influenza and laboratory-confirmed influenza are shown in figure 2. Other network plots, forest plots, direct, indirect, and network estimates, and details of the GRADE assessment for each outcome are presented in the appendix (pp 36–82). Most of the outcomes had no serious concerns for incoherence (appendix pp 47–56, 83) or heterogeneity (appendix pp 84–85). No analyses suggested potential publication bias (appendix p 86).

19 trials with 15 645 individuals reported on laboratory-confirmed seasonal symptomatic influenza.^12^, ^27^, ^32^, ^33^, ^35^, ^36^, ^37^, ^38^, ^39^, ^40^, ^41^, ^42^, ^43^, ^44^, ^45^, ^48^, ^49^, ^51^, ^55^ All relative effect estimates come from direct comparisons between antivirals and placebo or standard care. In comparison with placebo or standard care, all antivirals except amantadine (no data) and rimantadine had similar RR estimates ranging from 0·35 to 0·43, with 95% CIs that did not include no effect (zanamivir: RR 0·35, 95% CI 0·25–0·50; oseltamivir: 0·40, 0·26–0·62; laninamivir: 0·43, 0·30–0·63; baloxavir: 0·43, 0·23–0·79; appendix pp 40–82), indicating a reduction in the risk of symptomatic influenza. The RR for rimantadine for symptomatic influenza A virus infection was 0·76 (0·28–2·06; appendix pp 40–82).

**Figure 3:**
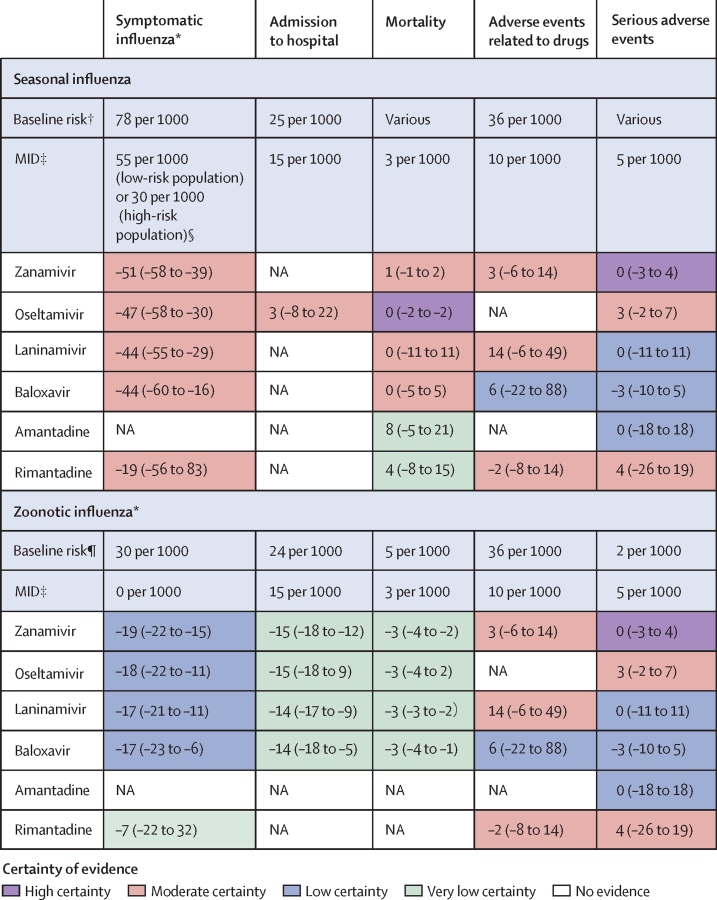
Summary of effects of interventions compared with placebo Absolute risk estimates for each outcome are presented in comparison with standard care or placebo. Numbers in the coloured cells are point estimates per 1000 people and their 95% CIs. MID=minimal important difference. NA=not applicable due to no available data. *For low-risk populations, these antivirals probably have little to no important effect on symptomatic influenza; however, zanamivir, oseltamivir, laninamivir, and baloxavir probably have an important effect on symptomatic influenza in high-risk populations. †Baseline risk estimates for for symptomatic influenza, admission to hospital, and adverse events related to drugs were from the control (placebo or standard care) group of eligible studies; various indicates that no single estimate was used and the absolute risk reductions were calculated as the risk differences. ‡MID values were established based on discussion with the WHO guideline development panel; they were used to support judgements of imprecision. §Low-risk and high-risk populations were defined based on the 2022 WHO influenza guidelines (appendix p 18). ¶Baseline risk estimates for symptomatic influenza, admission to hospital, mortality, and serious adverse events were informed by the WHO guideline panel discussion; baseline risk estimates for adverse events related to drugs were from the control group of eligible studies.

For populations at low risk of severe influenza, the effect of zanamivir, oseltamivir, laninamivir, baloxavir, and rimantadine in reducing symptomatic influenza fell below the threshold of importance as defined by MIDs (RR estimates of 0·35–0·76 and absolute risk reductions from 19 fewer to 51 fewer per 1000; appendix pp 40–82). For populations at high risk of severe influenza, zanamivir, oseltamivir, laninamivir, and baloxavir probably have important effects in reducing symptomatic influenza (moderate certainty; figure 3 and appendix pp 57–82). By contrast, rimantadine probably has little or no effect on symptomatic influenza A virus infection (moderate certainty; figure 3 and appendix pp 57–82). Whether amantadine reduces the development of symptomatic influenza A virus infection is very uncertain (figure 3).

33 trials with 19 096 individuals reported influenza virus infection regardless of symptoms.^12^, ^27^, ^28^, ^29^, ^30^, ^31^, ^32^, ^33^, ^34^, ^35^, ^36^, ^37^, ^38^, ^39^, ^40^, ^41^, ^42^, ^43^, ^44^, ^45^, ^46^, ^47^, ^48^, ^49^, ^50^, ^51^, ^52^, ^53^, ^54^, ^55^, ^56^, ^57^, ^58^ Compared with placebo or standard care, all antivirals had a similar effect showing a decrease in the risk of influenza virus infection, with RR estimates from 0·46 to 0·58 and absolute risk reductions from 96 fewer to 74 fewer per 1000 people (appendix pp 40–82). Oseltamivir, laninamivir, and baloxavir all probably decrease the risk of influenza virus infection, and amantadine probably decreases the risk of influenza A virus infection (moderate certainty; appendix pp 40–82). Zanamivir might decrease the risk of influenza virus infection and rimantadine might decrease the risk of influenza A virus infection (low certainty; appendix pp 40–82). Antivirals reduced symptomatic influenza, driving the results for all influenza (symptomatic and asymptomatic infection). By contrast, antivirals probably have little or no effect on prevention of asymptomatic influenza virus infection (moderate certainty; appendix pp 40–82).

Four studies with 3434 participants reported admission to hospital for the comparison of oseltamivir and placebo for seasonal influenza.^33^, ^44^, ^45^, ^55^ Oseltamivir probably has little or no effect on admission to hospital (moderate certainty; figure 3 and appendix pp 40–82). No data were available on admission to hospital for other antiviral drugs (figure 3).

For all-cause mortality, we calculated the risk difference because the baseline event rate was less than 1%. 15 studies with 10 068 participants provided evidence that zanamivir, oseltamivir, laninamivir, and baloxavir all probably have little or no effect on all-cause mortality,^12^, ^27^, ^32^, ^33^, ^40^, ^41^, ^42^, ^43^, ^44^, ^45^, ^46^, ^47^, ^51^, ^52^, ^55^ with absolute risk reductions from zero fewer to one more per 1000 patients (high or moderate certainty; figure 3 and appendix pp 40–82). Whether amantadine or rimantadine reduces all-cause mortality from influenza A virus infection is very uncertain (figure 3 and appendix pp 40–82).

13 trials with 10 838 participants reported adverse events related to drugs.^12^, ^27^, ^32^, ^35^, ^36^, ^37^, ^38^, ^40^, ^41^, ^42^, ^43^, ^49^, ^51^ Zanamivir, laninamivir, and rimantadine all probably result in fewer drug-related adverse events, with RRs ranging from 1·01 to 1·40 and absolute risks ranging from three more to 14 more per 1000 people (moderate certainty; figure 3 and appendix pp 40–82). Compared with placebo, baloxavir might have little or no effect on drug-related adverse events (six more per 1000 people, 95% CI 22 fewer to 88 more, low certainty; figure 3 and appendix pp 40–82).

16 studies with 11 755 participants reported serious adverse events.^12^, ^27^, ^32^, ^33^, ^35^, ^39^, ^40^, ^41^, ^42^, ^43^, ^44^, ^45^, ^49^, ^50^, ^51^, ^55^ Compared with placebo, all antivirals might have little or no effect on serious adverse events, with absolute risk increases ranging from zero to four per 1000 people (appendix pp 40–82). The certainty of evidence was high for zanamivir, moderate for oseltamivir and rimantadine, and low for laninamivir, baloxavir, and amantadine (figure 3 and appendix pp 57–82).

No randomised controlled trials were identified for antiviral post-exposure prophylaxis of individuals exposed to symptomatic people or to animals infected with novel influenza A viruses (zoonotic influenza) associated with severe disease and high mortality in infected humans. Therefore, we considered indirect evidence from trials of antiviral post-exposure prophylaxis for seasonal influenza. For populations exposed to novel influenza A viruses associated with severe disease and high mortality in infected humans, zanamivir, oseltamivir, laninamivir, and baloxavir might have an important effect in reducing development of symptomatic zoonotic influenza (low certainty; figure 3 and appendix pp 57–82). Whether amantadine or rimantadine reduce the development of symptomatic zoonotic influenza is very uncertain (figure 3 and appendix pp 57–82). We infer that when using antivirals for post-exposure prophylaxis against zoonotic influenza, the risk of adverse events is similar to when using antivirals for post-exposure prophylaxis of seasonal influenza (figure 3 and appendix pp 57–82).

The evidence for seasonal influenza raises the possibility that zanamivir, oseltamivir, laninamivir, and baloxavir might have important effects on admission to hospital and all-cause mortality for people exposed to novel influenza A viruses associated with severe disease and high mortality in infected humans. However, the certainty of evidence is very low (figure 3, appendix pp 57–82).

Our sensitivity analyses showed similar results to the primary analyses (appendix pp 87–110). The effects of zanamivir for preventing laboratory-confirmed influenza B were similar to the effects for preventing laboratory-confirmed influenza A (appendix pp 103–04). No statistically significant subgroup effects were found between different age groups and influenza vaccine statuses on symptomatic influenza (interaction p>0·10; appendix pp 111–13). When analysing antiviral treatment in subgroups of index patients, a statistically significant interaction (p=0·010) was found, but the subgroup effect was low according to ICEMAN criteria (appendix pp 114–16).

## Discussion

In this systematic review and network meta-analysis of randomised controlled trials of antiviral prophylaxis of influenza compared with placebo or standard care, we found that oseltamivir, laninamivir, zanamivir, and baloxavir can each reduce the risk of symptomatic seasonal influenza in individuals at high risk who have been exposed to a symptomatic close contact with influenza, when antiviral prophylaxis is initiated promptly (eg, within 48 h) after exposure. However, post-exposure prophylaxis with these antivirals might have little or no important effect on reducing symptomatic seasonal influenza in low-risk populations. For populations exposed to novel influenza A viruses associated with severe disease in infected humans, prompt administration of post-exposure prophylaxis with oseltamivir, zanamivir, laninamivir, or baloxavir might reduce the risk of symptomatic zoonotic influenza. We did not find convincing evidence to suggest that antiviral post-exposure prophylaxis provides important reductions in the risk of admission to hospital or all-cause mortality due to seasonal influenza. These antivirals might also have no important effects on adverse events or severe adverse events related to the drugs.

This network meta-analysis assessed antivirals for post-exposure prophylaxis of influenza. We specified explicit eligibility criteria, conducted a comprehensive literature search for eligible studies, and performed duplicate selection, data extraction, and risk of bias assessment. We assessed the effects of antivirals in preventing seasonal influenza for both low-risk and high-risk populations, and, based on discussion with the WHO guideline panel, incorporated data modelling for prevention of zoonotic influenza. The WHO panel provided MID values of importance to patients that we used to interpret the results and guide the ratings for imprecision. We performed within-trial subgroup analyses to explore possible effect modification according to age and influenza vaccination status and performed sensitivity analyses to assess risk of bias, missing data, exposure status to influenza viruses, and ICC values, all of which yielded results similar to our primary analyses.

Our review has limitations. First, data were not available to assess some outcomes identified by the WHO guidelines panel as important, including length of hospitalisation, ICU admission, invasive mechanical ventilation, and influenza disease severity. Second, the mean age of participants in the included studies ranged from 6·75 years to 81·15 years, including many patients at high risk, especially adults aged 65 years and older. However, data were scarce for pregnant people and infants younger than 1 year. Third, although studies varied in the route of drug administration, dosage, and duration of post-exposure prophylaxis, we combined them in the network meta-analysis. Fourth, we included three trials in which the index case had influenza-like illness because the results were unchanged when these studies were excluded in sensitivity analyses. Fifth, because no randomised controlled trials have been conducted of antivirals for post-exposure prophylaxis to prevent symptomatic zoonotic influenza, we used indirect evidence from randomised controlled trials of seasonal influenza as the evidence base for zoonotic influenza. Also, because the overall mortality for zoonotic influenza is unknown, we estimated the case fatality proportion for zoonotic influenza to be 30%, based on sporadic human infections with different novel influenza A viruses associated with severe disease. This overestimates the mortality for other avian influenza A virus infections and swine influenza A virus infections of humans that are associated with lower disease severity. Finally, the available data for included trials of antivirals allowed us only to assess the antiviral effects of zanamivir separately for the outcomes of influenza type A and influenza type B. The effects of zanamivir were similar for both influenza types. Given that for post-exposure prophylaxis trials of other antiviral drugs, most participants studied were exposed to index cases infected with influenza A viruses, the effects of other antivirals for post-exposure prophylaxis against influenza B remain uncertain.

Previous systematic reviews have suggested that prophylaxis with antivirals is effective for preventing symptomatic influenza but have not assessed the certainty of the evidence.^9^, ^10^, ^11^, ^59^, ^60^, ^61^, ^62^, ^63^ Our findings of no statistically significant effect on asymptomatic influenza virus infection,^10^, ^11^ mortality^59^ or serious adverse events^10^ are consistent with those of previous systematic reviews. Previous reviews assessed zanamivir, oseltamivir, and laninamivir but did not include one more recent randomised controlled trial for baloxavir from 2020.^12^ Our systematic review is updated and is more comprehensive than previous reviews, provides ratings of the certainty of evidence, and is the first to specify MIDs and thus to explicitly address the importance to patients of the intervention effects.

Our systematic review provides evidence for the clinical benefit and safety of antiviral prophylaxis to prevent symptomatic seasonal influenza in individuals who are at high risk for severe influenza when started within 48 h of exposure to a symptomatic person with influenza.

There are a number of important populations for which data from randomised controlled trials on antiviral post-exposure prophylaxis remain scarce, including for exposed pregnant people, infants (age <1 year), and people with kidney dysfunction, liver disease, and other chronic medical conditions. Other knowledge gaps include understanding the risk of infection and severe zoonotic influenza, and the benefit of antiviral post-exposure prophylaxis in people exposed to animals or humans infected with novel influenza A viruses associated with severe disease and high mortality.

In conclusion, following exposure to people with seasonal influenza, prompt initiation of post-exposure prophylaxis with oseltamivir, zanamivir, laninamivir, or baloxavir probably provides an important reduction in the risk of symptomatic influenza in individuals who are at high risk for severe influenza. Similarly, based on indirect evidence for seasonal influenza, prompt initiation of post-exposure prophylaxis with these antivirals might also provide important reductions in the risk of symptomatic zoonotic influenza.

### Contributors

### Data sharing

All data included were derived from publicly available documents cited in the references. Extracted data are available upon request to the corresponding author.

## Declaration of interests

We declare no competing interests.
