# Supplementary material for: Antivirals for post-exposure prophylaxis of influenza: a systematic review and network meta-analysis
Source: Lancet. 2024 Aug 24;404(10454):764–72. doi: 10.1016/S0140-6736(24)01357-6 (PMC11369964; doi:10.1016/S0140-6736(24)01357-6)
Supplement: Supplementary appendix [file mmc1.pdf]

THE LANCET

Supplementary appendix

This appendix formed part of the original submission and has been peer reviewed. We post it as supplied by the authors.

Supplement to: Zhao Y, Gao Y, Guyatt G, et al. Antivirals for post-exposure prophylaxis of influenza: a systematic review and network meta-analysis. *Lancet* 2024; **404:** 764–72.

Antiviral therapies for prophylaxis against influenza: a systematic review and network meta-analysis

Content of table

Appendix 1 Search strategy 7

Appendix 2 Risk of bias tool 15

Appendix 3 Prior hypotheses and the anticipated direction of effects for subgroup analysis... 16

Appendix 4 Methods for rating the certainty of evidence. 17

Appendix 5 Character population at high risk of severe disease. 18

Appendix 6 Basic characteristics of eligible studies.. 19

Appendix 7 Risk of bias for eligible studies 28

Appendix 8 Network plots. 36

Figure 1 Network plot of trials evaluating antivirals prophylaxis against laboratory-confirmed asymptomatic influenza. 36

Figure 2 Network plot of trials evaluating antivirals prophylaxis against all-cause mortality. 37

Figure 3 Network plot of trials evaluating antivirals prophylaxis against serious adverse events. 38

Figure 4 Network plot of trials evaluating antivirals prophylaxis against adverse events related to drugs. 39

Appendix 9 Forest plots 40

Figure 1 Forest plot for antivirals prophylaxis against laboratory-confirmed symptomatic influenza. 40

Figure 2 Forest plot for antivirals prophylaxis against laboratory-confirmed influenza.. 41

Figure 3 Forest plot for antivirals prophylaxis against laboratory-confirmed asymptomatic influenza. 42

Figure 4 Forest plot for antivirals prophylaxis against admission to hospital. 43

Figure 5 Forest plot for antivirals prophylaxis against all-cause mortality. 44
Figure 6 Forest plot for antivirals prophylaxis against serious adverse events. ..45
Figure 7 Forest plot for antivirals prophylaxis against adverse events related to drugs. 46
Appendix 10 Direct, indirect, and network treatment estimates for each outcome. 47
Table 1 Direct, indirect, and network treatment estimates for laboratory-confirmed symptomatic influenza. 47
Table 2 Direct, indirect, and network treatment estimates for laboratory-confirmed influenza. 49
Table 3 Direct, indirect, and network treatment estimates for laboratory-confirmed asymptomatic influenza. 51
Table 4 Direct, indirect, and network treatment estimates for mortality. 52
Table 5 Direct, indirect, and network treatment estimates for adverse events related to drugs. 54
Table 6 Direct, indirect, and network treatment estimates for serious adverse events. 55
Appendix 11 Network estimates with GRADE ratings 57
Table 1 Network estimates with GRADE ratings for laboratory-confirmed symptomatic influenza (seasonal influenza) 57
Table 2 Network estimates with GRADE ratings for laboratory-confirmed influenza (seasonal influenza) 59
Table 3 Network estimates with GRADE ratings for laboratory-confirmed asymptomatic influenza (seasonal influenza) 60
Table 4 Network estimates with GRADE ratings for all-cause mortality (seasonal influenza) 61
Table 5 Network estimates with GRADE ratings for adverse events related to drugs (seasonal influenza and zoonotic influenza) 62
Table 6 Network estimates with GRADE ratings for serious adverse events (seasonal influenza and zoonotic influenza) 63

Table 7 Network estimates with GRADE ratings for laboratory-confirmed symptomatic influenza (zoonotic influenza)..64
Table 8 Network estimates with GRADE ratings for admission to hospital (zoonotic influenza) 65
Table 9 Network estimates with GRADE ratings for all-cause mortality (zoonotic influenza) 66
Appendix 12 Summary of findings table for antivirals versus placebo/standard care or placebo. 67
Table 1 Summary of findings table for zanamivir versus placebo or standard care for prophylaxis against seasonal influenza 67
Table 2 Summary of findings table for oseltamivir versus placebo for prophylaxis against seasonal influenza..69
Table 3 Summary of findings table for laninamivir versus placebo for prophylaxis against seasonal influenza..71
Table 4 Summary of findings table for baloxavir versus placebo for prophylaxis against seasonal influenza..73
Table 5 Summary of findings table for amantadine versus placebo for prophylaxis against seasonal influenza..75
Table 6 Summary of findings table for rimantadine versus placebo for prophylaxis against seasonal influenza..76
Table 7 Summary of findings table for zanamivir versus placebo for prophylaxis against zoonotic influenza...77
Table 8 Summary of findings table for oseltamivir versus placebo for prophylaxis against zoonotic influenza..78
Table 9 Summary of findings table for laninamivir versus placebo for prophylaxis against zoonotic influenza..79
Table 10 Summary of findings table for baloxavir versus placebo for prophylaxis against zoonotic influenza...80

Table 11 Summary of findings table for amantadine versus placebo for prophylaxis against zoonotic influenza 81
Table 12 Summary of findings table for rimantadine versus placebo for prophylaxis against zoonotic influenza 82
Appendix 13 Assessment of global inconsistency 83
Appendix 14 Assessment of between-study heterogeneity. 84
Appendix 15 Funnel plot 86
Figure 1 Funnel plot zanamivir vs placebo/ standard care for laboratory-confirmed influenza (Harbord test p = 0.77). 86
Appendix 16 Sensitivity analysis by only including the trials with lower risk of bias 87
Figure 1 Sensitivity analysis for antivirals prophylaxis against laboratory-confirmed symptomatic influenza by only including the trials with lower risk of bias. 87
Figure 2 Sensitivity analysis for antivirals prophylaxis against laboratory-confirmed influenza by only including the trials with lower risk of bias. 88
Figure 3 Sensitivity analysis for antivirals prophylaxis against mortality by only including the trials with lower risk of bias 89
Figure 4 Sensitivity analysis for antivirals prophylaxis against serious adverse events by only including the trials with lower risk of bias. 90
Figure 5 Sensitivity analysis for antivirals prophylaxis against adverse events related to drugs by only including the trials with lower risk of bias 91
Appendix 17 Plausible worst-case sensitivity analyses 92
Figure 1 Plausible worst-case sensitivity analyses for antivirals prophylaxis against laboratory-confirmed symptomatic influenza. 92
Figure 2 Plausible worst-case sensitivity analyses for antivirals prophylaxis against laboratory-confirmed influenza. 93
Appendix 18 Sensitivity analysis according to the evidence of exposure to influenza 94

Figure 1 Sensitivity analysis for antivirals prophylaxis against laboratory-confirmed symptomatic influenza by including the trials with clear evidence of exposure to influenza. 94
Figure 2 Sensitivity analysis for antivirals prophylaxis against laboratory-confirmed symptomatic influenza by excluding the trials with clear evidence of exposure to influenza. 95
Figure 3 Sensitivity analysis for antivirals prophylaxis against laboratory-confirmed influenza by including the trials with clear evidence of exposure to influenza. 96
Figure 4 Sensitivity analysis for antivirals prophylaxis against laboratory-confirmed influenza by including the trials with less evidence of exposure to influenza. 97
Figure 5 Sensitivity analysis for antivirals prophylaxis against laboratory-confirmed asymptomatic influenza by including the trials with clear evidence of exposure to influenza. 98
Figure 6 Sensitivity analysis for antivirals prophylaxis against laboratory-confirmed asymptomatic influenza by excluding the trials with clear evidence of exposure to influenza. 99
Figure 7 Sensitivity analysis for antivirals prophylaxis against laboratory-confirmed symptomatic influenza by only including the trials with close contact with the index with confirmed influenza. 100
Figure 8 Sensitivity analysis for antivirals prophylaxis against laboratory-confirmed influenza by only including the trials with close contact with the index with confirmed influenza. 101
Figure 9 Sensitivity analysis for antivirals prophylaxis against laboratory-confirmed asymptomatic influenza by only including the trials with close contact with the index with confirmed influenza. 102
Appendix 19 Sensitivity analysis includes only the trials that reported laboratory-confirmed influenza A and influenza B separately. 103

Figure 1 Analysis for zanamivir prophylaxis against laboratory-confirmed influenza A (includes trials that only reported laboratory-confirmed influenza A). 103
Figure 2 Analysis for zanamivir prophylaxis against laboratory-confirmed influenza B (includes trials that only reported laboratory-confirmed influenza B). 104
Appendix 20 Sensitivity analysis by using a higher ICC (0.10) to adjust cluster design effect. 105
Figure 1 Sensitivity analysis for antivirals prophylaxis against laboratory-confirmed symptomatic influenza (ICC = 0.1). 105
Figure 2 Sensitivity analysis for antivirals prophylaxis against laboratory-confirmed influenza (ICC = 0.1). 106
Figure 3 Sensitivity analysis for antivirals prophylaxis against laboratory-confirmed asymptomatic influenza (ICC = 0.1). 107
Figure 4 Sensitivity analysis for antivirals prophylaxis against all-cause mortality (ICC = 0.1). 108
Figure 5 Sensitivity analysis for antivirals prophylaxis against serious adverse events (ICC = 0.1). 109
Figure 6 Sensitivity analysis for antivirals prophylaxis against adverse events related to drugs (ICC = 0.1). 110
Appendix 21 Subgroup analysis 111
Figure 1 Within-subgroup analysis of laboratory-confirmed influenza according to age (zanamivir versus placebo) 111
Figure 2 Within-subgroup analysis of laboratory-confirmed influenza according to vaccination status (zanamivir versus placebo) 112
Figure 3 Within-subgroup analysis of laboratory-confirmed influenza according to the status of index patients treatment (zanamivir versus placebo) 113
Appendix 22: Credibility assessment of subgroup analysis 114
Text 1 Credibility assessment of treatment of index patients 114

Appendix 1 Search strategy

Ovid MEDLINE(R) ALL

1 exp Influenza, Human/ 58097
2 exp Influenza A virus/ 49553
3 exp Influenza B virus/ 4644
4 exp Influenzavirus C/ 363
5 (Influenza or flu or H1N1 or PH1N1 or H3N2 or AH1N1 or AH3N2 or H5N1 or H7N9).mp. [mp=title, book title, abstract, original title, name of substance word, subject heading word, floating sub-heading word, keyword heading word, organism supplementary concept word, protocol supplementary concept word, rare disease supplementary concept word, unique identifier, synonyms, population supplementary concept word, anatomy supplementary concept word] 140078
6 or/1-5 140084
7 Antiviral agents/ 100530
8 Antiviral*.tw. 118002
9 (neuraminidase inhibitor* or NA inhibitor*).tw. 2443
10 Oseltamivir/ or Zanamivir/ 3724
11 (oseltamivir or tamiflu or GS 4104 or GS4104 or GS-4104 or GS 4071 or GS4071 or GS-4071 or zanamivir or relenza or GG 167 or GG167 or GG-167 or CS-8958 or Dectova or Laninamivir or R-125489 or R125489 or R 125489 or Inavir or peramivir or BCX 1812 or BCX1812 or BCX-1812 or RWJ 270201 or RWJ270201 or RWJ-270201 or Rapivab or rapiacta).ti,ab. 5041
12 Viral Polymerase Complex Inhibitor*.tw. 0
13 (Favipiravir or T-705 or Avigan or FabiFlu or Pimodivir or VX-787 or JNJ-63623872 or AL-794 or ALS-033719 or ZSP1273 or Enisamium iodide or FAV00A or TG-1000 or GP681).ti,ab. 1339
14 matrix protein 2 ion channel inhibitor*.tw. 2
15 (Radavirsen or AVI-7100).ti,ab. 2
16 cap-dependent endonuclease inhibitor*.tw.38
17 ("Baloxavir marboxil" or Baloxavir or S-033188 or Xofluza).ti,ab. 274
18 (Umifenovir or Arbidol or Arbidole).ti,ab. 430
19 Amantadine/ or Rimantadine/ 4282
20 (Amantadine or Symmetrel or Symetrel or Rimantadine or Flumadine or Roflual).ti,ab. 4429
21 or/7-20 178589
22 6 and 21 18091
23 randomized controlled trial.pt. 599492
24 controlled clinical trial.pt. 95418
25 randomized.ab. 616237
26 placebo.ab. 241273
27 drug therapy.fs. 2621016
28 randomly.ab. 416158
29 trial.ti. 292288
30 groups.ab. 2566299

31 or/23-30 5605395
32 (animals not (humans and animals)).sh. 5119274
33 31 not 324891800
34 22 and 33 6314

Ovid Embase
1 exp Influenza/ or Influenza virus/ 121021
2 exp Influenza A virus/ or exp Influenza A virus/ 20306
3 exp Influenza B/ or exp Influenza B virus/ 6376
4 exp Influenza C/ or exp Influenza C virus/ 240
5 (Influenza or flu or H1N1 or PH1N1 or H3N2 or AH1N1 or AH3N2 or H5N1 or H7N9).mp. [mp=title, abstract, heading word, drug trade name, original title, device manufacturer, drug manufacturer, device trade name, keyword heading word, floating subheading word, candidate term word] 203693
6 or/1-5 203693
7 Antivirus agent/ 97344
8 Antiviral*.tw. 155192
9 (neuraminidase inhibitor* or NA inhibitor*).tw. 3010
10 Sialidase inhibitor/ or Oseltamivir/ or Zanamivir/ or Laninamivir/ or Peramivir/ 17267
11 (oseltamivir or tamiflu or "GS 4104" or GS4104 or GS-4104 or "GS 4071" or GS4071 or GS-4071 or zanamivir or relenza or "GG 167" or GG167 or GG-167 or CS-8958 or Dectova or Laninamivir or R-125489 or R125489 or "R 125489" or Inavir or peramivir or "BCX 1812" or BCX1812 or BCX-1812 or "RWJ 270201" or RWJ270201 or RWJ-270201 or Rapivab or rapiacta).ti,ab. 7147
12 Viral Polymerase Complex Inhibitor*.tw. 0
13 Favipiravir/ or Pimodivir/ or (Favipiravir or T-705 or Avigan or FabiFlu or Pimodivir or VX-787 or JNJ-63623872 or AL-794 or ALS-033719 or ZSP1273 or Enisamium iodide or FAV00A or TG-1000 or GP681).ti,ab. 5003
14 matrix protein 2 ion channel inhibitor*.tw. 1
15 Radavirsen/ or (Radavirsen or AVI-7100).ti,ab. 15
16 cap-dependent endonuclease inhibitor*.tw.49
17 Baloxavir marboxil/ or ("Baloxavir marboxil" or Baloxavir or S-033188 or Xofluza).ti,ab. 538
18 Umifenovir/ or (Umifenovir or Arbidol or Arbidole).ti,ab. 1836
19 Amantadine/ or Rimantadine/ 17849
20 (Amantadine or Symmetrel or Symetrel or Rimantadine or Flumadine or Roflual).ti,ab. 5729
21 or/7-20 229508
22 6 and 21 29821
23 Randomized controlled trial/ 782084
24 Controlled clinical study/ 470919
25 random$.ti,ab. 1972415

26 randomization/ 98334
27 intermethod comparison/ 300395
28 placebo.ti,ab. 364870
29 (compare or compared or comparison).ti. 604471
30 ((evaluated or evaluate or evaluating or assessed or assess) and (compare or compared or comparing or comparison)).ab. 2773393
31 (open adj label).ti,ab. 108525
32 ((double or single or doubly or singly) adj (blind or blinded or blindly)).ti,ab. 273606
33 double blind procedure/ 210398
34 parallel group$1.ti,ab. 32087
35 (crossover or cross over).ti,ab. 124492
36 ((assign$ or match or matched or allocation) adj5 (alternate or group$1 or intervention$1 or patient$1 or subject$1 or participant$1)).ti,ab. 414740
37 (assigned or allocated).ti,ab. 490042
38 (controlled adj7 (study or design or trial)).ti,ab. 449985
39 (volunteer or volunteers).ti,ab. 282271
40 human experiment/ 641156
41 trial.ti. 400728
42 or/23-41 6316622
43 (random$ adj sampl$ adj7 ("cross section$" or questionnaire$1 or survey$ or database$1)).ti,ab. not (comparative study/ or controlled study/ or randomi?ed controlled.ti,ab. or randomly assigned.ti,ab.) 9594
44 Cross-sectional study/ not (randomized controlled trial/ or controlled clinical study/ or controlled study/ or randomi?ed controlled.ti,ab. or control group$1.ti,ab.) 360295
45 (((case adj control$) and random$) not randomi?ed controlled).ti,ab. 21530
46 (Systematic review not (trial or study)).ti. 258944
47 (nonrandom$ not random$).ti,ab. 18933
48 "Random field$".ti,ab. 2959
49 (random cluster adj3 sampl$).ti,ab. 1578
50 (review.ab. and review.pt.) not trial.ti. 1128667
51 "we searched".ab. and (review.ti. or review.pt.) 49232
52 "update review".ab. 136
53 (databases adj4 searched).ab. 62280
54 (rat or rats or mouse or mice or swine or porcine or murine or sheep or lambs or pigs or piglets or rabbit or rabbits or cat or cats or dog or dogs or cattle or bovine or monkey or monkeys or trout or marmoset$1).ti. and animal experiment/ 1219722
55 Animal experiment/ not (human experiment/ or human/) 2561951
56 or/43-55 4337577
57 42 not 565573353
58 22 and 57 3027

OVID EBM Reviews - Cochrane Central Register of Controlled Trials

1 exp Influenza, Human/ 3241
2 exp Influenza A virus/ 1021
3 exp Influenza B virus/ 320
4 exp Influenzavirus C/ 0
5 (Influenza or flu or H1N1 or PH1N1 or H3N2 or AH1N1 or AH3N2 or H5N1 or H7N9).mp. [mp=title, original title, abstract, floating sub-heading word, mesh headings, heading words, keyword] 10694
6 or/1-5 10694
7 Antiviral agents/ 4914
8 Antiviral*.tw. 6923
9 (neuraminidase inhibitor* or NA inhibitor*).tw. 145
10 Oseltamivir/ or Zanamivir/ 348
11 (oseltamivir or tamiflu or "GS 4104" or GS4104 or GS-4104 or "GS 4071" or GS4071 or GS-4071 or zanamivir or relenza or "GG 167" or GG167 or GG-167 or CS-8958 or Dectova or Laninamivir or R-125489 or R125489 or "R 125489" or Inavir or peramivir or "BCX 1812" or BCX1812 or BCX-1812 or "RWJ 270201" or RWJ270201 or RWJ-270201 or Rapivab or rapiacta).ti,ab. 700
12 Viral Polymerase Complex Inhibitor*.tw. 0
13 (Favipiravir or T-705 or Avigan or FabiFlu or Pimodivir or VX-787 or JNJ-63623872 or AL-794 or ALS-033719 or ZSP1273 or Enisamium iodide or FAV00A or TG-1000 or GP681).ti,ab. 310
14 matrix protein 2 ion channel inhibitor*.tw. 0
15 (Radavirsen or AVI-7100).ti,ab. 3
16 cap-dependent endonuclease inhibitor*.tw.4
17 ("Baloxavir marboxil" or Baloxavir or S-033188 or Xofluza).ti,ab. 59
18 (Umifenovir or Arbidol or Arbidole).ti,ab. 71
19 Amantadine/ or Rimantadine/ 485
20 (Amantadine or Symmetrel or Symetrel or Rimantadine or Flumadine or Roflual).ti,ab. 913
21 or/7-20 11661
22 6 and 21 1277
23 randomized controlled trial.pt. 0
24 controlled clinical trial.pt. 0
25 randomized.ab. 680359
26 placebo.ab. 332394
27 drug therapy.fs. 261711
28 randomly.ab. 309521
29 trial.ti. 410998
30 groups.ab. 576574
31 or/23-30 1361436
32 (animals not (humans and animals)).sh. 2671
33 31 not 321359098
34 22 and 33 1006

Ovid Global Health

1 exp Influenza/ or Influenza viruses/ 40928

2 exp Influenza A virus/ or exp Influenza A virus/ 22916

3 exp Influenza B/ or exp Influenza B virus/ 3545

4 exp Influenza C/ or exp Influenza C virus/ 180

5 (Influenza or flu or H1N1 or PH1N1 or H3N2 or AH1N1 or AH3N2 or H5N1 or H7N9).mp. [mp=abstract, title, original title, heading words, cabicodes words] 50678

6 or/1-5 50678

7 Antiviral agents/ 115951

8 Antiviral*.tw. 138051

9 (neuraminidase inhibitor* or NA inhibitor*).tw. 1090

10 Sialidase inhibitors/ or Oseltamivir/ or Zanamivir/ or Laninamivir/ or Peramivir/ 1965

11 (oseltamivir or tamiflu or "GS 4104" or GS4104 or GS-4104 or "GS 4071" or GS4071 or GS-4071 or zanamivir or relenza or "GG 167" or GG167 or GG-167 or CS-8958 or Dectova or Laninamivir or R-125489 or R125489 or "R 125489" or Inavir or peramivir or "BCX 1812" or BCX1812 or BCX-1812 or "RWJ 270201" or RWJ270201 or RWJ-270201 or Rapivab or rapiacta).ti,ab. 2460

12 Viral Polymerase Complex Inhibitor*.tw. 0

13 Favipiravir/ or (Favipiravir or T-705 or Avigan or FabiFlu or Pimodivir or VX-787 or JNJ-63623872 or AL-794 or ALS-033719 or ZSP1273 or Enisamium iodide or FAV00A or TG-1000 or GP681).ti,ab. 682

14 matrix protein 2 ion channel inhibitor*.tw. 0

15 (Radavirsen or AVI-7100).ti,ab. 1

16 cap-dependent endonuclease inhibitor*.tw. 17

17 ("Baloxavir marboxil" or Baloxavir or S-033188 or Xofluza).ti,ab. 113

18 (Umifenovir or Arbidol or Arbidole).ti,ab. 185

19 Amantadine/ or Rimantadine/ 551

20 (Amantadine or Symmetrel or Symetrel or Rimantadine or Flumadine or Roflual).ti,ab. 708

21 or/7-20 138737

22 6 and 21 8017

23 exp randomized controlled trials/ 61880

24 (randomized controlled trial or random* or blind* or placebo*).mp. [mp=abstract, title, original title, heading words, cabicodes words] 322171

25 23 or 24 322171

26 22 and 25 590

CINAHL

#	Query	Limiters/Expanders	Results

S36	S23 AND S26 AND S35	Search modes - Boolean/Phrase	391
S35	S27 OR S28 OR S29 OR S30 OR S31 OR S32 OR S33 OR S34	Search modes - Boolean/Phrase	27,192
S34	TI (matrix protein 2 ion channel inhibitor* OR Radavirsen or AVI-7100 OR cap-dependent endonuclease inhibitor* OR "Baloxavir marboxil" or Baloxavir or S-033188 or Xofluza OR Umifenovir or Arbidol or Arbidole OR Amantadine or Symmetrel or Symetrel or Rimantadine or Flumadine or Roflual) OR AB (matrix protein 2 ion channel inhibitor* OR Radavirsen or AVI-7100 OR cap-dependent endonuclease inhibitor* OR "Baloxavir marboxil" or Baloxavir or S-033188 or Xofluza OR Umifenovir or Arbidol or Arbidole OR Amantadine or Symmetrel or Symetrel or Rimantadine or Flumadine or Roflual)	Search modes - Boolean/Phrase	711
S33	(MH "Amantadine")	Search modes - Boolean/Phrase	587
S32	TI (Viral Polymerase Complex Inhibitor* OR Favipiravir or T-705 or Avigan or FabiFlu or Pimodivir or VX-787 or JNJ-63623872 or AL-794 or ALS-033719 or ZSP1273 or Enisamium iodide or FAV00A or TG-1000 or GP681) OR AB (Viral Polymerase Complex Inhibitor* OR Favipiravir or T-705 or Avigan or FabiFlu or Pimodivir or VX-787 or JNJ-63623872 or AL-794 or ALS-033719 or ZSP1273 or Enisamium iodide or FAV00A or TG-1000 or GP681)	Search modes - Boolean/Phrase	179
S31	TI (oseltamivir or tamiflu or "GS 4104" or GS4104 or GS-4104 or "GS 4071" or GS4071 or GS-4071 or zanamivir or relenza or "GG 167" or GG167 or GG-167 or CS-8958 or Dectova or Laninamivir or R-125489 or R125489 or "R 125489" or Inavir or peramivir or "BCX 1812" or BCX1812 or BCX-1812 or "RWJ 270201" or RWJ270201 or RWJ-270201 or Rapivab or rapiacta) OR AB (oseltamivir or tamiflu or "GS 4104" or GS4104 or GS-4104 or "GS 4071" or GS4071 or GS-4071 or zanamivir or relenza or "GG 167" or GG167 or GG-167 or CS-8958 or Dectova or Laninamivir or R-125489 or R125489 or "R 125489" or Inavir or peramivir or "BCX 1812" or BCX1812 or BCX-1812 or "RWJ 270201" or RWJ270201 or RWJ-270201 or Rapivab or rapiacta)	Search modes - Boolean/Phrase	1,256
S30	(MH "Oseltamivir")	Search modes - Boolean/Phrase	787

S29	TI(neuraminidase inhibitor* or NA inhibitor*) OR AB(neuraminidase inhibitor* or NA inhibitor*)	Search modes - Boolean/Phrase	397
S28	TI Antiviral* OR AB Antiviral*	Search modes - Boolean/Phrase	11,069
S27	(MH "Antiviral Agents")	Search modes - Boolean/Phrase	19,889
S26	S24 OR S25	Search modes - Boolean/Phrase	33,506
S25	TI (Influenza or flu or H1N1 or PH1N1 or H3N2 or AH1N1 or AH3N2 or H5N1 or H7N9) OR AB (Influenza or flu or H1N1 or PH1N1 or H3N2 or AH1N1 or AH3N2 or H5N1 or H7N9)	Search modes - Boolean/Phrase	29,072
S24	(MH "Influenza+") OR (MH "Influenza A Virus+") OR (MH "Influenzavirus C") OR (MH "Influenza B Virus")	Search modes - Boolean/Phrase	22,039
S23	S22 NOT S21	Search modes - Boolean/Phrase	971,896
S22	S1 OR S2 OR S3 OR S4 OR S5 OR S6 OR S7 OR S8 OR S9 OR S10 OR S11 OR S12 OR S13 OR S14 OR S15	Search modes - Boolean/Phrase	1,019,541
S21	S19 NOT S20	Search modes - Boolean/Phrase	213,830
S20	MH (human)	Search modes - Boolean/Phrase	2,696,267
S19	S16 OR S17 OR S18	Search modes - Boolean/Phrase	247,788
S18	TI (animal model*)	Search modes - Boolean/Phrase	3,563
S17	MH (animal studies)	Search modes - Boolean/Phrase	153,253
S16	MH animals+	Search modes - Boolean/Phrase	103,495
S15	AB (cluster W3 RCT)	Search modes - Boolean/Phrase	498
S14	MH (crossover design) OR MH (comparative studies)	Search modes - Boolean/Phrase	478,421

S13	AB (control W5 group)	Search modes - Boolean/Phrase	145,440
S12	PT (randomized controlled trial)	Search modes - Boolean/Phrase	151,971
S11	MH (placebos)	Search modes - Boolean/Phrase	13,800
S10	MH (sample size) AND AB (assigned OR allocated OR control)	Search modes - Boolean/Phrase	4,424
S9	TI (trial)	Search modes - Boolean/Phrase	183,598
S8	AB (random*)	Search modes - Boolean/Phrase	400,359
S7	TI (randomised OR randomized)	Search modes - Boolean/Phrase	142,994
S6	MH cluster sample	Search modes - Boolean/Phrase	5,250
S5	MH pretest-posttest design	Search modes - Boolean/Phrase	53,382
S4	MH random assignment	Search modes - Boolean/Phrase	80,504
S3	MH single-blind studies	Search modes - Boolean/Phrase	16,026
S2	MH double-blind studies	Search modes - Boolean/Phrase	Display
S1	MH randomized controlled trials	Search modes - Boolean/Phrase	Display

Appendix 2 Risk of bias tool

The modified Cochrane instrument evaluates eight domains, including random sequence generation; allocation concealment; blinding of participants, healthcare providers, data collectors and outcome assessors; incomplete outcome data (if the rate of missing data was less than 10%, we judged it as low risk of bias); and other potential sources of bias (e.g., early trial discontinuation). We rated each domain at the outcome level as either: definitely or probably low risk of bias (low risk of bias), probably or definitely high risk of bias (high risk of bias).

The Cochrane risk of bias instrument 2 evaluates six domains, including randomization, the timing of identification and recruitment of participants, intended interventions, incomplete outcome data, measurement of the outcome and selection of the reported result. We rated each domain at the outcome level as either: low risk of bias, unclear risk of bias and high risk of bias. When there were more than two domains with high risk of bias, we rated the outcome of that study as high risk of bias.

Appendix 3 Prior hypotheses and the anticipated direction of effects for subgroup analysis.

We planned to conduct subgroup analyses with hypothesized direction of effect as follows:

1. Influenza virus type (antivirals have greater effectiveness for prevention of pandemic influenza than for seasonal or zoonotic influenza);
2. Age (antivirals are more effective for prevention of influenza in non-older adults and adolescents than other age groups exposed to influenza such as young children aged <2 years, children aged 2-12 years, and older adults (≥65 years);
3. Exposure status (antivirals are more effective in exposed persons who utilized personal protective measures (e.g., facemasks) than in persons with unprotected exposures to influenza viruses;
4. Exposure to source of infection (antivirals are more effective for persons exposed to ill humans with influenza than for persons exposed to animal sources infected with influenza A viruses);
5. Influenza vaccination status (antivirals are more effective in vaccinated persons exposed to influenza viruses than in unvaccinated persons);
6. Disease severity (antivirals are more effective at reducing severe influenza in persons exposed to influenza viruses who are at high risk for severe disease, such as people who are pregnant or up to 2 weeks postpartum, extreme obesity (BMI≥40), or those have underlying health conditions (e.g., chronic respiratory, cardiovascular disease, immunocompromised) than in low risk persons)

When there were only between-trial data but ten or more studies were available, we performed random effects meta-regressions to examine the impact of continuous variables (e.g., age) on the effect of antivirals for preventing influenza.

Appendix 4 Methods for rating the certainty of evidence.

Following GRADE guidance, we rated the certainty of evidence for direct estimates of antiviral post-exposure prophylaxis as very low, low, moderate, or high by assessing limitations such as risk of bias, inconsistency, indirectness, and publication bias [31]. When outcome data were missing, we conducted a complete case analysis as the primary analysis. If the results of the primary analysis indicated a statistically significant effect of antiviral prophylaxis, we planned to evaluate the robustness by performing a plausible worst case sensitivity analysis for each outcome [32]. If the worst-case analyses significantly influenced the observed effect, we planned to rate down the certainty of evidence for risk of bias due to missing data.

To assess the evidence for indirect estimates, we focused on the dominant first order loop and rated the certainty of indirect evidence as the lowest certainty of the direct comparisons informing that dominant loop. When the distribution of plausible effect modification differed in the contributing direct comparisons, we further rated down indirect comparisons for intransitivity.

For the network analysis estimates, we started with the certainty of evidence that dominates the comparison (direct or indirect estimates). When direct and indirect estimates contributed similarly to the network estimate, we chose the higher of the two certainties. When incoherence existed between the direct and indirect estimates, we planned to rate down the certainty of evidence for incoherence and used the estimate with the higher certainty as the best estimate for effect.

We followed GRADE guidance in reporting results.[1] We developed the summary of finding tables using MAGICapp [https://app.magicapp.org], providing both relative and absolute effects and including plain language summaries with wording following GRADE guidance.[1,2]

Reference

1. Santesso N, Glenton C, Dahm P, et al. GRADE guidelines 26: informative statements to communicate the findings of systematic reviews of interventions. J Clin Epidemiol 2020; 119: 126-35.

2. Carrasco-Labra A, Brignardello-Petersen R, Santesso N, et al. Improving GRADE evidence tables part 1: a randomized trial shows improved understanding of content in summary of findings tables with a new format. J Clin Epidemiol 2016; 74: 7-18.

Appendix 5 Character population at high risk of severe disease.

The high-risk population included individuals with conditions such as chronic lung disease, cardiac disease, hematologic disorders, kidney or liver disorders, metabolic disease, immunosuppression, pregnant persons and those up to 2 weeks postpartum, children younger than 59 months, persons 65 years and older, people with a body mass index of 40 or higher, and those younger than 19 years of age who are on long-term aspirin- or salicylate-containing medications. All persons exposed to symptomatic humans or to animals infected with novel influenza A viruses known to be associated with high mortality in humans, or exposed to influenza A viruses circulating among animals with unknown disease severity in infected humans, were considered to be at high risk of severe disease.

Appendix 6 Basic characteristics of eligible studies

Study	Publication status Registration	Randomization design	Patients randomized	Country	Mean age, age range (years)	Male (%)	Exposure status	Vaccinated (%)	Treatment for patients	Treatment for index patients	Outcomes
Zanamivir vs Placebo (9 RCTs)											
Ambrozaitis 2005	Peer-reviewed publication NR	Individual RCT	489	Lithuania, Netherlands, Israel	67 20-107	52.6	Pre-exposure	9.2	Zanamivir 10mg inhalations vs placebo once daily *14 days	-	Laboratory-confirmed symptomatic influenza, laboratory-confirmed influenza, laboratory-confirmed asymptomatic infection, mortality, adverse events related to drugs, serious adverse events
Hayden 2000	Peer-reviewed publication NAI30010	Cluster RCT	837	USA	26.2 NR	44.9	Post-exposure	16.1	Zanamivir 10mg inhalations vs placebo once daily *10 days	Yes	Laboratory-confirmed symptomatic influenza, laboratory-confirmed influenza, laboratory-confirmed asymptomatic infection, adverse events related to drugs, serious adverse events
Kaiser 2000	Peer-reviewed publication NAIA/B2009	Individual RCT	575	Europe, North America	34.3 13-77	40.4	Post-exposure	0	Zanamivir 3.2mg intranasal vs 10mg inhalation vs 3.2mg intranasal and 10mg inhalation vs placebo once daily * 5 days	Unclear	Laboratory-confirmed symptomatic influenza, laboratory-confirmed influenza, laboratory-confirmed asymptomatic infection, adverse events related to drugs

LaForce 2007	Peer-reviewed publication NAI30034	Individual RCT	3363	Canada, the Czech Republic, France, Germany, Latvia, USA	60.4 12-94	42.1	Pre-exposure	67.2	Zanamivir 10 mg oral inhaled vs placebo once daily*28 days	-	Laboratory-confirmed symptomatic influenza, laboratory-confirmed influenza, laboratory-confirmed asymptomatic infection, mortality, adverse events related to drugs, serious adverse outcomes
Monto 1999	Peer-reviewed publication NAIA3005	Individual RCT	1107	USA	28.8 18-69	40.6	Pre-exposure	14.4	Zanamivir inhalation 10mg vs placebo once daily *28 days	-	Laboratory-confirmed symptomatic influenza, laboratory-confirmed influenza, laboratory-confirmed asymptomatic infection, adverse events related to drugs, serious adverse outcomes
Monto 2002	Peer-reviewed publication NAI30031	Cluster RCT	1291	North America, Europe, Australia, New Zealand, and South Africa	27.3 >=5	45.9	Post-exposure	10.2	Zanamivir inhalation 10 mg vs placebo once daily * 10 days	No	Laboratory-confirmed symptomatic influenza, laboratory-confirmed influenza, laboratory-confirmed asymptomatic infection
167-101	Trial registration	Individual RCT	317	Japan	33.6 >=18	26.5	Pre-exposure	0.3	Zanamivir inhalation 10 mg vs placebo once daily * 28 days	-	Laboratory-confirmed symptomatic influenza, laboratory-confirmed influenza, laboratory-confirmed asymptomatic infection, mortality, adverse events related to drugs, serious adverse outcomes

NAIA2006	Trial registration	Individual RCT	64	Canada, USA	28.7 >=13	56.7	Post-exposure	0	Zanamivir inhaled 5mg bid vs intranasal 6.4mg bid vs 10mg bid inhaled and 6.4mg bid intranasal placebo * 21 days	No	Laboratory-confirmed symptomatic influenza, laboratory-confirmed influenza, laboratory-confirmed asymptomatic infection, mortality, adverse events related to drugs, serious adverse outcomes
NAIB2006	Trial registration	Individual RCT	62	France, Sweden and the United Kingdom	35.45 >=18	54.9	Post-exposure	0	Zanamivir inhalation 10mg bid vs placebo * 5 days	No	Laboratory-confirmed symptomatic influenza, laboratory-confirmed influenza, laboratory-confirmed asymptomatic infection, mortality, adverse events related to drugs, serious adverse outcomes
Oseltamivir vs placebo (6 RCTs)											
Kashiwagi 2000	Peer-reviewed publication NR	Individual RCT	308	Japan	34.1 18-83	53.9	Pre-exposure	NR	Oseltamivir 75 mg orals vs placebo once daily * 42 days	-	Laboratory-confirmed symptomatic influenza, laboratory-confirmed influenza, laboratory-confirmed asymptomatic infection, serious adverse events
Welliver 2001	Peer-reviewed publication Wv15799	Cluster RCT	957	North America and Europe	33.4 12-85	49.0	Post-exposure	12.6	Oseltamivir 75mg vs placebo once daily * 7 days	No	laboratory-confirmed symptomatic influenza, laboratory-confirmed influenza, laboratory-confirmed asymptomatic infection, mortality, admission to hospital, adverse events related to drugs

Marianne 2014	Peer-reviewed publication NL27938.041.09	Cluster RCT	99	Netherlands	80.8 NR	34.3	Post-exposure	87.9	Oseltamivir 75 mg vs placebo once daily* 10 days	Yes	Laboratory-confirmed influenza, mortality
Hayden 1999	Peer-reviewed publication Wv15673 and Wv15697	Individual RCT	1559	USA	34.4 18-65	37.5	Pre-exposure	0	Oseltamivir 75 mg once daily vs 75 mg twice daily vs placebo * 42 days	-	Laboratory-confirmed symptomatic influenza, laboratory-confirmed influenza, laboratory-confirmed asymptomatic infection, mortality, admission to hospital, adverse events related to drugs
WV15708	Trial registration	Individual RCT	385	Australia, New Zealand, Saudi Arabia, Brazil	76.3 >= 65	41.1	Pre-exposure	80.3	Oseltamivir 75 mg vs placebo once daily * 42 days	-	Laboratory-confirmed symptomatic influenza, laboratory-confirmed influenza, laboratory-confirmed asymptomatic infection, mortality, admission to hospital, serious adverse events
WV15825	Trial registration	Individual RCT	548	USA, France, Netherlands, Belgium, United Kingdom	85.2 >= 65	31.0	Pre-exposure	97.3	Oseltamivir 75 mg vs placebo once daily * 42 days	-	Laboratory-confirmed symptomatic influenza, laboratory-confirmed influenza, laboratory-confirmed asymptomatic infection, mortality, admission to hospital, serious adverse events
Laninamivir vs placebo (3 RCTs)											

Kashiwagi 2013	Peer-reviewed publication JapicCTI-111647	Individual RCT	1711	Japan	34.1 >=10	12.3	Post-exposure	NR	Laninamivir inhalation 20mg once daily * 2 days vs 20mg once daily * 3 days vs placebo	Yes	Laboratory-confirmed symptomatic influenza, laboratory-confirmed influenza, laboratory-confirmed asymptomatic infection, adverse events related to antivirals
Kashiwagi 2016	Peer-reviewed publication JapicCTI-142679	Individual RCT	803	Japan	35.3 >=10	11.0	Post-exposure	37.8	Laninamivir inhalation 40mg once vs 20mg once daily * 2 days vs placebo	Yes	Laboratory-confirmed symptomatic influenza, laboratory-confirmed influenza, laboratory-confirmed asymptomatic infection, adverse events related to antivirals
Nakano 2016	Peer-reviewed publication NR	Individual RCT	343	Japan	6.8 <10	49.9	Post-exposure	41.1	Laninamivir inhalation 20 mg once vs placebo	Yes	Laboratory-confirmed symptomatic influenza, laboratory-confirmed influenza, laboratory-confirmed asymptomatic infection, mortality, adverse events related to antivirals, serious adverse events
Baloxavir vs placebo (1 RCT)											
Ikematsu 2020	Peer-reviewed publication JapicCTI-184180	Individual RCT	752	Japan	33.6 NR	21.6	Post-exposure	34.1	Baloxavir oral (weight < 10 kg: 1mg/kg; 10 to < 20 kg, 10mg; 20 to < 40 kg, 20mg; ≥ 40 kg 40mg) vs placebo once	Yes	Laboratory-confirmed symptomatic influenza, laboratory-confirmed influenza, laboratory-confirmed asymptomatic infection, mortality, adverse events related to antivirals, serious adverse outcomes
Rimantadine vs Placebo (6 RCTs)											

Brady 1990	Peer-reviewed publication NR	Individual RCT	228	USA	31.4 18-55	25.4	Pre-exposure	0	Rimantadine 100 mg vs placebo orals once daily *14 days	-	Laboratory-confirmed influenza
Bricaire 1990	Peer-reviewed publication NR	Cluster RCT	301	France	NR NR	48.7	Post-exposure	NR	Rimantadine (adults 200 mg/d, older 100 mg/d, child 5mg/kg/d) vs placebo orals daily for 10 days	Unclear	Laboratory-confirmed influenza
Carwford 1988	Peer-reviewed publication NR	Individual RCT	107	USA	NR NR	NR	Pre-exposure	0	Rimantadine 5 mg/kg/d (maximum dose, 150 mg/d for children under 10 years of age and 200 mg/d for children more than 10 years) once vs placebo	-	Laboratory-confirmed influenza
Reuman 1988	Peer-reviewed publication NR	Individual RCT	476	USA	NR 18-55	NR	Pre-exposure	0	Rimantadine 100mg/d vs 200mg/d vs placebo *42 days	-	Laboratory-confirmed influenza
Hayden 1989	Peer-reviewed publication NR	Cluster RCT	115	USA	25.5 1-57	48.7	Post-exposure	0	Rimantadine (the daily dose was 200 mg for adults and for children over nine or	Yes	Laboratory-confirmed influenza

									those who weighed more than 30 kg. The dose was 5 mg per kilogram of body weight up to a maximum of 150 mg per day for children less than 9 years of age or for those weighing less than 30kg) vs placebo once daily for 10 days.		
Monto 1995	Peer-reviewed publication NR	Individual RCT	328	USA	86.3 NR	16.2	Pre-exposure	93.3	Rimantadine 200 mg/day vs 100 mg/day vs placebo for 56 days	-	Laboratory-confirmed influenza, mortality
Amantadine vs placebo (4 RCTs)											
Oker-Blom 1970	Peer-reviewed publication NR	Individual RCT	391	Finland	22 NR	66.2	Pre-exposure	NR	Amantadine 100 mg vs placebo bid *30 days	-	Laboratory-confirmed influenza
Payler 1984	Peer-reviewed publication NR	Individual RCT	536	Britain	NR 18 – 24	NR	Pre-exposure	100	Amantadine 100 mg vs placebo once daily *14 days	-	Laboratory-confirmed influenza

Pettersson 1980	Peer-reviewed publication NR	Individual RCT	192	Finland	50.6 >=10	71.2	Pre-exposure	NR	Amantadine 100 mg bid * 21 days vs 100mg bid *35 days vs placebo	-	Laboratory-confirmed influenza, mortality
Nafta 1970	Peer-reviewed publication NR	Individual RCT	215	Romania	NR 3-50	NR	Pre-exposure	NR	Amantadine 100 mg vs placebo bid * 20 days	-	Laboratory-confirmed influenza, mortality, serious adverse events
Zanamivir vs Rimantadine; Zanamivir vs standard care (1 RCT)											
Schilling 1988	Peer-reviewed publication NR	Cluster RCT	140	USA	NR NR	NR	Pre-exposure	NR	1. Zanamivir 10 mg inhaled and 4.4 mg intransally bid vs rimantadine 100 mg *14 days; 2. Zanamivir 10 mg inhaled and 4.4 mg intransally bid vs standard of care * 14 days	-	Laboratory-confirmed influenza
Rimantadine vs Amantadine vs placebo (2 RCT)											
Dolin 1982	Peer-reviewed publication NR	Individual RCT	378	USA	25.6 18-45	NR	Pre-exposure	0	Rimantadine 100mg bid vs amantadine 100mg bid vs placebo * 42 days	-	Laboratory-confirmed influenza

Quarles 1981	Peer-reviewed publication NR	Individual RCT	308	USA	NR 18–24	NR	Pre-exposure	NR	Rimantadine 100mg bid vs amantadine 100mg bid vs placebo * 42 days	-	Laboratory-confirmed influenza
Rimantadine vs Zanamivir (1 RCT)											
Gravenstein 2005	Peer-reviewed publication NAIA3003	Individual RCT	469	USA	76.0 44-102	29.3	Pre-exposure	97.3	Rimantadine 100mg once daily vs zanamivir oral inhalations 10mg once daily *14 days	-	Laboratory-confirmed symptomatic influenza, mortality, adverse events related to antivirals, serious adverse outcomes

RCT: randomized controlled trial; NR = not report

Note: Of the included trials, 33 reported laboratory-confirmed influenza outcomes, among which 18 reported laboratory-confirmed symptomatic influenza and laboratory-confirmed asymptomatic influenza virus infection separately; one trial only reported laboratory-confirmed symptomatic influenza; 14 trials only reported laboratory-confirmed influenza without specifying symptom status. Seventeen trials reported all-cause mortality; 4 reported admission to hospital; 13 reported adverse events related to drugs; and 16 reported serious adverse events. No studies reported duration of symptoms, length of hospitalization, progression to invasive mechanical ventilation, length of mechanical ventilation, progression of disease severity, admission to ICU, or emergence of antiviral resistance.

Appendix 7 Risk of bias for eligible studies

2a for individual randomized trials

Study	Trial registration	Outcome	Sequence generation	Allocation concealment	Blinding					Lost to follow-up	Other bias
					Patients	Health care providers	Data collectors	Outcome assessors and/or adjudicators	Data analysts		
Ambrozaitis 2005	NAIA3004	Laboratory-confirmed symptomatic influenza	Low	Low	Low	Low	Low	Low	Low	Low	Low
		Laboratory-confirmed influenza	Low	Low	Low	Low	Low	Low	Low	Low	Low
		Laboratory-confirmed asymptomatic influenza	Low	Low	Low	Low	Low	Low	Low	Low	Low
		Mortality	Low	Low	Low	Low	Low	Low	Low	Low	Low
		Adverse events	Low	Low	Low	Low	Low	Low	High	Low	Low
		Serious adverse events	Low	Low	Low	Low	Low	Low	High	Low	Low
		Adverse events related to drugs	Low	Low	Low	Low	Low	Low	High	Low	Low
Brady 1990	NA	Laboratory-confirmed influenza	Low	Low	Low	Low	Low	Low	Low	Low	Low
		Adverse events	Low	Low	Low	Low	High	High	High	Low	Low
Carwford 1988	NA	Laboratory-confirmed influenza	High	High	Low	Low	Low	Low	Low	Low	Low
		Adverse events	High	High	Low	Low	High	High	High	Low	Low
Dolin 1982	NA	Laboratory-confirmed influenza	Low	Low	Low	Low	Low	Low	Low	Low	Low
Gravenstein 2005	NAIA3003	Laboratory-confirmed symptomatic influenza	Low	Low	Low	Low	Low	Low	Low	Low	Low
		Mortality	Low	Low	Low	Low	Low	Low	Low	Low	Low
		Adverse events	Low	Low	Low	Low	Low	Low	Low	Low	Low
		Serious adverse events	Low	Low	Low	Low	Low	Low	Low	Low	Low
		Adverse events related to drugs	Low	Low	Low	Low	Low	Low	Low	Low	Low
Hayden 1999	Wv15673 Wv15697	Laboratory-confirmed symptomatic influenza	Low	Low	Low	Low	Low	Low	Low	Low	Low

		Laboratory-confirmed influenza	Low	Low	Low	Low	Low	Low	Low	Low	Low
		Laboratory-confirmed asymptomatic influenza	Low	Low	Low	Low	Low	Low	Low	Low	Low
		Mortality	Low	Low	Low	Low	Low	Low	Low	Low	Low
		Admission to hospital	Low	Low	Low	Low	Low	Low	Low	Low	Low
		Adverse events	Low	Low	Low	Low	High	High	High	Low	Low
		Serious adverse events	Low	Low	Low	Low	High	High	High	Low	Low
Ikematsu 2020	JapicCTI-184180	Laboratory-confirmed symptomatic influenza	Low	Low	Low	Low	Low	Low	Low	Low	Low
		Laboratory-confirmed influenza	Low	Low	Low	Low	Low	Low	Low	Low	Low
		Laboratory-confirmed asymptomatic influenza	Low	Low	Low	Low	Low	Low	Low	Low	Low
		Mortality	Low	Low	Low	Low	Low	Low	Low	Low	Low
		Adverse events	Low	Low	Low	Low	High	High	High	Low	Low
		Serious adverse events	Low	Low	Low	Low	High	High	High	Low	Low
		Adverse events related to drugs	Low	Low	Low	Low	High	High	High	Low	Low
Kaiser 2000	NAIA/B2009	Laboratory-confirmed symptomatic influenza	High	Low	Low	Low	Low	Low	Low	Low	Low
		Laboratory-confirmed influenza	High	Low	Low	Low	Low	Low	Low	Low	Low
		Laboratory-confirmed asymptomatic influenza	High	Low	Low	Low	Low	Low	Low	Low	Low
		Adverse events	High	Low	Low	Low	Low	Low	Low	Low	Low
		Adverse events related to drugs	High	Low	Low	Low	Low	Low	Low	Low	Low
Kashiwagi 2000	NA	Laboratory-confirmed symptomatic influenza	Low	Low	Low	Low	Low	Low	Low	Low	Low
		Laboratory-confirmed influenza	Low	Low	Low	Low	Low	Low	Low	Low	Low
		Laboratory-confirmed asymptomatic influenza	Low	Low	Low	Low	Low	Low	Low	Low	Low
		Adverse events	Low	Low	Low	Low	High	High	High	Low	Low
		Serious adverse events	Low	Low	Low	Low	High	High	High	Low	Low

Kashiwagi 2013	JapicCTI-111647	Laboratory-confirmed symptomatic influenza	Low	Low	Low	Low	Low	Low	Low	Low	Low
		Laboratory-confirmed influenza	Low	Low	Low	Low	Low	Low	Low	Low	Low
		Laboratory-confirmed asymptomatic influenza	Low	Low	Low	Low	Low	Low	Low	Low	Low
		Adverse events	Low	Low	Low	Low	Low	Low	Low	Low	Low
		Serious adverse events	Low	Low	Low	Low	Low	Low	Low	Low	Low
Kashiwagi 2016	JapicCTI-142679	Laboratory-confirmed symptomatic influenza	Low	Low	Low	Low	Low	Low	Low	Low	Low
		Laboratory-confirmed influenza	Low	Low	Low	Low	Low	Low	Low	Low	Low
		Laboratory-confirmed asymptomatic influenza	Low	Low	Low	Low	Low	Low	Low	Low	Low
		Adverse events	Low	Low	Low	Low	Low	Low	Low	Low	Low
		Serious adverse events	Low	Low	Low	Low	Low	Low	Low	Low	Low
LaForce 2007	NAI30034	Laboratory-confirmed symptomatic influenza	High	Low	Low	Low	Low	Low	Low	Low	Low
		Laboratory-confirmed influenza	High	Low	Low	Low	Low	Low	Low	Low	Low
		Laboratory-confirmed asymptomatic influenza	High	Low	Low	Low	Low	Low	Low	Low	Low
		Mortality	High	Low	Low	Low	Low	Low	Low	Low	Low
		Adverse events	High	Low	Low	Low	Low	Low	High	Low	Low
		Serious adverse events	High	Low	Low	Low	Low	Low	High	Low	Low
		Adverse events related to drugs	High	Low	Low	Low	Low	Low	High	Low	Low
Monto 1995	NA	Laboratory-confirmed influenza	High	High	Low	Low	Low	Low	Low	Low	Low
		Mortality	High	High	Low	Low	Low	Low	Low	Low	Low
Monto 1999	NAIA3005	Laboratory-confirmed influenza	Low	Low	Low	Low	Low	Low	Low	Low	Low
		Laboratory-confirmed influenza without symptoms	Low	Low	Low	Low	Low	Low	Low	Low	Low
		Laboratory-confirmed influenza with symptoms	Low	Low	Low	Low	Low	Low	Low	Low	Low

		Adverse events	Low	Low	Low	Low	High	High	High	Low	Low
		Serious adverse events	Low	Low	Low	Low	High	High	High	Low	Low
		Adverse events related to drugs	Low	Low	Low	Low	High	High	High	Low	Low
Nafta 1970	NA	Laboratory-confirmed influenza	High	High	Low	Low	Low	Low	Low	Low	Low
		Serious adverse events	High	High	Low	Low	High	High	High	Low	Low
Nakano 2016	NA	Laboratory-confirmed symptomatic influenza	Low	Low	Low	Low	Low	Low	Low	Low	Low
		Laboratory-confirmed influenza	Low	Low	Low	Low	Low	Low	Low	Low	Low
		Laboratory-confirmed asymptomatic influenza	Low	Low	Low	Low	Low	Low	Low	Low	Low
		Mortality	Low	Low	Low	Low	Low	Low	Low	Low	Low
		Adverse events	Low	Low	Low	Low	High	High	High	Low	Low
		Serious adverse events	Low	Low	Low	Low	High	High	High	Low	Low
		Adverse events related to drugs	Low	Low	Low	Low	High	High	High	Low	Low
Oker-Blom 1970	NA	Laboratory-confirmed influenza	High	Low	Low	Low	Low	Low	Low	Low	Low
Payler 1984	NA	Laboratory-confirmed influenza	High	High	Low	Low	Low	Low	Low	Low	Low
Pettersson 1980	NA	Laboratory-confirmed influenza	Low	Low	Low	Low	Low	Low	Low	Low	Low
		Mortality	Low	Low	Low	Low	Low	Low	Low	Low	Low
		Adverse events	Low	Low	Low	Low	High	High	High	Low	Low
Quarles 1981	NA	Laboratory-confirmed influenza	High	High	Low	Low	Low	Low	Low	Low	High
Reuman 1988	NA	Laboratory-confirmed influenza	Low	Low	Low	Low	Low	Low	Low	Low	Low
		Adverse events	Low	Low	Low	Low	High	High	High	Low	Low
167-101	167-101	Laboratory-confirmed symptomatic influenza	Low	Low	Low	Low	Low	Low	Low	Low	Low
		Laboratory-confirmed influenza	Low	Low	Low	Low	Low	Low	Low	Low	Low
		Laboratory-confirmed asymptomatic influenza	Low	Low	Low	Low	Low	Low	Low	Low	Low
		Mortality	Low	Low	Low	Low	Low	Low	Low	Low	Low

		Adverse events	Low	Low	Low	Low	Low	Low	Low	Low	Low
		Serious adverse events	Low	Low	Low	Low	Low	Low	Low	Low	Low
		Adverse events related to drugs	Low	Low	Low	Low	Low	Low	Low	Low	Low
NAIA2006	NAIA2006	Laboratory-confirmed symptomatic influenza	Low	Low	Low	Low	Low	Low	Low	High	Low
		Laboratory-confirmed influenza	Low	Low	Low	Low	Low	Low	Low	High	Low
		Laboratory-confirmed asymptomatic influenza	Low	Low	Low	Low	Low	Low	Low	High	Low
		Mortality	Low	Low	Low	Low	Low	Low	Low	High	Low
		Adverse events	Low	Low	Low	Low	Low	Low	Low	High	Low
		Serious adverse events	Low	Low	Low	Low	Low	Low	Low	High	Low
		Adverse events related to drugs	Low	Low	Low	Low	Low	Low	Low	High	Low
NAIB2006	NAIB2006	Laboratory-confirmed symptomatic influenza	Low	Low	Low	Low	Low	Low	Low	Low	Low
		Laboratory-confirmed influenza	Low	Low	Low	Low	Low	Low	Low	Low	Low
		Laboratory-confirmed asymptomatic influenza	Low	Low	Low	Low	Low	Low	Low	Low	Low
		Mortality	Low	Low	Low	Low	Low	Low	Low	Low	Low
		Adverse events	Low	Low	Low	Low	Low	Low	Low	Low	Low
		Serious adverse events	Low	Low	Low	Low	Low	Low	Low	Low	Low
		Adverse events related to drugs	Low	Low	Low	Low	Low	Low	Low	Low	Low
WV15708	WV15708	Laboratory-confirmed symptomatic influenza	Low	Low	Low	Low	Low	Low	Low	Low	Low
		Laboratory-confirmed influenza	Low	Low	Low	Low	Low	Low	Low	Low	Low
		Laboratory-confirmed asymptomatic influenza	Low	Low	Low	Low	Low	Low	Low	Low	Low
		Mortality	Low	Low	Low	Low	Low	Low	Low	Low	Low
		Admission to hospital	Low	Low	Low	Low	Low	Low	Low	Low	Low
		Adverse events	Low	Low	Low	Low	Low	Low	Low	Low	Low
		Serious adverse events	Low	Low	Low	Low	Low	Low	Low	Low	Low

		Adverse events related to drugs	Low	Low	Low	Low	Low	Low	Low	Low	Low
WV15825	WV15825	Laboratory-confirmed symptomatic influenza	Low	Low	Low	Low	Low	Low	Low	Low	Low
		Laboratory-confirmed influenza	Low	Low	Low	Low	Low	Low	Low	Low	Low
		Laboratory-confirmed asymptomatic influenza	Low	Low	Low	Low	Low	Low	Low	Low	Low
		Mortality	Low	Low	Low	Low	Low	Low	Low	Low	Low
		Admission to hospital	Low	Low	Low	Low	Low	Low	Low	Low	Low
		Adverse events	Low	Low	Low	Low	Low	Low	Low	Low	Low
		Serious adverse events	Low	Low	Low	Low	Low	Low	Low	Low	Low

2b Risk of bias for cluster randomized trials.

Study	Trial registration	Outcome	Bias arising from the randomization process	Bias arising from the timing of identification and recruitment of participants	Bias due to deviations from intended interventions	Bias due to missing outcome data	Bias in measurement of the outcome	Bias in selection of the reported result
Bricaire 1990	NA	Laboratory-confirmed influenza	High	High	Low	Low	Low	Low
		Adverse events	High	High	Low	Low	Low	Low
Hayden 1989	NA	Laboratory-confirmed influenza	High	High	Low	Low	Low	Low
Hayden 2000	NAI30010	Laboratory-confirmed symptomatic influenza	High	Low	Low	Low	Low	Low
		Laboratory-confirmed influenza	High	Low	Low	Low	Low	Low
		Laboratory-confirmed asymptomatic influenza	High	Low	Low	Low	Low	Low
		Adverse events	High	Low	Low	Low	Low	Low
		Serious adverse events	High	Low	Low	Low	Low	Low

		Adverse events related to drugs	High	Low	Low	Low	Low	Low
Marianne 2014	NL27938.04 1.09	Laboratory-confirmed influenza	Low	Low	Low	Low	Low	Low
		Mortality	Low	Low	Low	Low	Low	Low
		Adverse events	Low	Low	Low	Low	Low	Low
Monto 2002	NAI30031	Laboratory-confirmed symptomatic influenza	High	High	Low	Low	Low	Low
		Laboratory-confirmed influenza	High	High	Low	Low	Low	Low
		Laboratory-confirmed asymptomatic influenza	High	High	Low	Low	Low	Low
		Adverse events	High	High	Low	Low	Low	Low
Schilling 1988	NA	Laboratory-confirmed influenza	High	High	Low	Low	Low	Low
Welliver 2001	Wv15799	Laboratory-confirmed symptomatic influenza	Low	Low	Low	Low	Low	Low
		Laboratory-confirmed influenza	Low	Low	Low	Low	Low	Low
		Laboratory-confirmed asymptomatic influenza	Low	Low	Low	Low	Low	Low
		Mortality	Low	Low	Low	Low	Low	Low
		Admission to hospital	Low	Low	Low	Low	Low	Low
		Adverse events	Low	Low	Low	Low	Low	Low
		Serious adverse events	Low	Low	Low	Low	Low	Low
		Adverse events related to drugs	Low	Low	Low	Low	Low	Low

Note: Among the included trials, five were rated at high risk of bias for the objective outcomes (laboratory-confirmed influenza virus infection, laboratory-confirmed symptomatic influenza, laboratory-confirmed asymptomatic influenza virus infection, admission to hospital, all-cause mortality) due to sequence generation and allocation concealment limitations, and eleven were rated at high risk of bias for subjective outcomes (adverse events related to drugs, serious adverse events), mostly due to failure to blind data collectors, outcome assessors and/or adjudicators

and data analysts. Among the cluster randomized trials, two were rated at high risk of bias for both objective outcomes and subjective outcomes [37, 56], and one was rated at high risk of bias for objective outcomes, all due to bias arising from the randomization process and bias arising from the timing of identification and recruitment of participants. Not applicable: NA.

Appendix 8 Network plots.

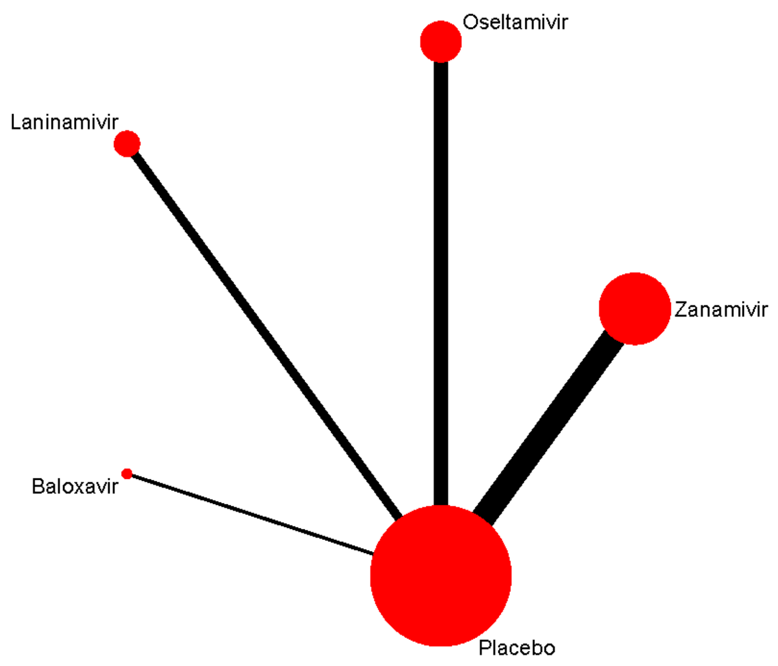

Figure 1 Network plot of trials evaluating antivirals prophylaxis against laboratory-confirmed asymptomatic influenza.

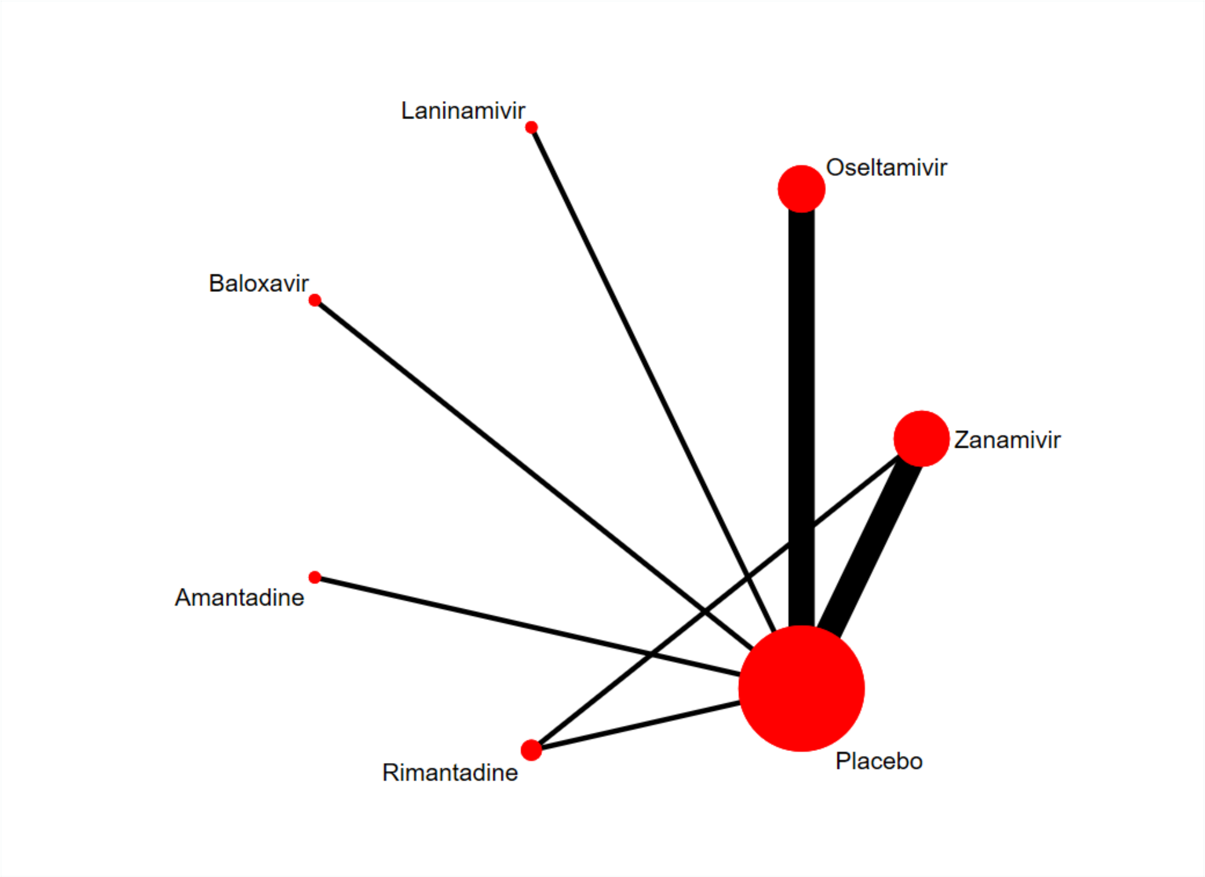

Figure 2 Network plot of trials evaluating antivirals prophylaxis against all-cause mortality.

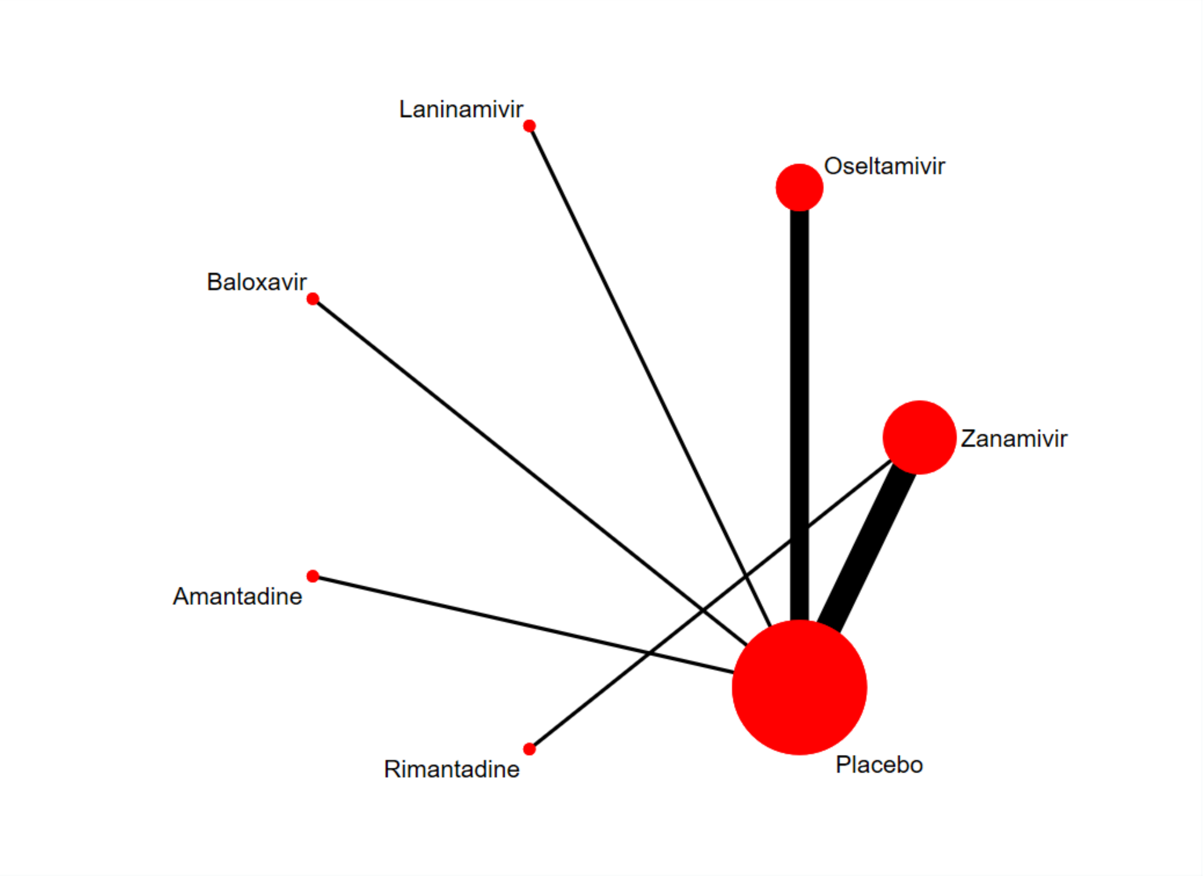

Figure 3 Network plot of trials evaluating antivirals prophylaxis against serious adverse events.

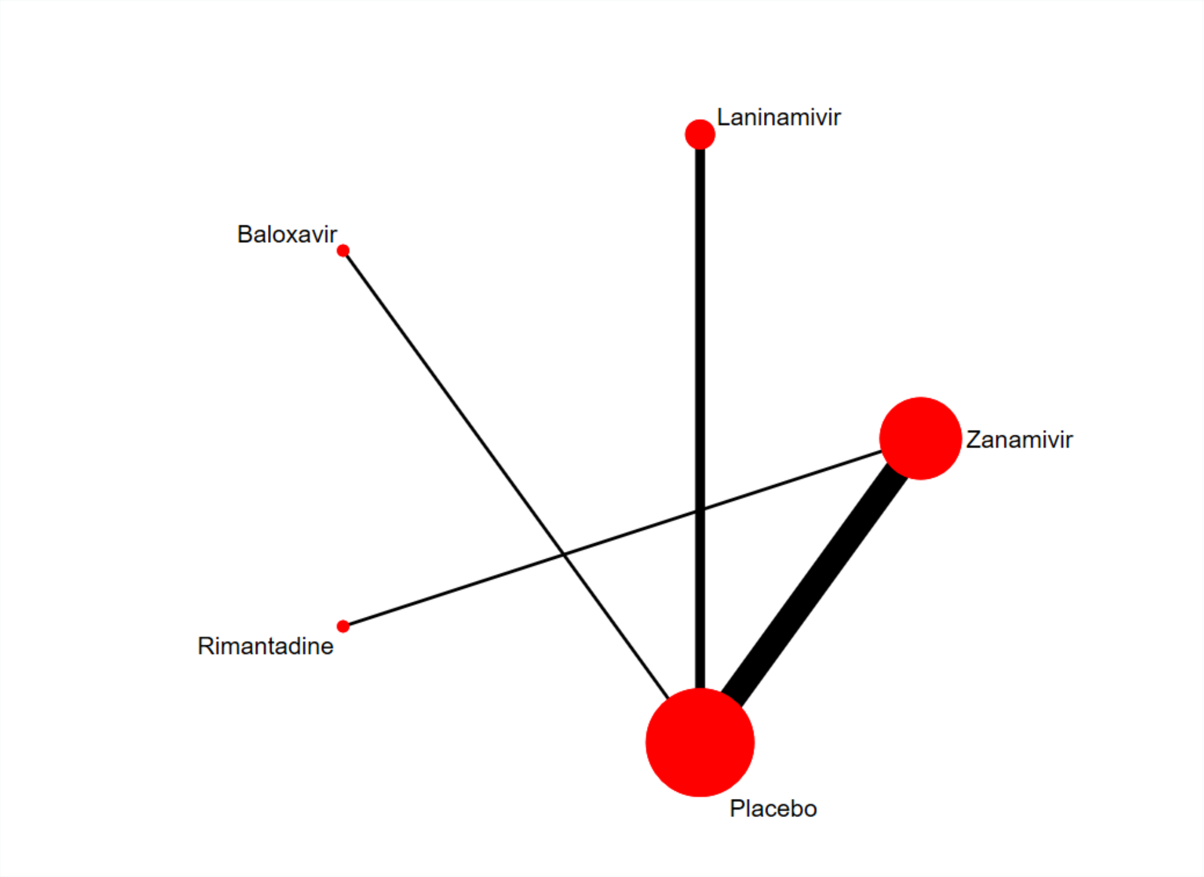

Figure 4 Network plot of trials evaluating antivirals prophylaxis against adverse events related to drugs.

Appendix 9 Forest plots

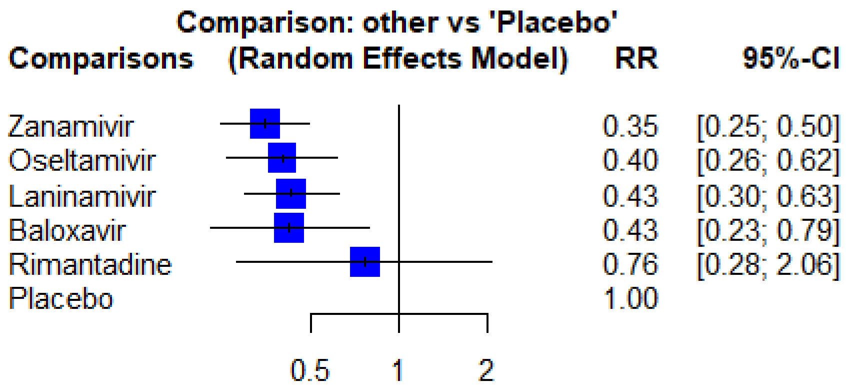

Figure 1 Forest plot for antivirals prophylaxis against laboratory-confirmed symptomatic influenza.

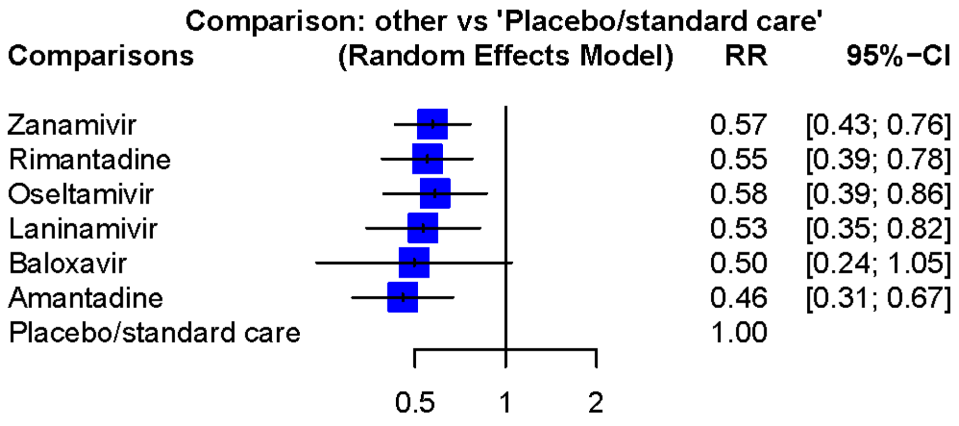

Figure 2 Forest plot for antivirals prophylaxis against laboratory-confirmed influenza.

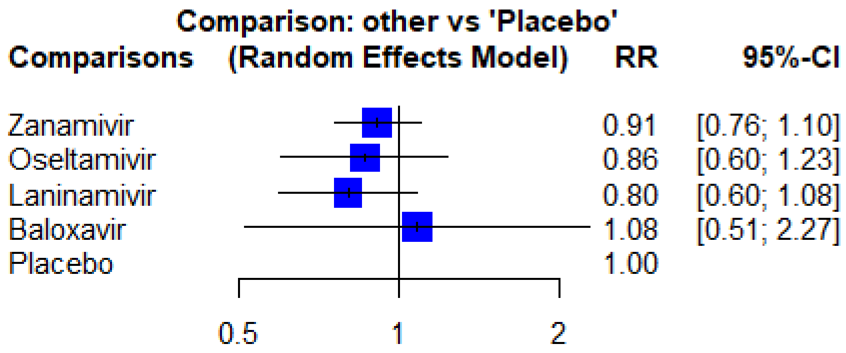

Figure 3 Forest plot for antivirals prophylaxis against laboratory-confirmed asymptomatic influenza.

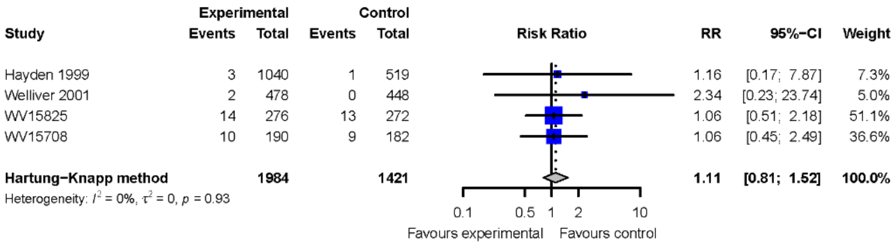

Figure 4 Forest plot for antivirals prophylaxis against admission to hospital.

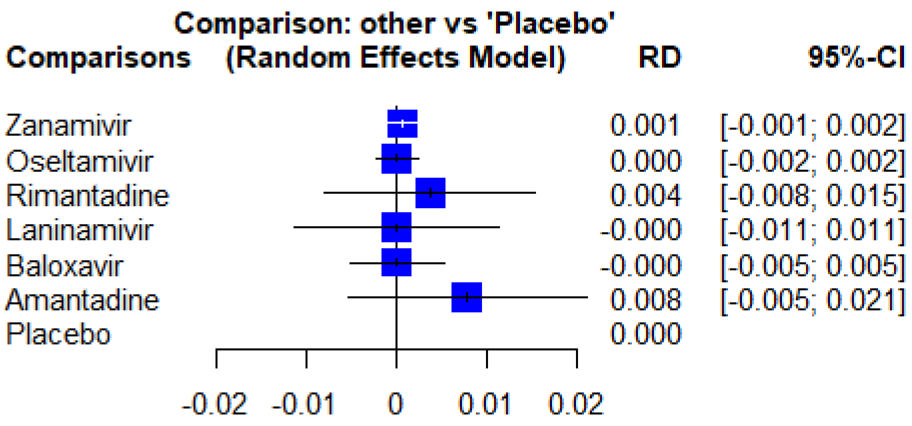

Figure 5 Forest plot for antivirals prophylaxis against all-cause mortality.

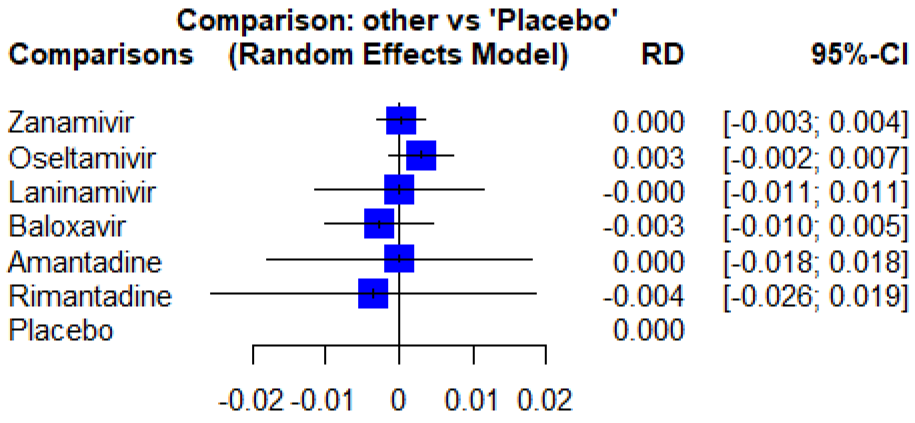

Figure 6 Forest plot for antivirals prophylaxis against serious adverse events.

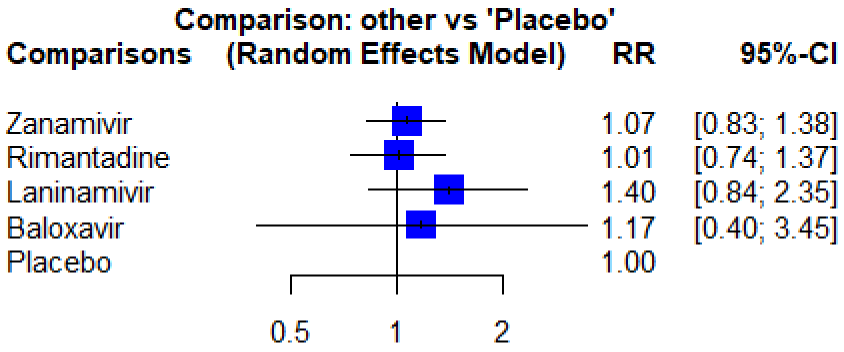

Figure 7 Forest plot for antivirals prophylaxis against adverse events related to drugs.

Appendix 10 Direct, indirect, and network treatment estimates for each outcome.

Table 1 Direct, indirect, and network treatment estimates for laboratory-confirmed symptomatic influenza.

Comparison	k	Prop	NMA (95% CI)	Direct (95% CI)	Indirect (95% CI)	Diff (95% CI)	z	Incoherence p-value
Baloxavir vs. Laninamivir	0	0	0.98 [0.48; 2.03]	NA	0.98 [0.48; 2.03]	NA	NA	NA
Baloxavir vs. Oseltamivir	0	0	1.06 [0.50; 2.26]	NA	1.06 [0.50; 2.26]	NA	NA	NA
Baloxavir vs. Placebo	1	1.00	0.43 [0.23; 0.79]	0.43 [0.23; 0.79]	NA	NA	NA	NA
Baloxavir vs. Rimantadine	0	0	0.56 [0.17; 1.80]	NA	0.56 [0.17; 1.80]	NA	NA	NA
Baloxavir vs. Zanamivir	0	0	1.21 [0.60; 2.47]	NA	1.21 [0.60; 2.47]	NA	NA	NA
Laninamivir vs. Oseltamivir	0	0	1.08 [0.61; 1.91]	NA	1.08 [0.61; 1.91]	NA	NA	NA
Laninamivir vs. Placebo	3	1.00	0.43 [0.30; 0.63]	0.43 [0.30; 0.63]	NA	NA	NA	NA
Laninamivir vs. Rimantadine	0	0	0.57 [0.20; 1.64]	NA	0.57 [0.20; 1.64]	NA	NA	NA
Laninamivir vs. Zanamivir	0	0	1.23 [0.74; 2.05]	NA	1.23 [0.74; 2.05]	NA	NA	NA
Oseltamivir vs. Placebo	5	1.00	0.40 [0.26; 0.62]	0.40 [0.26; 0.62]	NA	NA	NA	NA
Oseltamivir vs. Rimantadine	0	0	0.53 [0.18; 1.56]	NA	0.53 [0.18; 1.56]	NA	NA	NA
Oseltamivir vs. Zanamivir	0	0	1.14 [0.66; 1.99]	NA	1.14 [0.66; 1.99]	NA	NA	NA
Rimantadine vs. Placebo	0	0	0.76 [0.28; 2.06]	NA	0.76 [0.28; 2.06]	NA	NA	NA
Zanamivir vs. Placebo	9	1.00	0.35 [0.25; 0.50]	0.35 [0.25; 0.50]	NA	NA	NA	NA
Rimantadine vs. Zanamivir	1	1.00	2.17 [0.86; 5.53]	2.17 [0.86; 5.53]	NA	NA	NA	NA

Comparison: treatment comparison; k: number of studies providing direct evidence; prop: direct evidence proportion; NMA: estimated treatment effect (RR) in network meta-analysis; direct: estimated treatment effect (RR) derived from direct evidence; indirect: estimated treatment effect (RR) derived from indirect evidence;

RoR: Ratio of Ratios (direct versus indirect); z: z-value of test for disagreement (direct versus indirect): Incoherence p-value: p-value of test for disagreement (direct versus indirect)

Table 2 Direct, indirect, and network treatment estimates for laboratory-confirmed influenza.

**Comparison**	**k**	**Prop**	**NMA (95% CI)**	**Direct (95% CI)**	**Indirect (95% CI)**	**Diff (95% CI)**	**z**	**Incoherence p-value**
Amantadine vs. Baloxavir	0	0	0.92 [0.40; 2.12]	NA	0.92 [0.40; 2.12]	NA	NA	NA
Amantadine vs. Laninamivir	0	0	0.86 [0.48; 1.53]	NA	0.86 [0.48; 1.53]	NA	NA	NA
Amantadine vs. Oseltamivir	0	0	0.78 [0.45; 1.36]	NA	0.78 [0.45; 1.36]	NA	NA	NA
Amantadine vs. Placebo/stardard care	6	0.94	0.46 [0.31; 0.67]	0.45 [0.30; 0.67]	0.56 [0.12; 2.67]	0.81 [0.16; 4.10]	-0.25	0.8002
Amantadine vs. Rimantadine	2	0.50	0.83 [0.52; 1.33]	1.04 [0.54; 2.03]	0.66 [0.34; 1.28]	1.58 [0.62; 4.03]	0.96	0.3379
Amantadine vs. Zanamivir	0	0	0.80 [0.50; 1.28]	NA	0.80 [0.50; 1.28]	NA	NA	NA
Baloxavir vs. Laninamivir	0	0	0.93 [0.39; 2.21]	NA	0.93 [0.39; 2.21]	NA	NA	NA
Baloxavir vs. Oseltamivir	0	0	0.85 [0.37; 1.98]	NA	0.85 [0.37; 1.98]	NA	NA	NA
Baloxavir vs. Placebo/stardard care	1	1.00	0.50 [0.24; 1.05]	0.50 [0.24; 1.05]	NA	NA	NA	NA
Baloxavir vs. Rimantadine	0	0	0.90 [0.40; 2.06]	NA	0.90 [0.40; 2.06]	NA	NA	NA
Baloxavir vs. Zanamivir	0	0	0.87 [0.39; 1.93]	NA	0.87 [0.39; 1.93]	NA	NA	NA
Laninamivir vs. Oseltamivir	0	0	0.91 [0.51; 1.64]	NA	0.91 [0.51; 1.64]	NA	NA	NA
Laninamivir vs. Placebo/stardard care	3	1.00	0.53 [0.35; 0.82]	0.53 [0.35; 0.82]	NA	NA	NA	NA
Laninamivir vs. Rimantadine	0	0	0.97 [0.56; 1.68]	NA	0.97 [0.56; 1.68]	NA	NA	NA
Laninamivir vs. Zanamivir	0	0	0.93 [0.55; 1.56]	NA	0.93 [0.55; 1.56]	NA	NA	NA
Oseltamivir vs. Placebo/stardard care	6	1.00	0.58 [0.39; 0.86]	0.58 [0.39; 0.86]	NA	NA	NA	NA
Oseltamivir vs. Rimantadine	0	0	1.06 [0.63; 1.79]	NA	1.06 [0.63; 1.79]	NA	NA	NA
Oseltamivir vs. Zanamivir	0	0	1.02 [0.63; 1.66]	NA	1.02 [0.63; 1.66]	NA	NA	NA

Rimantadine vs. Placebo/stardard care	8	0.85	0.55 [0.39; 0.78]	0.52 [0.36; 0.75]	0.76 [0.32; 1.84]	0.68 [0.26; 1.76]	-0.80	0.4249
Zanamivir vs. Placebo/stardard care	10	0.93	0.57 [0.43; 0.76]	0.62 [0.46; 0.84]	0.21 [0.07; 0.59]	3.00 [1.01; 8.97]	1.97	0.0489
Rimantadine vs. Zanamivir	2	0.19	0.96 [0.63; 1.47]	2.35 [0.88; 6.33]	0.78 [0.49; 1.26]	3.00 [1.01; 8.97]	1.97	0.0489

Comparison: treatment comparison; k: number of studies providing direct evidence; prop: direct evidence proportion; NMA: estimated treatment effect (RR) in network meta-analysis; direct: estimated treatment effect (RR) derived from direct evidence; indirect: estimated treatment effect (RR) derived from indirect evidence; RoR: Ratio of Ratios (direct versus indirect); z: z-value of test for disagreement (direct versus indirect): Incoherence p-value: p-value of test for disagreement (direct versus indirect).

Table 3 Direct, indirect, and network treatment estimates for laboratory-confirmed asymptomatic influenza.

**Comparison**	**k**	**Prop**	**NMA (95% CI)**	**Direct (95% CI)**	**Indirect (95% CI)**	**Diff (95% CI)**	**z**	**Incoherence p-value**
Baloxavir vs. Laninamivir	0	0	1.35 [0.61; 2.99]	NA	1.35 [0.61; 2.99]	NA	NA	NA
Baloxavir vs. Oseltamivir	0	0	1.25 [0.55; 2.86]	NA	1.25 [0.55; 2.86]	NA	NA	NA
Baloxavir vs. Placebo	1	1.00	1.08 [0.51; 2.27]	1.08 [0.51; 2.27]	NA	NA	NA	NA
Baloxavir vs. Zanamivir	0	0	1.18 [0.55; 2.54]	NA	1.18 [0.55; 2.54]	NA	NA	NA
Laninamivir vs. Oseltamivir	0	0	0.93 [0.58; 1.48]	NA	0.93 [0.58; 1.48]	NA	NA	NA
Laninamivir vs. Placebo	3	1.00	0.80 [0.60; 1.08]	0.80 [0.60; 1.08]	NA	NA	NA	NA
Laninamivir vs. Zanamivir	0	0	0.88 [0.62; 1.25]	NA	0.88 [0.62; 1.25]	NA	NA	NA
Oseltamivir vs. Placebo	5	1.00	0.86 [0.60; 1.23]	0.86 [0.60; 1.23]	NA	NA	NA	NA
Oseltamivir vs. Zanamivir	0	0	0.94 [0.63; 1.42]	NA	0.94 [0.63; 1.42]	NA	NA	NA
Zanamivir vs. Placebo	9	1.00	0.91 [0.76; 1.10]	0.91 [0.76; 1.10]	NA	NA	NA	NA

Comparison: treatment comparison; k: number of studies providing direct evidence; prop: direct evidence proportion; NMA: estimated treatment effect (RR) in network meta-analysis; direct: estimated treatment effect (RR) derived from direct evidence; indirect: estimated treatment effect (RR) derived from indirect evidence; RoR: Ratio of Ratios (direct versus indirect); z: z-value of test for disagreement (direct versus indirect): Incoherence p-value: p-value of test for disagreement (direct versus indirect).

Table 4 Direct, indirect, and network treatment estimates for mortality.

Comparison	k	Prop	NMA (95% CI)	Direct (95% CI)	Indirect (95% CI)	Diff (95% CI)	z	Incoherence p-value
Amantadine vs. Baloxavir	0	0	0.008 [-0.006; 0.022]	NA	0.008 [-0.006; 0.022]	NA	NA	NA
Amantadine vs. Laninamivir	0	0	0.008 [-0.010; 0.025]	NA	0.008 [-0.010; 0.025]	NA	NA	NA
Amantadine vs. Oseltamivir	0	0	0.008 [-0.006; 0.021]	NA	0.008 [-0.006; 0.021]	NA	NA	NA
Amantadine vs. Placebo	1	1.00	0.008 [-0.005; 0.021]	0.008 [-0.005; 0.021]	NA	NA	NA	NA
Amantadine vs. Rimantadine	0	0	0.004 [-0.013; 0.022]	NA	0.004 [-0.013; 0.022]	NA	NA	NA
Amantadine vs. Zanamivir	0	0	0.007 [-0.006; 0.020]	NA	0.007 [-0.006; 0.020]	NA	NA	NA
Baloxavir vs. Laninamivir	0	0	0.000 [-0.013; 0.013]	NA	0.000 [-0.013; 0.013]	NA	NA	NA
Baloxavir vs. Oseltamivir	0	0	-0.000 [-0.006; 0.006]	NA	-0.000 [-0.006; 0.006]	NA	NA	NA
Baloxavir vs. Placebo	1	1.00	-0.000 [-0.005; 0.005]	-0.000 [-0.005; 0.005]	NA	NA	NA	NA
Baloxavir vs. Rimantadine	0	0	-0.004 [-0.016; 0.009]	NA	-0.004 [-0.016; 0.009]	NA	NA	NA
Baloxavir vs. Zanamivir	0	0	-0.001 [-0.006; 0.005]	NA	-0.001 [-0.006; 0.005]	NA	NA	NA
Laninamivir vs. Oseltamivir	0	0	-0.000 [-0.012; 0.012]	NA	-0.000 [-0.012; 0.012]	NA	NA	NA
Laninamivir vs. Placebo	3	1.00	-0.000 [-0.011; 0.011]	-0.000 [-0.011; 0.011]	NA	NA	NA	NA
Laninamivir vs. Rimantadine	0	0	-0.004 [-0.020; 0.013]	NA	-0.004 [-0.020; 0.013]	NA	NA	NA
Laninamivir vs. Zanamivir	0	0	-0.001 [-0.012; 0.011]	NA	-0.001 [-0.012; 0.011]	NA	NA	NA
Oseltamivir vs. Placebo	5	1.00	0.000 [-0.002; 0.002]	0.000 [-0.002; 0.002]	NA	NA	NA	NA
Oseltamivir vs. Rimantadine	0	0	-0.004 [-0.016; 0.008]	NA	-0.004 [-0.016; 0.008]	NA	NA	NA
Oseltamivir vs. Zanamivir	0	0	-0.001 [-0.003; 0.002]	NA	-0.001 [-0.003; 0.002]	NA	NA	NA

Rimantadine vs. Placebo	1	0.04	0.004 [-0.008; 0.015]	0.069 [0.013; 0.126]	0.001 [-0.011; 0.013]	0.069 [0.011; 0.126]	2.34	0.0191
Zanamivir vs. Placebo	5	1	0.001 [-0.001; 0.002]	0.001 [-0.001; 0.002]	0.069 [0.012; 0.127]	-0.069 [-0.126; -0.011]	-2.34	0.0191
Rimantadine vs. Zanamivir	1	0.96	0.003 [-0.009; 0.015]	0.000 [-0.012; 0.012]	0.069 [0.013; 0.125]	-0.069 [-0.126; -0.011]	-2.34	0.0191

Comparison: treatment comparison; k: number of studies providing direct evidence; prop: direct evidence proportion; NMA: estimated treatment effect (RD) in network meta-analysis; direct: estimated treatment effect (RD) derived from direct evidence; indirect: estimated treatment effect (RD) derived from indirect evidence; Diff: difference between direct and indirect treatment estimates; z: z-value of test for disagreement (direct versus indirect): Incoherence p-value: p-value of test for disagreement (direct versus indirect).

Table 5 Direct, indirect, and network treatment estimates for adverse events related to drugs.

**Comparison**	**k**	**Prop**	**NMA (95% CI)**	**Direct (95% CI)**	**Indirect (95% CI)**	**Diff (95% CI)**	**z**	**Incoherence p-value**
Baloxavir vs. Laninamivir	0	0	0.83 [0.25; 2.76]	NA	0.83 [0.25; 2.76]	NA	NA	NA
Baloxavir vs. Placebo	1	1.00	1.17 [0.40; 3.45]	1.17 [0.40; 3.45]	NA	NA	NA	NA
Baloxavir vs. Rimantadine	0	0	1.16 [0.38; 3.57]	NA	1.16 [0.38; 3.57]	NA	NA	NA
Baloxavir vs. Zanamivir	0	0	1.10 [0.36; 3.33]	NA	1.10 [0.36; 3.33]	NA	NA	NA
Laninamivir vs. Placebo	2	1.00	1.40 [0.84; 2.35]	1.40 [0.84; 2.35]	NA	NA	NA	NA
Laninamivir vs. Rimantadine	0	0	1.39 [0.76; 2.54]	NA	1.39 [0.76; 2.54]	NA	NA	NA
Laninamivir vs. Zanamivir	0	0	1.32 [0.74; 2.34]	NA	1.32 [0.74; 2.34]	NA	NA	NA
Rimantadine vs. Placebo	0	0	1.01 [0.74; 1.37]	NA	1.01 [0.74; 1.37]	NA	NA	NA
Zanamivir vs. Placebo	7	1	1.07 [0.83; 1.38]	1.07 [0.83; 1.38]	NA	NA	NA	NA
Rimantadine vs. Zanamivir	1	1	0.95 [0.80; 1.12]	0.95 [0.80; 1.12]	NA	NA	NA	NA

Comparison: treatment comparison; k: number of studies providing direct evidence; prop: direct evidence proportion; NMA: estimated treatment effect (RR) in network meta-analysis; direct: estimated treatment effect (RR) derived from direct evidence; indirect: estimated treatment effect (RR) derived from indirect evidence; RoR: Ratio of Ratios (direct versus indirect); z: z-value of test for disagreement (direct versus indirect): Incoherence p-value: p-value of test for disagreement (direct versus indirect).

Table 6 Direct, indirect, and network treatment estimates for serious adverse events.

Comparison	k	Prop	NMA (95% CI)	Direct (95% CI)	Indirect (95% CI)	Diff (95% CI)	z	Incoherence p-value
Amantadine vs. Baloxavir	0	0	0.003 [-0.017; 0.022]	NA	0.003 [-0.017; 0.022]	NA	NA	NA
Amantadine vs. Laninamivir	0	0	-0.000 [-0.021; 0.021]	NA	-0.000 [-0.021; 0.021]	NA	NA	NA
Amantadine vs. Oseltamivir	0	0	-0.003 [-0.021; 0.016]	NA	-0.003 [-0.021; 0.016]	NA	NA	NA
Amantadine vs. Placebo	1	1.00	-0.000 [-0.018; 0.018]	-0.000 [-0.018; 0.018]	NA	NA	NA	NA
Amantadine vs. Rimantadine	0	0	0.004 [-0.025; 0.032]	NA	0.004 [-0.025; 0.032]	NA	NA	NA
Amantadine vs. Zanamivir	0	0	-0.000 [-0.019; 0.018]	NA	-0.000 [-0.019; 0.018]	NA	NA	NA
Baloxavir vs. Laninamivir	0	0	-0.003 [-0.016; 0.011]	NA	-0.003 [-0.016; 0.011]	NA	NA	NA
Baloxavir vs. Oseltamivir	0	0	-0.006 [-0.014; 0.003]	NA	-0.006 [-0.014; 0.003]	NA	NA	NA
Baloxavir vs. Placebo	1	1.00	-0.003 [-0.010; 0.005]	-0.003 [-0.010; 0.005]	NA	NA	NA	NA
Baloxavir vs. Rimantadine	0	0	0.001 [-0.022; 0.024]	NA	0.001 [-0.022; 0.024]	NA	NA	NA
Baloxavir vs. Zanamivir	0	0	-0.003 [-0.011; 0.005]	NA	-0.003 [-0.011; 0.005]	NA	NA	NA
Laninamivir vs. Oseltamivir	0	0	-0.003 [-0.015; 0.009]	NA	-0.003 [-0.015; 0.009]	NA	NA	NA
Laninamivir vs. Placebo	1	1.00	0.000 [-0.011; 0.011]	0.000 [-0.011; 0.011]	NA	NA	NA	NA
Laninamivir vs. Rimantadine	0	0	0.004 [-0.021; 0.029]	NA	0.004 [-0.021; 0.029]	NA	NA	NA
Laninamivir vs. Zanamivir	0	0	-0.000 [-0.012; 0.012]	NA	-0.000 [-0.012; 0.012]	NA	NA	NA
Oseltamivir vs. Placebo	5	1.00	0.003 [-0.002; 0.007]	0.003 [-0.002; 0.007]	NA	NA	NA	NA
Oseltamivir vs. Rimantadine	0	0	0.006 [-0.016; 0.029]	NA	0.006 [-0.016; 0.029]	NA	NA	NA
Oseltamivir vs. Zanamivir	0	0	0.003 [-0.003; 0.008]	NA	0.003 [-0.003; 0.008]	NA	NA	NA

Rimantadine vs. Placebo	0	0	-0.004 [-0.026; 0.019]	NA	-0.004 [-0.026; 0.019]	NA	NA	NA
Zanamivir vs. Placebo	5	1	0.000 [-0.003; 0.004]	0.000 [-0.003; 0.004]	NA	NA	NA	NA
Rimantadine vs. Zanamivir	1	1.00	-0.004 [-0.026; 0.018]	-0.004 [-0.026; 0.018]	NA	NA	NA	NA

Comparison: treatment comparison; k: number of studies providing direct evidence; prop: direct evidence proportion; NMA: estimated treatment effect (RD) in network meta-analysis; direct: estimated treatment effect (RD) derived from direct evidence; indirect: estimated treatment effect (RD) derived from indirect evidence; Diff: difference between direct and indirect treatment estimates; z: z-value of test for disagreement (direct versus indirect): Incoherence p-value: p-value of test for disagreement (direct versus indirect).

Appendix 11 Network estimates with GRADE ratings

Table 1 Network estimates with GRADE ratings for laboratory-confirmed symptomatic influenza (seasonal influenza)

**Comparison groups**		**Network relative estimate (RR)**			**Low-risk populations**				**High-risk populations**			
					**Network absolute estimate (per 1000)**			**GRADE rating**	**Network absolute estimate (per 1000)**			**GRADE rating**
**Treatment 1**	**Treatment 2**	**Point estimate**	**95% CI lower limit**	**95% CI upper limit**	**Point estimate**	**95% CI lower limit**	**95% CI upper limit**		**Point estimate**	**95% CI lower limit**	**95% CI upper limit**	
Baloxavir	Laninamivir	0.98	0.48	2.03	-2	-41	80	Moderate	-2	-41	80	Moderate
Baloxavir	Oseltamivir	1.06	0.50	2.26	5	-39	98	Moderate	5	-39	98	Moderate
Baloxavir	Placebo	0.42	0.23	0.79	-44	-60	-16	Moderate	-44	-60	-16	Moderate
Baloxavir	Rimantadine	0.56	0.17	1.80	-34	-65	62	Moderate	-34	-65	62	Moderate
Baloxavir	Zanamivir	1.21	0.60	2.47	16	-31	115	Moderate	16	-31	115	Moderate
Laninamivir	Oseltamivir	1.08	0.61	1.91	6	-30	71	Moderate	6	-30	71	Moderate
Laninamivir	Placebo	0.43	0.30	0.63	-44	-55	-29	Moderate	-44	-55	-29	Moderate
Laninamivir	Rimantadine	0.57	0.20	1.64	-34	-62	50	Moderate	-34	-62	50	Moderate
Laninamivir	Zanamivir	1.23	0.74	2.05	18	-20	82	Moderate	18	-20	82	Moderate
Oseltamivir	Placebo	0.40	0.26	0.62	-47	-58	-30	Moderate	-47	-58	-30	Moderate
Oseltamivir	Rimantadine	0.53	0.18	1.56	-37	-64	44	Moderate	-37	-64	44	Moderate
Oseltamivir	Zanamivir	1.14	0.66	1.99	11	-27	77	Moderate	11	-27	77	Moderate
Rimantadine	Placebo	0.76	0.28	2.06	-19	-56	83	Moderate	-19	-56	83	Moderate
Zanamivir	Placebo	0.35	0.25	0.50	-51	-58	-39	Moderate	-51	-58	-39	Moderate
Zanamivir	Rimantadine	2.17	0.86	5.53	91	-11	353	Moderate	91	-11	353	Moderate

NA, not applicable.

Table 2 Network estimates with GRADE ratings for laboratory-confirmed influenza (seasonal influenza)

**Comparison groups**		**Network relative estimate**			**Network absolute estimate (per 1000)**			**GRADE rating**
**Treatment 1**	**Treatment 2**	**Point estimate**	**95% CI lower limit**	**95% CI upper limit**	**Point estimate**	**95% CI lower limit**	**95% CI upper limit**	
Amantadine	Baloxavir	0.92	0.40	2.12	-14	-106	198	Moderate
Amantadine	Laninamivir	0.86	0.48	1.53	-80	-108	94	Moderate
Amantadine	Oseltamivir	0.78	0.45	1.36	-39	-97	64	Moderate
Amantadine	Placebo/standard care	0.46	0.31	0.67	-96	-122	-58	Moderate
Amantadine	Rimantadine	0.83	0.52	1.33	-30	-85	58	Moderate
Amantadine	Zanamivir	0.80	0.50	1.28	-35	-88	50	Moderate
Baloxavir	Laninamivir	0.93	0.39	2.21	-12	-108	214	Moderate
Baloxavir	Oseltamivir	0.85	0.37	1.98	-27	-112	173	Moderate
Baloxavir	Placebo/standard care	0.50	0.24	1.05	-88	-129	9	Moderate
Baloxavir	Rimantadine	0.90	0.40	2.06	-18	-106	188	Moderate
Baloxavir	Zanamivir	0.87	0.39	1.93	-23	-108	165	Moderate
Laninamivir	Oseltamivir	0.91	0.51	1.64	-16	-87	113	Moderate
Laninamivir	Placebo/standard care	0.53	0.35	0.82	-83	-115	-32	Moderate
Laninamivir	Rimantadine	0.97	0.56	1.68	-5	-78	120	Moderate
Laninamivir	Zanamivir	0.93	0.55	1.56	-12	-80	99	Moderate
Oseltamivir	Placebo/standard care	0.58	0.39	0.86	-74	-108	-25	Moderate
Oseltamivir	Rimantadine	1.06	0.63	1.79	11	-65	140	Moderate
Oseltamivir	Zanamivir	1.02	0.63	1.66	4	-65	117	Moderate
Rimantadine	Placebo/standard care	0.55	0.39	0.78	-80	-108	-39	Low
Zanamivir	Placebo/standard care	0.57	0.43	0.76	-74	-101	-42	Low
Zanamivir	Rimantadine	0.96	0.63	1.47	-7	-65	83	Moderate

Table 3 Network estimates with GRADE ratings for laboratory-confirmed asymptomatic influenza (seasonal influenza)

**Comparison groups**		**Network relative estimate**			**Network absolute estimate**			**GRADE rating**
**Treatment 1**	**Treatment 2**	**Point estimate**	**95% CI lower limit**	**95% CI upper limit**	**Point estimate**	**95% CI lower limit**	**95% CI upper limit**	
Baloxavir	Laninamivir	1.35	0.61	2.99	17	-19	98	Moderate
Baloxavir	Oseltamivir	1.25	0.55	2.86	12	-22	91	Moderate
Baloxavir	Placebo	1.08	0.51	2.27	4	-24	62	Moderate
Baloxavir	Zanamivir	1.18	0.55	2.54	9	-22	75	Moderate
Laninamivir	Oseltamivir	0.93	0.58	1.48	-3	-21	24	High
Laninamivir	Placebo	0.80	0.60	1.08	-10	-20	4	High
Laninamivir	Zanamivir	0.88	0.62	1.25	-6	-19	12	High
Oseltamivir	Placebo	0.86	0.60	1.23	-7	-20	11	High
Oseltamivir	Zanamivir	0.94	0.63	1.42	-3	-18	21	High
Zanamivir	Placebo	0.91	0.76	1.10	-4	-12	5	High

NA, not applicable.

Table 4 Network estimates with GRADE ratings for all-cause mortality (seasonal influenza)

**Comparison groups**		**Network relative estimate (RR)**			**Network absolute estimate (per 1000)**			**GRADE rating**
**Treatment 1**	**Treatment 2**	**Point estimate**	**95% CI lower limit**	**95% CI upper limit**	**Point estimate**	**95% CI lower limit**	**95% CI upper limit**	
Amantadine	Baloxavir	NA	NA	NA	8	-6	22	Moderate
Amantadine	Laninamivir	NA	NA	NA	8	-10	25	Moderate
Amantadine	Oseltamivir	NA	NA	NA	8	-6	21	Moderate
Amantadine	Placebo	NA	NA	NA	8	-5	21	**Very Low**
Amantadine	Rimantadine	NA	NA	NA	4	-13	22	Moderate
Amantadine	Zanamivir	NA	NA	NA	7	-6	20	Moderate
Baloxavir	Laninamivir	NA	NA	NA	0	-13	13	Moderate
Baloxavir	Oseltamivir	NA	NA	NA	0	-6	6	Moderate
Baloxavir	Placebo	NA	NA	NA	0	-5	5	Moderate
Baloxavir	Rimantadine	NA	NA	NA	-4	-16	9	Moderate
Baloxavir	Zanamivir	NA	NA	NA	-1	-6	5	Moderate
Laninamivir	Oseltamivir	NA	NA	NA	0	--12	12	Moderate
Laninamivir	Placebo	NA	NA	NA	0	-11	11	Moderate
Laninamivir	Rimantadine	NA	NA	NA	-4	-20	13	Moderate
Laninamivir	Zanamivir	NA	NA	NA	-1	-12	11	Moderate
Oseltamivir	Placebo	NA	NA	NA	0	-2	2	High
Oseltamivir	Rimantadine	NA	NA	NA	-4	-16	8	Moderate
Oseltamivir	Zanamivir	NA	NA	NA	-1	-3	2	Moderate
Rimantadine	Placebo	NA	NA	NA	4	-8	15	Very Low
Zanamivir	Placebo	NA	NA	NA	1	-1	2	**Moderate**
Zanamivir	Rimantadine	NA	NA	NA	3	-9	15	Very Low

NA, not applicable.

Table 5 Network estimates with GRADE ratings for adverse events related to drugs (seasonal influenza and zoonotic influenza)

**Comparison groups**		**Network relative estimate**			**Network absolute estimate**			**GRADE rating**
**Treatment 1**	**Treatment 2**	**Point estimate**	**95% CI lower limit**	**95% CI upper limit**	**Point estimate**	**95% CI lower limit**	**95% CI upper limit**	
Baloxavir	Laninamivir	0.83	0.25	2.76	-6	-27	63	Low
Baloxavir	Placebo	1.17	0.40	3.45	6	-22	88	Low
Baloxavir	Rimantadine	1.16	0.38	3.75	6	-22	99	Low
Baloxavir	Zanamivir	1.10	0.36	3.33	4	-23	84	Low
Laninamivir	Placebo	1.40	0.84	2.35	14	-6	49	Moderate
Laninamivir	Rimantadine	1.39	0.76	2.54	14	-9	55	Moderate
Laninamivir	Zanamivir	1.32	0.74	2.34	12	-9	48	Moderate
Rimantadine	Placebo	1.01	0.74	1.37	0	-9	13	Moderate
Zanamivir	Placebo	1.07	0.83	1.38	3	-6	14	Moderate
Rimantadine	Zanamivir	0.95	0.80	1.12	-2	-7	4	High

Table 6 Network estimates with GRADE ratings for serious adverse events (seasonal influenza and zoonotic influenza)

Comparison groups		Network relative estimate			Network absolute estimate (per 1000)			GRADE rating
Treatment 1	Treatment 2	Point estimate	95% CI lower limit	95% CI upper limit	Point estimate	95% CI lower limit	95% CI upper limit	
Amantadine	Baloxavir	NA	NA	NA	3	-17	22	Low
Amantadine	Laninamivir	NA	NA	NA	-0	-21	21	Low
Amantadine	Oseltamivir	NA	NA	NA	-3	-21	16	Low
Amantadine	Placebo	NA	NA	NA	-0	-18	18	Low
Amantadine	Rimantadine	NA	NA	NA	4	-25	32	Low
Amantadine	Zanamivir	NA	NA	NA	-0	-19	18	Low
Baloxavir	Laninamivir	NA	NA	NA	-3	-16	11	Low
Baloxavir	Oseltamivir	NA	NA	NA	-6	-14	3	Low
Baloxavir	Placebo	NA	NA	NA	-3	-10	5	Low
Baloxavir	Rimantadine	NA	NA	NA	1	-22	24	Low
Baloxavir	Zanamivir	NA	NA	NA	-3	-11	5	Low
Laninamivir	Oseltamivir	NA	NA	NA	-3	--15	9	Low
Laninamivir	Placebo	NA	NA	NA	0	-11	11	Low
Laninamivir	Rimantadine	NA	NA	NA	4	-21	29	Low
Laninamivir	Zanamivir	NA	NA	NA	-0	-12	12	Low
Oseltamivir	Placebo	NA	NA	NA	3	-2	7	Moderate
Oseltamivir	Rimantadine	NA	NA	NA	6	-16	29	Low
Oseltamivir	Zanamivir	NA	NA	NA	3	-3	8	Low
Rimantadine	Placebo	NA	NA	NA	-4	-26	19	Moderate
Zanamivir	Placebo	NA	NA	NA	0	-3	4	High
Zanamivir	Rimantadine	NA	NA	NA	-4	-26	18	Low

NA, not applicable.

Table 7 Network estimates with GRADE ratings for laboratory-confirmed symptomatic influenza (zoonotic influenza)

Comparison groups		Network relative estimate			Network absolute estimate (per 1000)			GRADE rating
Treatment 1	Treatment 2	Point estimate	95% CI lower limit	95% CI upper limit	Point estimate	95% CI lower limit	95% CI upper limit	
Baloxavir	Laninamivir	0.98	0.48	2.03	NA	NA	NA	NA
Baloxavir	Oseltamivir	1.06	0.50	2.26	NA	NA	NA	NA
Baloxavir	Placebo	0.42	0.23	0.79	-17	-23	-6	Low
Baloxavir	Rimantadine	0.56	0.17	1.80	NA	NA	NA	NA
Baloxavir	Zanamivir	1.21	0.60	2.47	NA	NA	NA	NA
Laninamivir	Oseltamivir	1.08	0.61	1.91	NA	NA	NA	NA
Laninamivir	Placebo	0.43	0.30	0.63	-17	-21	-11	Low
Laninamivir	Rimantadine	0.57	0.20	1.64	NA	NA	NA	NA
Laninamivir	Zanamivir	1.23	0.74	2.05	NA	NA	NA	NA
Oseltamivir	Placebo	0.40	0.26	0.62	-18	-22	-11	Low
Oseltamivir	Rimantadine	0.53	0.18	1.56	NA	NA	NA	NA
Oseltamivir	Zanamivir	1.14	0.66	1.99	NA	NA	NA	NA
Rimantadine	Placebo	0.76	0.28	2.06	-7	-22	32	Very Low
Zanamivir	Placebo	0.35	0.25	0.50	-19	-22	-15	Low
Zanamivir	Rimantadine	2.17	0.86	5.53	NA	NA	NA	NA

NA, not applicable. *Estimate was informed by direct evidence because of serious incoherence in these comparisons.

Table 8 Network estimates with GRADE ratings for admission to hospital (zoonotic influenza)

**Comparison groups**		**Network relative estimate**			**Network absolute estimate (per 1000)**			**GRADE rating**
**Treatment 1**	**Treatment 2**	**Point estimate**	**95% CI lower limit**	**95% CI upper limit**	**Point estimate**	**95% CI lower limit**	**95% CI upper limit**	
Baloxavir	Placebo	NA	NA	NA	-14	-18	-5	Very Low
Laninamivir	Placebo	NA	NA	NA	-14	-17	-9	Very Low
Oseltamivir	Placebo	NA	NA	NA	-15	-18	9	Very Low
Zanamivir	Placebo	NA	NA	NA	-15	-18	-12	Very Low

NA, not applicable.

Table 9 Network estimates with GRADE ratings for all-cause mortality (zoonotic influenza)

**Comparison groups**		**Network relative estimate**			**Network absolute estimate (per 1000)**			**GRADE rating**
**Treatment 1**	**Treatment 2**	**Point estimate**	**95% CI lower limit**	**95% CI upper limit**	**Point estimate**	**95% CI lower limit**	**95% CI upper limit**	
Baloxavir	Placebo	NA	NA	NA	-3	-4	-1	Very Low
Laninamivir	Placebo	NA	NA	NA	-3	-3	-2	Very Low
Oseltamivir	Placebo	NA	NA	NA	-3	-4	2	Very Low
Zanamivir	Placebo	NA	NA	NA	-3	-4	-2	Very Low

NA, not applicable.

Appendix 12 Summary of findings table for antivirals versus placebo/standard care or placebo.

Table 1 Summary of findings table for zanamivir versus placebo or standard care for prophylaxis against seasonal influenza

**Outcome**<br>Timeframe	**Study results and measurements**	**Absolute effect estimates**		**Certainty of the Evidence**<br>(Quality of evidence)	**Summary**
		Placebo/standard care	Zanamivir		
Laboratory-confirmed symptomatic influenza	Risk ratio: 0.35<br>(CI 95% 0.25 - 0.50)<br>Based on data from 8104 participants in 9 studies	78<br>per 1000	27<br>per 1000	**Moderate**<br>Due to serious imprecision	Low-risk populations and high-risk populations: compared to placebo, zanamivir probably decreases laboratory-confirmed symptomatic influenza.
		Difference: 51 fewer per 1000<br>(CI 95% 58 fewer - 39 fewer)			
Laboratory-confirmed influenza	Risk ratio: 0.57<br>(CI 95% 0.43 – 0.76)<br>Based on data from 8156 participants in 10 studies	177<br>per 1000	101<br>per 1000	**Low**<br>Due to serious incoherent and imprecision	Compared to placebo/standard of care, zanamivir may decrease laboratory-confirmed influenza.
		Difference: 74 fewer per 1000<br>(CI 95% 101 fewer - 42 fewer)			
Laboratory-confirmed asymptomatic influenza	Risk ratio: 0.91<br>(CI 95% 0.76 – 1.10)<br>Based on data from 8104 participants in 9 studies	49<br>per 1000	45<br>per 1000	**High**	Compared to placebo, zanamivir has little or no effect on laboratory-confirmed asymptomatic influenza.
		Difference: 4 fewer per 1000<br>(CI 95% 12 fewer – 5 more)			
Admission to hospital (No data from RCT)					Zanamivir has little or no effect on admission to hospital due to the baseline risk being lower than the minimal important difference.
All-cause mortality	Risk difference: 0.001<br>(CI 95% -0.001 - 0.002)<br>Based on data from 4295 participants in 5 studies	0.2<br>per 1000	1.2<br>per 1000	**Moderate**<br>Due to serious incoherent	Compared to placebo, zanamivir probably has little or no effect on all-cause mortality
		Difference: 1 more per 1000<br>(CI 95% 1 fewer - 2 more)			
	Risk ratio: 1.07	36	39	**Moderate**	

Adverse events related to drugs	(CI 95% 0.83 - 1.38) Based on data from 6814 participants in 8 studies	per 1000	per 1000	Due to serious imprecision	Zanamivir probably has little or no effect on the risk of adverse events related to drugs.
		Difference: 3 more per 1000 (CI 95% 6 fewer - 14 more)			
Serious adverse events	Risk difference: 0.0 (CI 95% -0.003 - 0.004) Based on data from 6239 participants in 7 studies	0.2 per 1000	0.2 per 1000	**High**	Zanamivir has little or no effect on the risk of serious adverse events
		Difference: 0 fewer per 1000 (CI 95% 3 fewer - 4 more)			

Table 2 Summary of findings table for oseltamivir versus placebo for prophylaxis against seasonal influenza

**Outcome** Timeframe	**Study results and measurements**	**Absolute effect estimates**		**Certainty of the Evidence** (Quality of evidence)	**Summary**
		Placebo	Oseltamivir		
Laboratory-confirmed symptomatic influenza	Risk ratio: 0.40 (CI 95% 0.26 - 0.62) Based on data from 3742 participants in 5 studies	78 per 1000	31 per 1000	**Moderate** Due to serious imprecision	Low-risk populations: compared to placebo, oseltamivir probably has little or no effect on laboratory-confirmed symptomatic influenza.
		Difference: 47 fewer per 1000 (CI 95% 58 fewer - 30 fewer)			High-risk populations: compared to placebo, oseltamivir probably decreases laboratory-confirmed symptomatic influenza.
Laboratory-confirmed influenza	Risk ratio: 0.58 (CI 95% 0.39- 0.86) Based on data from 3821 participants in 6 studies	177 per 1000	101 per 1000	**Moderate** Due to serious imprecision	Compared to placebo/standard of care, oseltamivir probably decreases laboratory-confirmed influenza.
		Difference: 74 fewer per 1000 (CI 95% 108 fewer - 25 fewer)			
Laboratory-confirmed asymptomatic influenza	Risk ratio: 0.86 (CI 95% 0.60 – 1.23) Based on data from 3742 participants in 5 studies	49 per 1000	42 per 1000	**High**	Compared to placebo, oseltamivir has little or no effect on laboratory-confirmed asymptomatic influenza.
		Difference: 7 fewer per 1000 (CI 95% 20 fewer – 11 more)			
Admission to hospital	Risk ratio: 1.11 (CI 95% 0.81 - 1.52) Based on data from 3434 participants in 4 studies	25 per 1000	28 per 1000	**Moderate** Due to serious imprecision	Oseltamivir probably has little or no effect on admission to hospital.
		Difference: 3 more per 1000 (CI 95% 8 fewer - 22 more)			
All-cause mortality	Risk difference: 0 (CI 95% -0.002 - 0.002) Based on data from 3507 participants in 5 studies	0.2 per 1000	0.2 per 1000	**High**	Oseltamivir has little or no impact on all-cause mortality.
		Difference: 0 fewer per 1000 (CI 95% 2 fewer - 2 more)			
Adverse events related to drugs					Whether oseltamivir increases adverse

(No data from RCT)					events related to drugs is very uncertain.
Serious adverse events	Risk difference: 0.0 (CI 95% -0.002 - 0.007) Based on data from 3742 participants in 5 studies	0.2 per 1000	3.2 per 1000	**Moderate** Due to serious imprecision	Oseltamivir probably has little or no effect on adverse events related to drugs
		Difference: 3.0 more (CI 95% 2.0 fewer - 7.0 more)			

Table 3 Summary of findings table for laninamivir versus placebo for prophylaxis against seasonal influenza

**Outcome** Timeframe	**Study results and measurements**	**Absolute effect estimates**		**Certainty of the Evidence** (Quality of evidence)	**Summary**
		Placebo	Laninamivir		
Laboratory-confirmed symptomatic influenza	Risk ratio: 0.43 (CI 95% 0.30 - 0.63) Based on data from 2593 participants in 3 studies	78 per 1000	34 per 1000	**Moderate** Due to serious imprecision	Low-risk populations: compared to placebo, laninamivir probably has little or no effect on laboratory-confirmed symptomatic influenza.
		Difference: 44 fewer per 1000 (CI 95% 55 fewer - 29 fewer)			High-risk populations: compared to placebo, laninamivir probably decreases laboratory-confirmed symptomatic influenza
Laboratory-confirmed influenza	Risk ratio: 0.53 (CI 95% 0.35 - 0.82) Based on data from 2593 participants in 3 studies	177 per 1000	94 per 1000	**Moderate** Due to serious imprecision	Compared to placebo/standard of care, laninamivir probably decreases laboratory-confirmed influenza.
		Difference: 83 fewer per 1000 (CI 95% 115 fewer - 32 fewer)			
Laboratory-confirmed asymptomatic influenza	Risk ratio: 0.80 (CI 95% 0.60 – 1.08) Based on data from 2593 participants in 3 studies	49 per 1000	39 per 1000	**High**	Compared to placebo, laninamivir has little or no effect on laboratory-confirmed asymptomatic influenza.
		Difference: 10 fewer per 1000 (CI 95% 20 fewer – 4 more)			
Admission to hospital (No data from RCT)					Laninamivir has little or no effect on admission to hospital due to the baseline risk being lower than the minimal important difference.
All-cause mortality	Risk difference: 0 (CI 95% -0.011 - 0.011) Based on data from 341 participants in 1 study	0.2 per 1000	0.2 per 1000	**Moderate** Due to serious imprecision	Laninamivir probably has little or no impact on all-cause mortality.
		Difference: 0.0 fewer per 1000 (CI 95% 11.0 fewer - 11.0 more)			

Adverse events related to drugs	Risk ratio: 1.4<br>(CI 95% 0.84 - 2.35)<br>Based on data from 2806 participants in 3 studies	36<br>per 1000	50<br>per 1000	**Moderate**<br>Due to serious imprecision	Laninamivir probably increases adverse events related to drugs
		Difference: 14 more per 1000<br>(CI 95% 6 fewer - 49 more)			
Serious adverse events	Risk difference: 0<br>(CI 95% -0.011 – 0.011)<br>Based on data from 341 participants in 1 study	0.2<br>per 1000	0.2<br>per 1000	**Low**<br>Due to serious risk of bias, Due to serious imprecision[1]	Laninamivir may have little or no effect on serious adverse events
		Difference: 0 fewer per 1000<br>(CI 95% 11 fewer - 11 more)			

Table 4 Summary of findings table for baloxavir versus placebo for prophylaxis against seasonal influenza

<table>
<tr><th rowspan="2">Outcome
Timeframe</th><th rowspan="2">Study results and measurements</th><th colspan="2">Absolute effect estimates</th><th rowspan="2">Certainty of the Evidence
(Quality of evidence)</th><th rowspan="2">Summary</th></tr>
<tr><th>Placebo</th><th>Baloxavir</th></tr>
<tr><td rowspan="2">Laboratory-confirmed symptomatic influenza</td><td rowspan="2">Risk ratio: 0.43
(CI 95% 0.23 - 0.79)
Based on data from 749 participants in 1 study</td><td>78
per 1000</td><td>34
per 1000</td><td rowspan="2">Moderate
Due to serious imprecision</td><td>Low-risk populations: compared to placebo, baloxavir probably has little or no effect on laboratory-confirmed symptomatic influenza.</td></tr>
<tr><td colspan="2">Difference: 44 fewer per 1000
(CI 95% 60 fewer - 16 fewer)</td><td>High-risk populations: compared to placebo, baloxavir probably decreases laboratory-confirmed symptomatic influenza.</td></tr>
<tr><td rowspan="2">Laboratory-confirmed influenza</td><td rowspan="2">Risk ratio: 0.50
(CI 95% 0.24 – 1.05)
Based on data from 749 participants in 1 study</td><td>177
per 1000</td><td>89
per 1000</td><td rowspan="2">Moderate
Due to serious imprecision</td><td rowspan="2">Compared to placebo/standard of care, baloxavir probably decreases laboratory-confirmed influenza.</td></tr>
<tr><td colspan="2">Difference: 88 fewer per 1000
(CI 95% 129 fewer - 9 more)</td></tr>
<tr><td rowspan="2">Laboratory-confirmed asymptomatic influenza</td><td rowspan="2">Risk ratio: 1.08
(CI 95% 0.51 – 2.27)
Based on data from 749 participants in 1 study</td><td>49
per 1000</td><td>53
per 1000</td><td rowspan="2">Moderate
Due to serious imprecision</td><td rowspan="2">Compared to placebo, baloxavir probably has little or no effect on laboratory-confirmed asymptomatic influenza.</td></tr>
<tr><td colspan="2">Difference: 4 more per 1000
(CI 95% 24 fewer - 62 more)</td></tr>
<tr><td rowspan="2">Admission to hospital (No data from RCT)</td><td rowspan="2"></td><td></td><td></td><td rowspan="2"></td><td rowspan="2">Baloxavir has little or no effect on admission to hospital due to the baseline risk being lower than the minimal important difference.</td></tr>
<tr><td colspan="2"></td></tr>
<tr><td rowspan="2">All-cause mortality</td><td rowspan="2">Risk difference: 0
(CI 95% -0.005 – 0.005)
Based on data from 749 participants in 1 study</td><td>0.2
per 1000</td><td>0.2
per 1000</td><td rowspan="2">Moderate
Due to serious imprecision</td><td rowspan="2">Baloxavir probably has little or no effect on all-cause mortality</td></tr>
<tr><td colspan="2">Difference: 0.0 fewer
(CI 95% 5.0 fewer - 5.0 more)</td></tr>
</table>

Adverse events related to drugs	Risk ratio: 1.17<br>(CI 95% 0.4 - 3.45)<br>Based on data from 749 participants in 1 study	36<br>per 1000	42<br>per 1000	**Low**<br>Due to serious risk of bias, Due to serious imprecision	Baloxavir may have little or no difference on adverse events related to drugs
		Difference: 6 more per 1000<br>(CI 95% 22 fewer - 88 more)			
Serious adverse events	Risk difference: 3<br>(CI 95% -0.010 - 0.005)<br>Based on data from 749 participants in 1 study	0.2<br>per 1000	3.2<br>per 1000	**Low**<br>Due to serious risk of bias, Due to serious imprecision	Compared to placebo, baloxavir may have little or no effect on serious adverse events
		Difference: 3.0 fewer<br>(CI 95% 10.0 fewer - 5.0 more)			

Table 5 Summary of findings table for amantadine versus placebo for prophylaxis against seasonal influenza

**Outcome** Timeframe	**Study results and measurements**	**Absolute effect estimates**		**Certainty of the Evidence** (Quality of evidence)	**Summary**
		Placebo	Amantadine		
Laboratory-confirmed symptomatic influenza					Not applicable
Laboratory-confirmed influenza	Risk ratio: 0.46 (CI 95% 0.31 - 0.67) Based on data from 1703 participants in 6 studies	177 per 1000	81 per 1000	**Moderate** Due to heterogeneity	Compared to placebo/standard care, amantadine probably decreases laboratory-confirmed influenza.
		Difference: 96 fewer per 1000 (CI 95% 122 fewer - 58 fewer)			
Laboratory-confirmed asymptomatic influenza					Not applicable
Admission to hospital (No data from RCT)					Not applicable
All-cause mortality	Risk difference: 0.008 (CI 95% -0.005 - 0.021) Based on data from 511 participants in 1 study	0.2 per 1000	8.2 per 1000	**Very low** Due to extremely serious imprecision	Whether amantadine increases all-cause mortality is very uncertain
		Difference: 8.0 more (CI 95% 5.0 fewer - 21 more)			
Adverse events related to drugs					Not applicable
Serious adverse events	Risk difference: 0 (CI 95% -0.018 -0.018) Based on data from 215 participants in 1 study	0.2 per 1000	3.2 per 1000	**Low** Due to serious risk of bias, Due to serious imprecision	Compared to placebo, amantadine may have little or no effect on serious adverse events
		Difference: 0.0 fewer (CI 95% 18.0 fewer - 18.0 more)			

Table 6 Summary of findings table for rimantadine versus placebo for prophylaxis against seasonal influenza

**Outcome** Timeframe	**Study results and measurements**	**Absolute effect estimates** Placebo	**Absolute effect estimates** Rimantadine	**Certainty of the Evidence** (Quality of evidence)	**Summary**
Laboratory-confirmed symptomatic influenza	Risk ratio: 0.76 (CI 95% 0.28 - 2.06) Indirect evidence	78 per 1000	59 per 1000	**Moderate** Due to serious imprecision	Low-risk and high-risk populations: compared to placebo, rimantadine probably has little or no effect on laboratory-confirmed symptomatic influenza.
		Difference: 19 fewer per 1000 (CI 95% 56 fewer - 83 more)			
Laboratory-confirmed influenza	Risk ratio: 0.55 (CI 95% 0.39 - 0.78) Based on data from 1763 participants in 8 studies	177 per 1000	97 per 1000	**Low** Due to serious imprecision and heterogeneity	Compared to placebo/standard of care, rimantadine may decrease laboratory-confirmed influenza.
		Difference: 80 fewer per 1000 (CI 95% 108 fewer - 39 fewer)			
Laboratory-confirmed asymptomatic influenza					Not applicable
Admission to hospital (No data from RCT)					Not applicable
All-cause mortality	Risk difference: 0.004 (CI 95% -0.008 – 0.015) Based on data from 196 participants in 1 study	0.2 per 1000	4.2 per 1000	**Very low** Due to serious incoherent and extremely serious imprecision	Whether rimantadine increases all-cause mortality is very uncertain.
		Difference: 4.0 more (CI 95% 8.0 fewer - 15 more)			
Adverse events related to drugs	Risk ratio: 1.05 (CI 95% 0.79 - 1.4) Indirect evidence	36 per 1000	38 per 1000	**Moderate** Due to serious imprecision	Compared to placebo, rimantadine probably has little or effect on adverse events related to drugs.
		Difference: 2 more per 1000 (CI 95% 8 fewer - 14 more)			
Serious adverse events	Risk difference: -0.004 (CI 95% -0.026 – 0.019) Indirect evidence	0.2 per 1000	0.2 per 1000	**Moderate** Due to serious imprecision	Compared to placebo, rimantadine probably has little or no effect on serious adverse events.
		Difference: 4 fewer per 1000 (CI 95% 26 fewer - 19 more)			

Table 7 Summary of findings table for zanamivir versus placebo for prophylaxis against zoonotic influenza

**Outcome** Timeframe	**Study results and measurements**	**Absolute effect estimates**		**Certainty of the Evidence** (Quality of evidence)	**Summary**
		Placebo/standard of care	Zanamivir		
Laboratory-confirmed symptomatic influenza	Risk ratio: 0.35 (CI 95% 0.25 - 0.5) Based on data from 8104 participants in 9 studies	30 per 1000	11 per 1000	**Low** Due to very serious indirectness	Zanamivir may reduce laboratory-confirmed symptomatic influenza
		Difference: 19 fewer per 1000 (CI 95% 22 fewer - 15 fewer)			
Admission to hospital (model)	Risk difference: -0.015 (CI 95% -0.018 – -0.012) Zoonotic influenza model	24 per 1000	9 per 1000	**Very low** Due to very serious indirectness, Due to serious imprecision	Whether zanamivir reduces admission to hospital is very uncertain.
		Difference: 15 fewer per 1000 (CI 95% 18 fewer - 12 fewer)			
All-cause mortality (model)	Risk difference: 0.003 (CI 95% -0.004 – -0.002) Zoonotic influenza model	5 per 1000	2 per 1000	**Very low** Due to very serious indirectness, Due to serious imprecision	Whether zanamivir reduces all-cause mortality is very uncertain.
		Difference: 3 fewer per 1000 (CI 95% 4 fewer - 2 fewer)			
Adverse events related to drugs	Risk ratio: 1.07 (CI 95% 0.83 - 1.38) Based on data from 6814 participants in 8 studies	36 per 1000	39 per 1000	**Moderate** Due to serious imprecision	Zanamivir probably has little or no effect on the risk of adverse events related to drugs.
		Difference: 3 more per 1000 (CI 95% 6 fewer - 14 more)			
Serious adverse events	Risk difference: 0 (CI 95% -0.003 - 0.004) Based on data from 6239 participants in 7 studies	0.2 per 1000	0.2 per 1000	**High**	Zanamivir has little or no effect on the risk of serious adverse events
		Difference: 0 fewer per 1000 (CI 95% 3 fewer - 4 more)			

Table 8 Summary of findings table for oseltamivir versus placebo for prophylaxis against zoonotic influenza

**Outcome**<br>Timeframe	**Study results and measurements**	**Absolute effect estimates**		**Certainty of the Evidence**<br>(Quality of evidence)	**Summary**
		Placebo	Oseltamivir		
Laboratory-confirmed symptomatic influenza	Risk ratio: 0.4<br>(CI 95% 0.26 - 0.62)<br>Based on data from 3742 participants in 5 studies	30<br>per 1000	12<br>per 1000	**Low**<br>Due to very serious indirectness	Oseltamivir may reduce laboratory-confirmed symptomatic influenza
		Difference: 18 fewer per 1000<br>(CI 95% 22 fewer - 11 fewer)			
Admission to hospital (model)	Risk difference: -0.015<br>(CI 95% -0.018 – 0.009)<br>Zoonotic influenza model	24<br>per 1000	9<br>per 1000	**Very low**<br>Due to very serious indirectness,<br>Due to serious imprecision.	Whether oseltamivir reduces admission to hospital is very uncertain.
		Difference: 15 fewer per 1000<br>(CI 95% 18 fewer - 9 more)			
All-cause mortality (model)	Risk difference: -0.003<br>(CI 95% -0.004 – 0.002)<br>Zoonotic influenza model	5<br>per 1000	2<br>per 1000	**Very low**<br>Due to very serious indirectness,<br>Due to serious imprecision	Whether oseltamivir reduces all-cause mortality mortality is very uncertain.
		Difference: 3 fewer per 1000<br>(CI 95% 4 fewer - 2 more)			
Adverse events related to drugs (No data from RCT)					Whether oseltamivir increases adverse events related to drugs is very uncertain.
Serious adverse events	Risk difference: 0.003<br>(CI 95% -0.002 - 0.007)<br>Based on data from 3742 participants in 5 studies	0.2<br>per 1000	0.2<br>per 1000	**Moderate**<br>Due to serious imprecision	Oseltamivir has little or no effect on the risk of adverse events related to drugs
		Difference: 3.0 more<br>(CI 95% 2.0 fewer - 7.0 more)			

Table 9 Summary of findings table for laninamivir versus placebo for prophylaxis against zoonotic influenza

**Outcome** Timeframe	**Study results and measurements**	**Absolute effect estimates**		**Certainty of the Evidence** (Quality of evidence)	**Summary**
		Placebo	Laninamivir		
Laboratory-confirmed symptomatic influenza	Risk ratio: 0.43 (CI 95% 0.3 - 0.63) Based on data from 2593 participants in 3 studies	30 per 1000	13 per 1000	**Low** Due to very serious indirectness	Laninamivir may reduce laboratory-confirmed symptomatic influenza
		Difference: 17 fewer per 1000 (CI 95% 21 fewer - 11 fewer)			
Admission to hospital (model)	Risk difference: -0.014 (CI 95% -0.017 – 0.009) Zoonotic influenza model	24 per 1000	10 per 1000	**Very low** Due to very serious indirectness, Due to serious imprecision	Whether laninamivir reduces admission to hospital is very uncertain.
		Difference: 14 fewer per 1000 (CI 95% 17 fewer - 9 fewer)			
All-cause mortality (model)	Risk difference: -0.003 (CI 95% -0.003 – 0.002) Zoonotic influenza model	5 per 1000	2 per 1000	**Very low** Due to serious imprecision, Due to very serious indirectness.	Whether laninamivir reduces all-cause mortality is very uncertain.
		Difference: 3 fewer per 1000 (CI 95% 3 fewer - 2 fewer)			
Adverse events related to drugs	Risk ratio: 1.4 (CI 95% 0.84 - 2.35) Based on data from 2806 participants in 3 studies	36 per 1000	50 per 1000	**Moderate** Due to serious imprecision	Laninamivir probably increases the incidence of adverse events related to drugs
		Difference: 14 more per 1000 (CI 95% 6 fewer - 49 more)			
Serious adverse events	Risk difference: 0 (CI 95% -0.011 – 0.011) Based on data from 341 participants in 1 study	0.2 per 1000	0.2 per 1000	**Low** Due to serious risk of bias, Due to serious imprecision[1]	Laninamivir may have little or no effect on the risk of serious adverse events
		Difference: 0 fewer per 1000 (CI 95% 11 fewer - 11 more)			

Table 10 Summary of findings table for baloxavir versus placebo for prophylaxis against zoonotic influenza

**Outcome** Timeframe	**Study results and measurements**	**Absolute effect estimates**		**Certainty of the Evidence** (Quality of evidence)	**Summary**
		Placebo	Baloxavir		
Laboratory-confirmed symptomatic influenza	Risk ratio: 0.43 (CI 95% 0.23 - 0.79) Based on data from 749 participants in 1 study	30 per 1000	13 per 1000	**Low** Due to very serious indirectness	Baloxavir may reduce laboratory-confirmed symptomatic influenza
		Difference: 17 fewer per 1000 (CI 95% 23 fewer - 6 fewer)			
Admission to hospital (model)	Risk difference: -0.014 (CI 95% -0.018 - -0.005) Zoonotic influenza model	24 per 1000	10 per 1000	**Very low** Due to very serious indirectness, Due to serious imprecision	Whether baloxavir reduces admission to hospital is very uncertain.
		Difference: 14 fewer per 1000 (CI 95% 18 fewer - 5 fewer)			
All-cause mortality (model)	Risk difference: -0.003 (CI 95% -0.004 - -0.001) Zoonotic influenza model	5 per 1000	2 per 1000	**Very low** Due to serious imprecision, Due to very serious indirectness	Whether baloxavir reduces all-cause mortality is very uncertain.
		Difference: 3 fewer per 1000 (CI 95% 4 fewer - 1 fewer)			
Adverse events related to drugs	Risk ratio: 1.17 (CI 95% 0.4 - 3.45) Based on data from 749 participants in 1 study	36 per 1000	42 per 1000	**Low** Due to serious risk of bias, Due to serious imprecision	Baloxavir may have little or no difference on the incidence of adverse events related to drugs
		Difference: 6 more per 1000 (CI 95% 22 fewer - 88 more)			
Serious adverse events	Risk difference: -0.003 (CI 95% -0.010 - 0.005) Based on data from 749 participants in 1 study	0.2 per 1000	0.2 per 1000	**Low** Due to serious risk of bias, Due to serious imprecision	Compared to placebo, baloxavir may have little or no effect on serious adverse events
		Difference: 3.0 fewer (CI 95% 10.0 fewer - 5.0 more)			

Table 11 Summary of findings table for amantadine versus placebo for prophylaxis against zoonotic influenza

**Outcome**<br>Timeframe	**Study results and measurements**	**Absolute effect estimates**		**Certainty of the Evidence**<br>(Quality of evidence)	**Summary**
		Placebo	Amantadine		
Laboratory-confirmed symptomatic influenza					Not applicable
Admission to hospital (model)					Not applicable
All-cause mortality (model)					Not applicable
Adverse events related to drugs					Not applicable
Serious adverse events	Risk difference: 0<br>(CI 95% -0.018 -0.018)<br>Based on data from 215 participants in 1 study	0.2<br>per 1000	3.2<br>per 1000	**Low**<br>Due to serious risk of bias, Due to serious imprecision	Compared to placebo, amantadine may have little or no effect on serious adverse events
		Difference: 0.0 fewer<br>(CI 95% 18.0 fewer - 18.0 more)			

Table 12 Summary of findings table for rimantadine versus placebo for prophylaxis against zoonotic influenza

**Outcome**<br>Timeframe	**Study results and measurements**	**Absolute effect estimates**		**Certainty of the Evidence**<br>(Quality of evidence)	**Summary**
		Placebo	Rimantadine		
Laboratory-confirmed symptomatic influenza	Risk ratio: 0.76<br>(CI 95% 0.28 - 2.06)<br>Indirect evidence	30<br>per 1000	23<br>per 1000	**Very low**<br>Due to very serious indirectness, Due to serious imprecision	Whether rimantadine reduces laboratory-confirmed symptomatic influenza is very uncertain
		Difference: 7 fewer per 1000<br>(CI 95% 22 fewer - 32 more)			
Admission to hospital (model)					Not applicable
All-cause mortality (model)					Not applicable
Adverse events related to drugs	Risk ratio: 1.05<br>(CI 95% 0.79 - 1.4)<br>Indirect evidence	36<br>per 1000	38<br>per 1000	**Moderate**<br>Due to serious imprecision	Compared to placebo, rimantadine probably has little or effect on adverse events related to drugs.
		Difference: 2 more per 1000<br>(CI 95% 8 fewer - 14 more)			
Serious adverse events	Risk difference: -0.004<br>(CI 95% -0.026 – 0.019)<br>Indirect evidence	0.2<br>per 1000	0.2<br>per 1000	**Moderate**<br>Due to serious imprecision	Compared to placebo, rimantadine probably has little or no effect on serious adverse events.
		Difference: 4 fewer per 1000<br>(CI 95% 26 fewer - 19 more)			

Appendix 13 Assessment of global inconsistency

Clinical outcome	P value
Laboratory-confirmed symptomatic influenza	NA
Laboratory-confirmed influenza	0.1130
Laboratory-confirmed asymptomatic influenza	NA
Admission to hospital	NA
All-cause mortality	0.019
Adverse events related to drugs	NA
Serious adverse events	NA

Appendix 14 Assessment of between-study heterogeneity.

Outcome	Comparison	No. study	I^2
Laboratory-confirmed symptomatic influenza	Zanamivir vs. Placebo/standard care	9	43%
	Oseltamivir vs. Placebo	5	0%
	Laninamivir vs. Placebo	3	66%
	Baloxavir vs. Placebo	1	NA
	Rimantadine vs. Placebo	0	NA
	Zanamivir vs. Rimantadine	1	NA
Laboratory-confirmed influenza	Zanamivir vs. Placebo/standard care	10	50%
	Oseltamivir vs. Placebo/standard care	6	0%
	Laninamivir vs. Placebo/standard care	3	72%
	Baloxavir vs. Placebo/standard care	1	NA
	Amantadine vs. Placebo/standard care	6	83%
	Rimantadine vs. Placebo/standard care	8	51%
	Zanamivir vs. Rimantadine	2	0%
	Rimantadine vs. Amantadine	2	0%
Laboratory-confirmed asymptomatic influenza	Zanamivir vs. Placebo	9	0%
	Oseltamivir vs. Placebo	5	0%
	Laninamivir vs. Placebo	3	0%
	Baloxavir vs. Placebo	1	NA
Admission to hospital	Oseltamivir vs. Placebo	4	0%
Mortality	Zanamivir vs. Placebo	5	0%
	Oseltamivir vs. Placebo	5	0%
	Laninamivir vs. Placebo	1	NA
	Baloxavir vs. Placebo	1	NA
	Amantadine vs. Placebo	1	NA
	Rimantadine vs. Placebo	1	NA
	Zanamivir vs. Rimantadine	1	NA
Adverse events related to treatments	Zanamivir vs. Placebo	8	0%
	Laninamivir vs. Placebo	3	0%
	Baloxavir vs. Placebo	1	NA
	Rimantadine vs. Placebo	0	NA
	Zanamivir vs. Rimantadine	1	NA
Serious adverse events	Zanamivir vs. Placebo	7	0%
	Oseltamivir vs. Placebo	5	0%
	Laninamivir vs. Placebo	1	NA

	Baloxavir vs. Placebo	1	NA
	Amantadine vs. Placebo	1	NA
	Rimantadine vs. Placebo	0	NA
	Zanamivir vs. Rimantadine	1	NA

NA, not applicable.

Appendix 15 Funnel plot

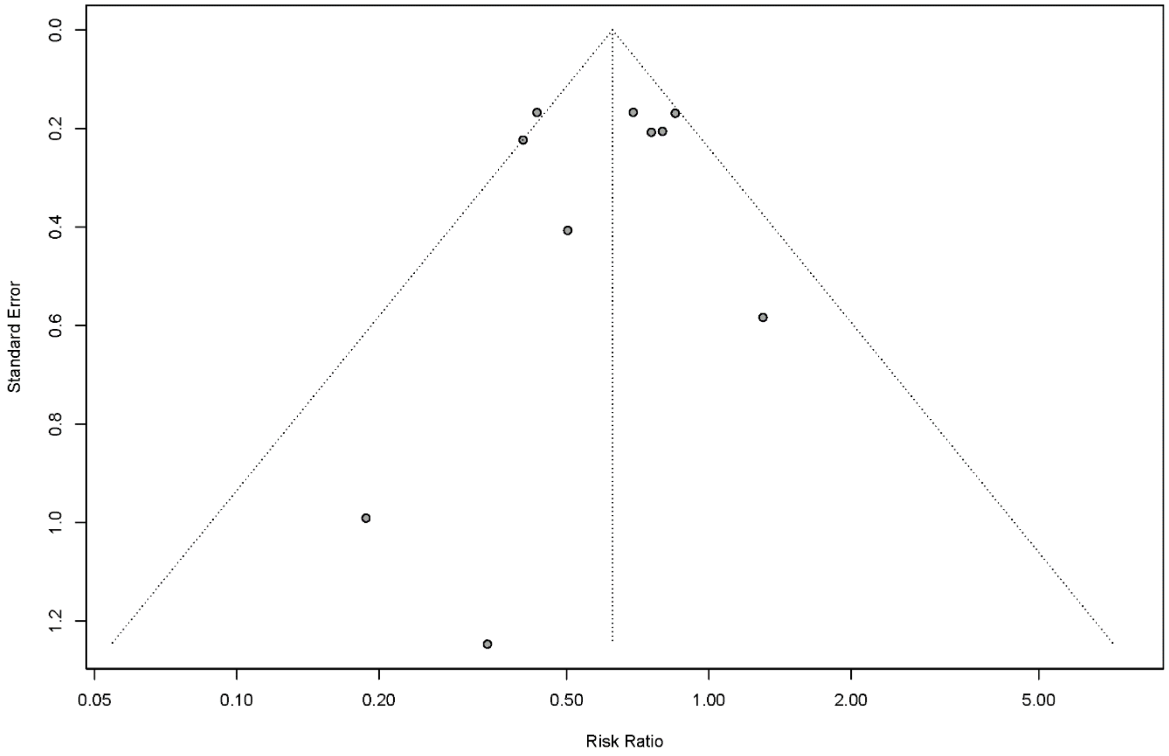

Figure 1 Funnel plot zanamivir vs placebo/ standard care for laboratory-confirmed influenza (Harbord test p = 0.77).

Appendix 16 Sensitivity analysis by only including the trials with lower risk of bias

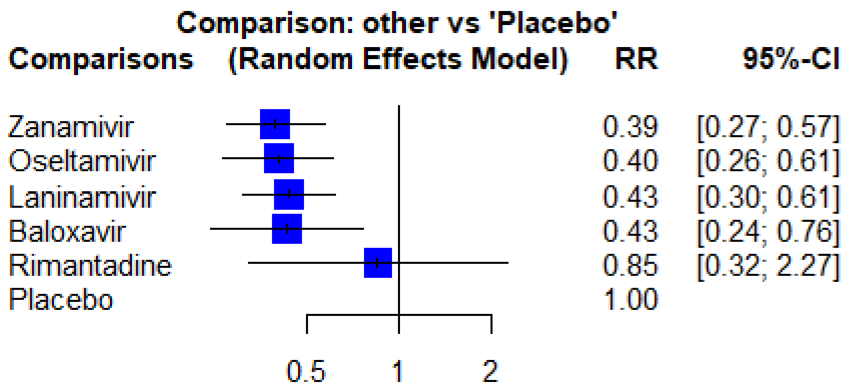

Figure 1 Sensitivity analysis for antivirals prophylaxis against laboratory-confirmed symptomatic influenza by only including the trials with lower risk of bias.

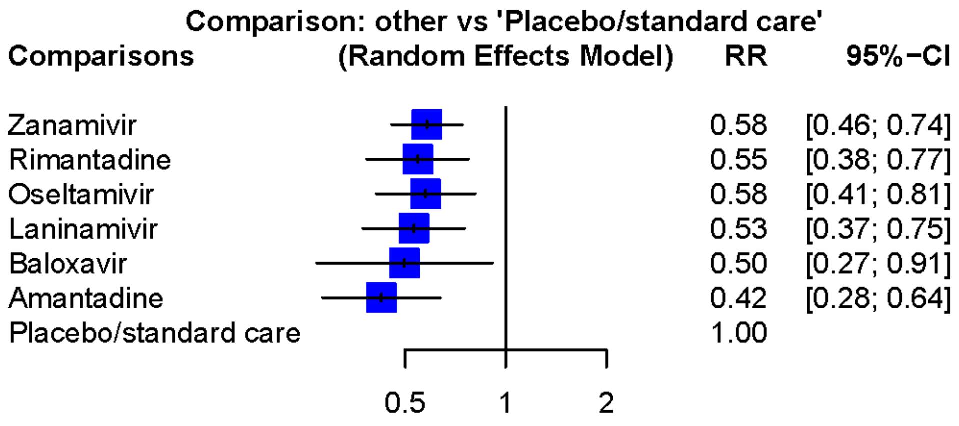

Figure 2 Sensitivity analysis for antivirals prophylaxis against laboratory-confirmed influenza by only including the trials with lower risk of bias.

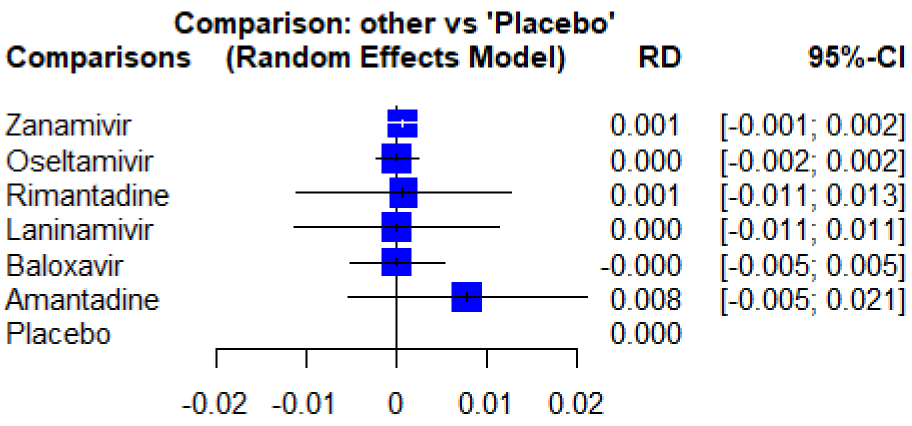

Figure 3 Sensitivity analysis for antivirals prophylaxis against mortality by only including the trials with lower risk of bias.

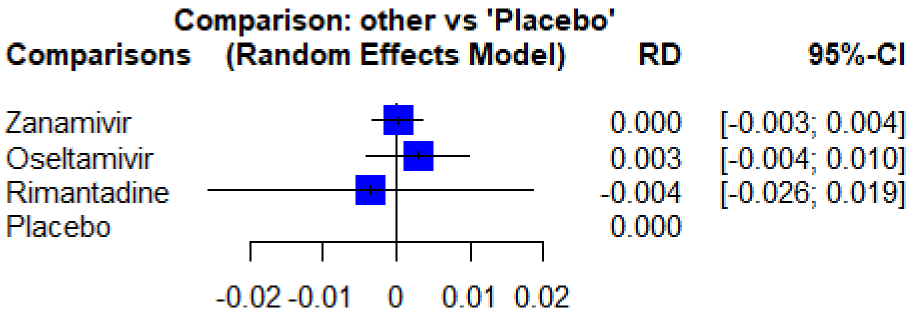

Figure 4 Sensitivity analysis for antivirals prophylaxis against serious adverse events by only including the trials with lower risk of bias.

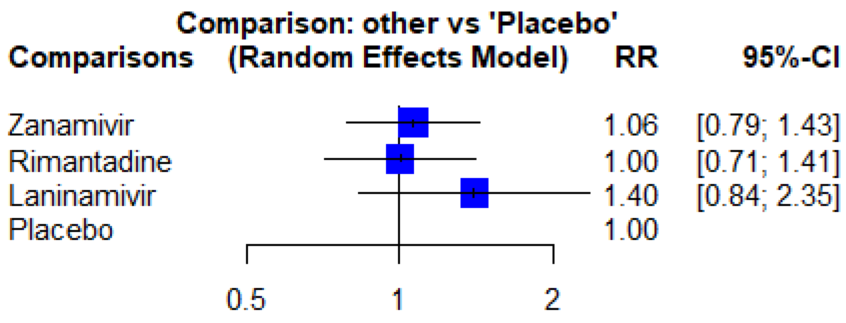

Figure 5 Sensitivity analysis for antivirals prophylaxis against adverse events related to drugs by only including the trials with lower risk of bias.

Appendix 17 Plausible worst-case sensitivity analyses

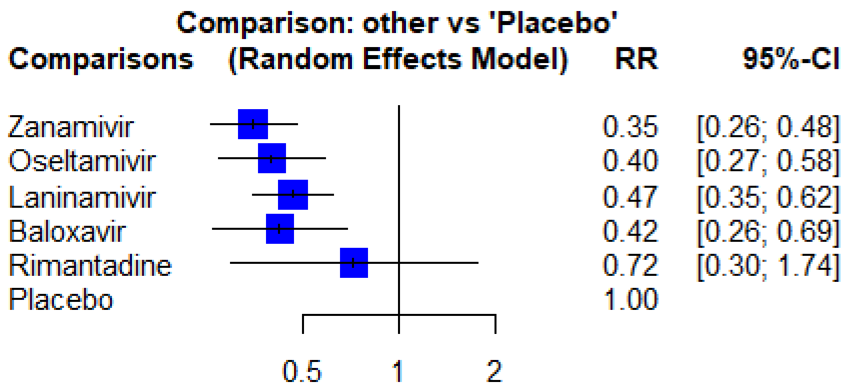

Figure **1** Plausible worst-case sensitivity analyses for antivirals prophylaxis against laboratory-confirmed symptomatic influenza.

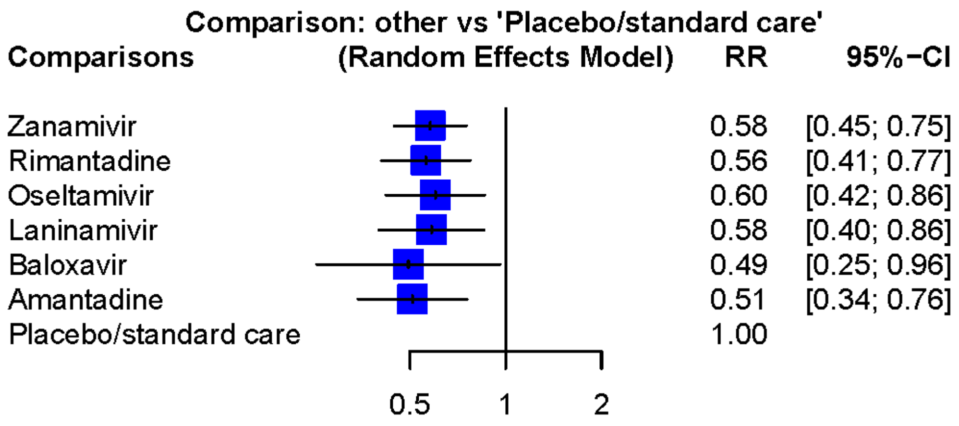

Figure 2 Plausible worst-case sensitivity analyses for antivirals prophylaxis against laboratory-confirmed influenza.

Appendix 18 Sensitivity analysis according to the evidence of exposure to influenza

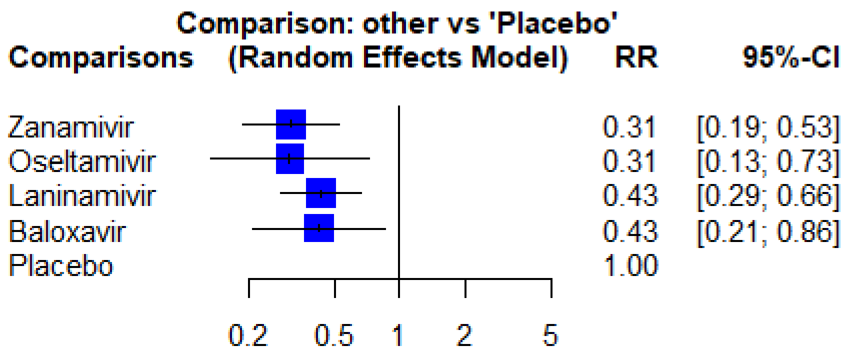

Figure 1 Sensitivity analysis for antivirals prophylaxis against laboratory-confirmed symptomatic influenza by including the trials with clear evidence of exposure to influenza.

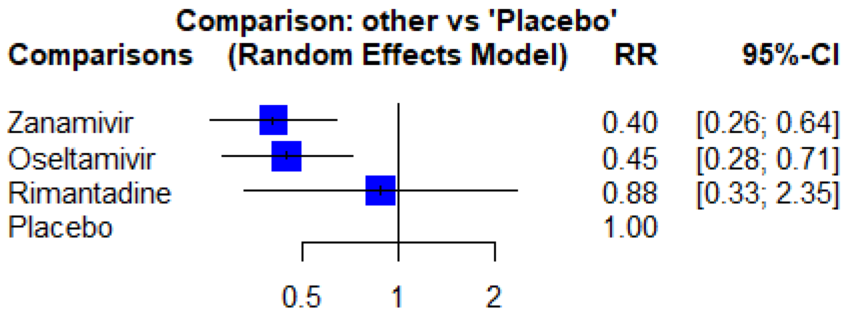

Figure 2 Sensitivity analysis for antivirals prophylaxis against laboratory-confirmed symptomatic influenza by excluding the trials with clear evidence of exposure to influenza.

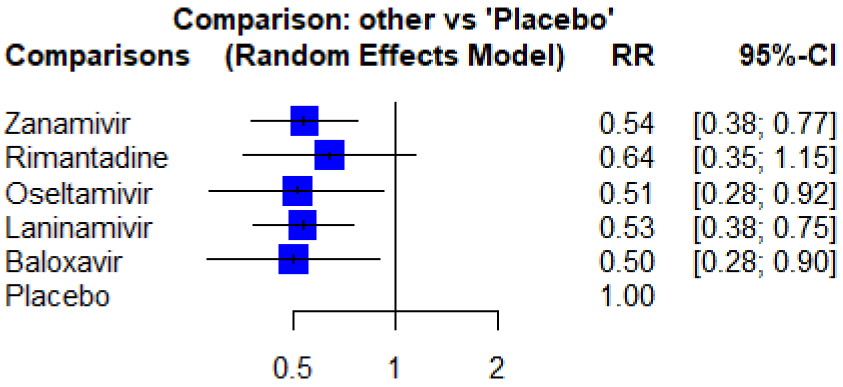

Figure 3 Sensitivity analysis for antivirals prophylaxis against laboratory-confirmed influenza by including the trials with clear evidence of exposure to influenza.

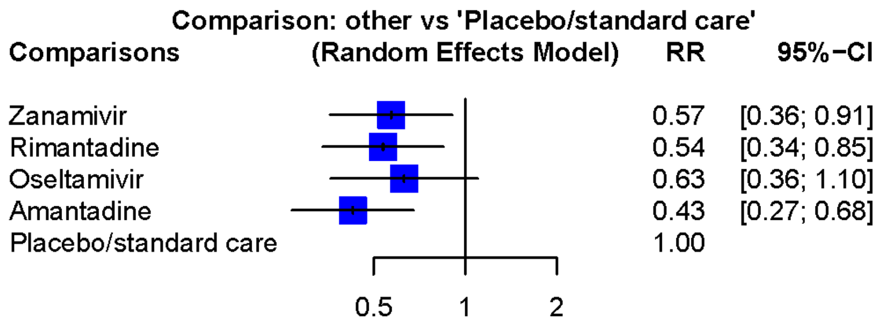

Figure 4 Sensitivity analysis for antivirals prophylaxis against laboratory-confirmed influenza by including the trials with less evidence of exposure to influenza.

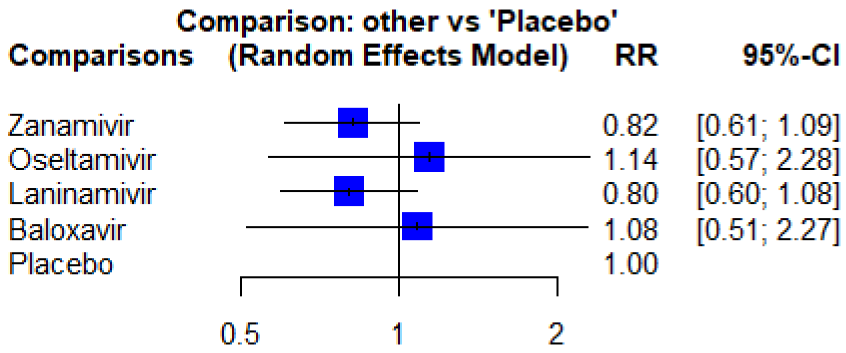

Figure 5 Sensitivity analysis for antivirals prophylaxis against laboratory-confirmed asymptomatic influenza by including the trials with clear evidence of exposure to influenza.

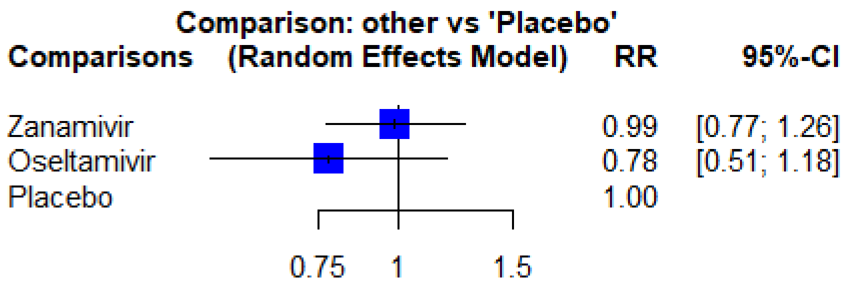

Figure 6 Sensitivity analysis for antivirals prophylaxis against laboratory-confirmed asymptomatic influenza by excluding the trials with clear evidence of exposure to influenza.

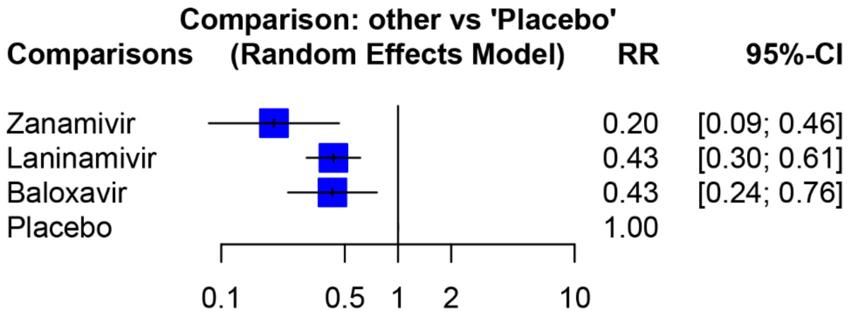

Figure 7 Sensitivity analysis for antivirals prophylaxis against laboratory-confirmed symptomatic influenza by only including the trials with close contact with the index with confirmed influenza.

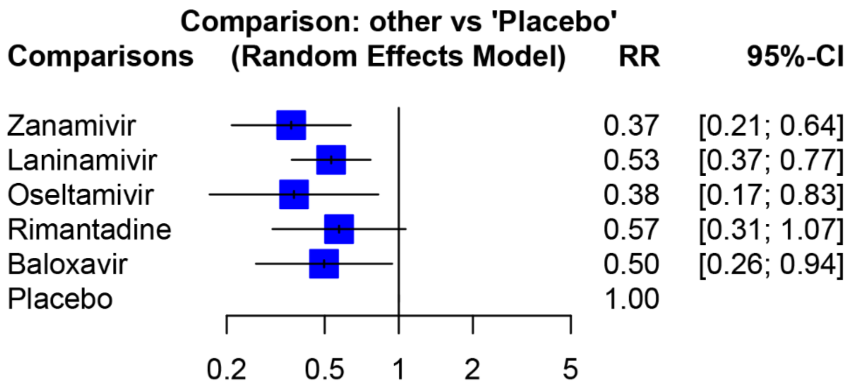

Figure 8 Sensitivity analysis for antivirals prophylaxis against laboratory-confirmed influenza by only including the trials with close contact with the index with confirmed influenza.

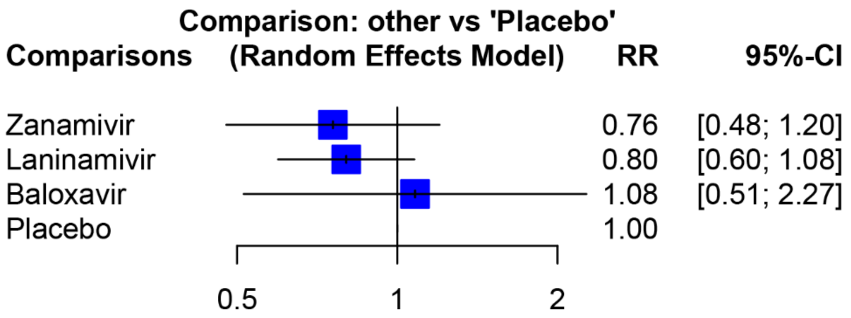

Figure 9 Sensitivity analysis for antivirals prophylaxis against laboratory-confirmed asymptomatic influenza by only including the trials with close contact with the index with confirmed influenza.

Appendix 19 Sensitivity analysis includes only the trials that reported laboratory-confirmed influenza A and influenza B separately.

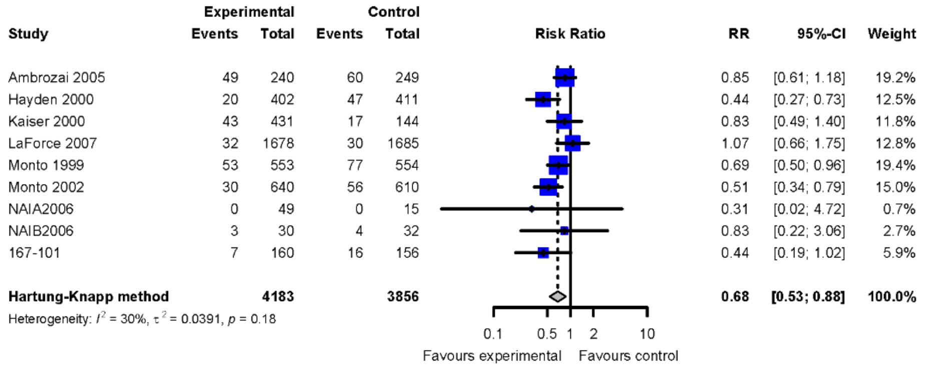

Figure 1 Analysis for zanamivir prophylaxis against laboratory-confirmed influenza A (includes trials that only reported laboratory-confirmed influenza A).

Study	Experimental Events	Experimental Total	Control Events	Control Total	RR	95%-CI	Weight
Hayden 2000	4	402	17	411	0.26	[0.09; 0.73]	20.8%
Kaiser 2000	17	431	6	144	0.90	[0.37; 2.17]	24.3%
LaForce 2007	3	1678	20	1685	0.17	[0.06; 0.53]	18.6%
Monto 2002	2	640	3	610	0.68	[0.13; 3.44]	11.5%
NAIA2006	1	49	2	15	0.19	[0.03; 1.31]	8.6%
NAIB2006	1	30	0	32	1.60	[0.13; 19.12]	5.7%
Schilling 1988	0	26	1	13	0.34	[0.03; 3.91]	5.9%
167-101	0	160	0	156	0.98	[0.06; 15.45]	4.7%
**Hartung-Knapp method**		**3416**		**3066**	**0.42**	**[0.22; 0.83]**	**100.0%**

Heterogeneity: I^2 = 19%, τ^2 = 0.2283, p = 0.28

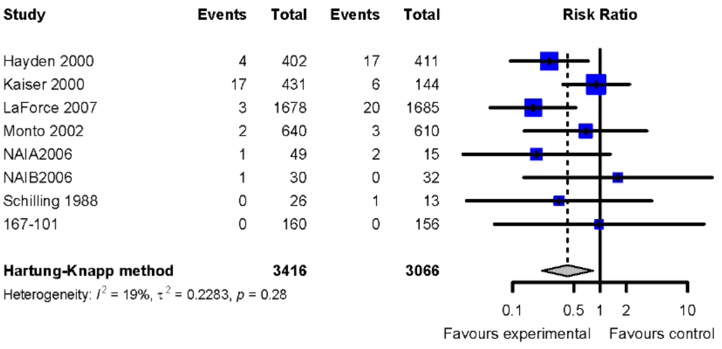

Figure 2 Analysis for zanamivir prophylaxis against laboratory-confirmed influenza B (includes trials that only reported laboratory-confirmed influenza B).

Appendix 20 Sensitivity analysis by using a higher ICC (0.10) to adjust cluster design effect.

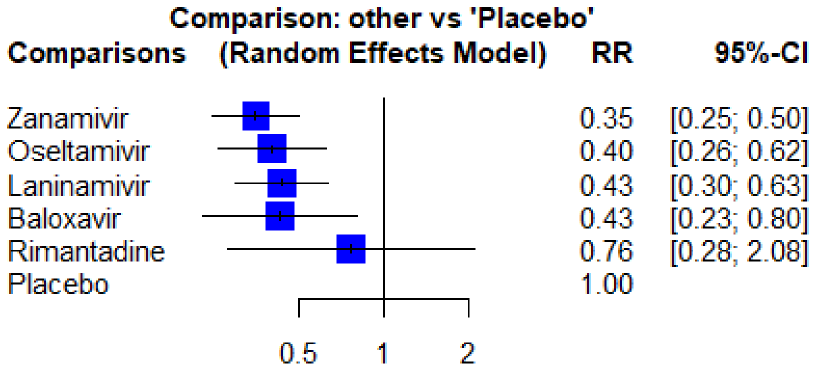

Figure 1 Sensitivity analysis for antivirals prophylaxis against laboratory-confirmed symptomatic influenza (ICC = 0.1).

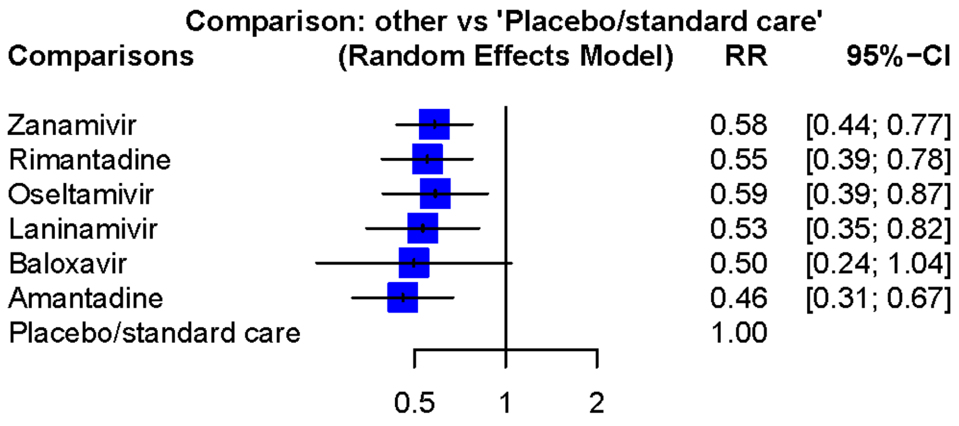

Figure 2 Sensitivity analysis for antivirals prophylaxis against laboratory-confirmed influenza (ICC = 0.1).

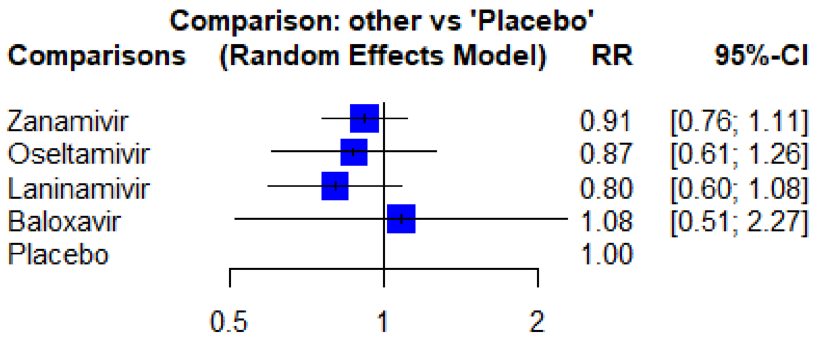

Figure 3 Sensitivity analysis for antivirals prophylaxis against laboratory-confirmed asymptomatic influenza (ICC = 0.1).

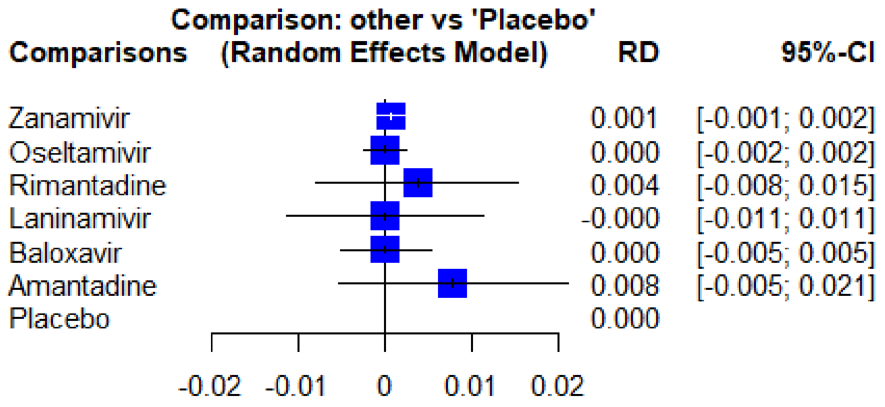

Figure 4 Sensitivity analysis for antivirals prophylaxis against all-cause mortality (ICC = 0.1).

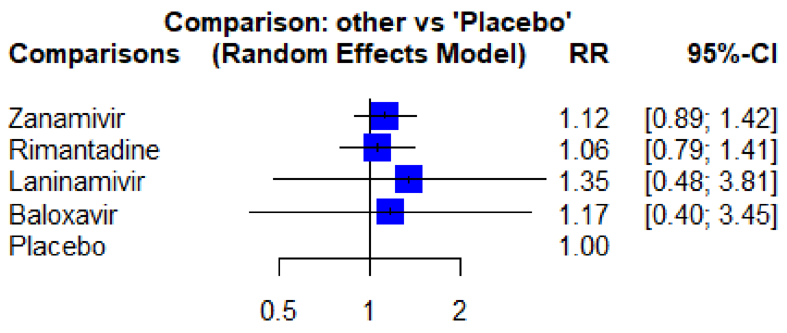

Figure 5 Sensitivity analysis for antivirals prophylaxis against serious adverse events (ICC = 0.1).

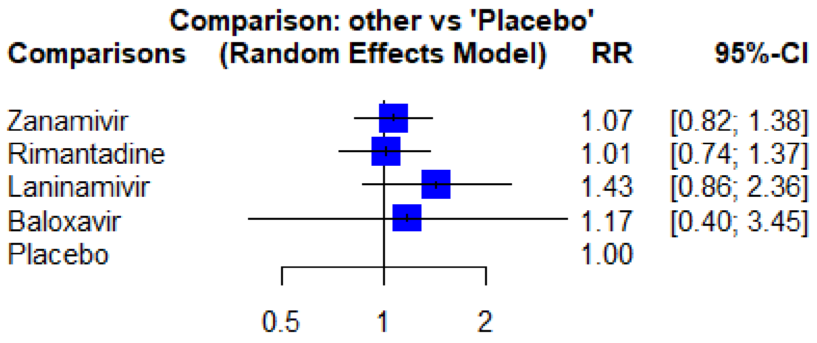

Figure 6 Sensitivity analysis for antivirals prophylaxis against adverse events related to drugs (ICC = 0.1).

Appendix 21 Subgroup analysis

Study	Zanamivir Events	Zanamivir Total	Placebo Events	Placebo Total	RR	95%-CI	Ratio of RRs	95%-CI
Ambrozaitis 2005								
Ambrozaitis 2005_>=65 years	9	152	14	170	0.73	[0.33; 1.61]	1.20	[0.50; 2.85]
Ambrozaitis 2005_<65 years	6	88	9	79	0.61	[0.24; 1.59]		
LaForce 2007								
LaForce 2007_>=65 years	1	946	5	950	0.27	[0.05; 1.66]	1.42	[0.33; 6.04]
LaForce 2007_<65 years	3	732	18	735	0.19	[0.06; 0.59]		
167-101								
167-101_>=35 years	2	57	2	55	0.97	[0.17; 5.36]	3.03	[0.51; 18.09]
167-101_<35 years	1	104	4	101	0.32	[0.05; 2.01]		
**Pooled Ration of RRs** **P=0.31**							1.43	[0.72; 2.84]

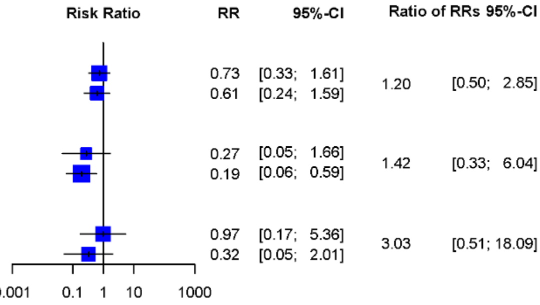

Figure 1 Within-subgroup analysis of laboratory-confirmed influenza according to age (zanamivir versus placebo)

Study	Zanamivir Events	Zanamivir Total	Placebo Events	Placebo Total	RR	95%-CI	Ratio of RRs	95%-CI
Ambrozaitis 2005								
Ambrozaitis 2005_vaccined	0	23	0	22	0.96	[0.06; 14.40]	1.43	[0.27; 7.67]
Ambrozaitis 2005_unvaccined	15	202	23	204	0.67	[0.36; 1.23]		
LaForce 2007								
LaForce 2007_vaccined	1	1115	6	1135	0.23	[0.04; 1.38]	1.21	[0.28; 5.20]
LaForce 2007_unvaccined	3	559	17	527	0.19	[0.06; 0.59]		
Monto 1999								
Monto 1999_vaccined	0	80	6	73	0.14	[0.02; 1.13]	0.36	[0.08; 1.70]
Monto 1999_unvaccined	11	462	28	447	0.39	[0.20; 0.76]		
**Pooled ratio of RRs** **P=0.715**							0.85	[0.34; 2.08]

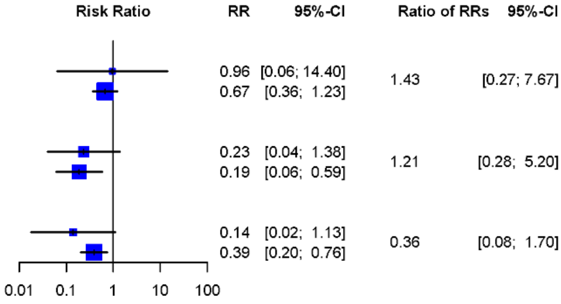

Figure 2 Within-subgroup analysis of laboratory-confirmed influenza according to vaccination status (zanamivir versus placebo)

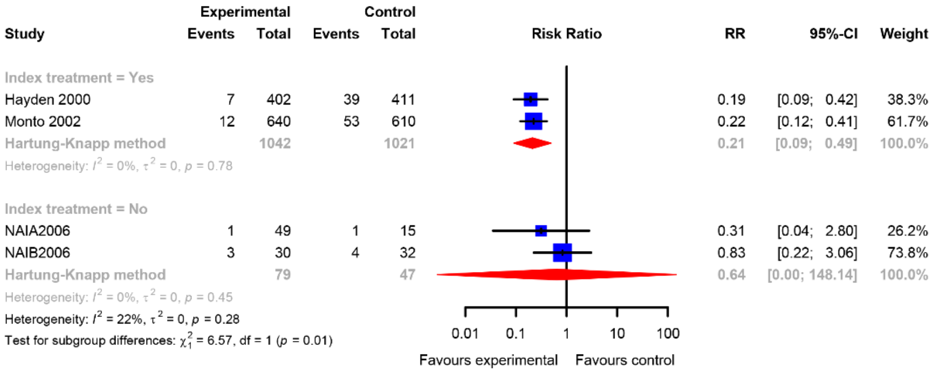

Figure 3 Within-subgroup analysis of laboratory-confirmed influenza according to the status of index patients treatment (zanamivir versus placebo)

Appendix 22: Credibility assessment of subgroup analysis

Text 1 Credibility assessment of treatment of index patients

Credibility assessment

1: Is the analysis of effect modification based on comparison within rather than between trials?

[**X**] Completely between	[ ] Mostly between or unclear	[ ] Mostly within	[ ] Completely within
Subgroup analysis or meta-regression comparing overall effects of each individual trial. This is typical for aggregate data meta-analysis.	*Subgroup analysis or meta-regression with most information coming from overall effects, but some trials providing within-trial subgroup information*	*Most trials providing within-trial subgroup information; or individual participant data analysis that combines within and between trial information*	*All trials providing within-trial subgroup information or individual participant data; and the analysis separates within from between trial information, e.g., meta-analysis of interactions*

2: For within-trial comparisons, is the effect modification similar from trial to trial? [**X**] Not applicable: no or one within-RCT comparison

[ ] Definitely not similar	[ ] Probably not similar or unclear	[ ] Mostly similar	[ ] Definitely similar
Effect modification reported for two or more trials and clearly different directions	*Effect modification not reported for individual trials or too imprecise to tell*	*Effect modification reported for two or more trials, mostly similar in direction, but considerable differences in magnitude*	*Effect modification reported for two or more trials, similar in direction, only some differences in magnitude*

Comment:

3: For between-trial comparisons, is the number of trials large? [ ] Not applicable: no between RCT comparison

[**X**] Very small	[ ] Rather small or unclear	[ ] Rather large	[ ] Large
1 or 2 or in smallest subgroup; 5 or less in continuous meta-regression	*3-4 in smallest subgroup; 6-10 in continuous meta-regression*	*5-9 in smallest subgroup; 11 to 15 in continuous meta-regression*	*10 or more in smallest subgroup; more than 15 in continuous meta-regression*

Comment:

4: Was the direction of effect modification correctly hypothesized a priori?

[**X**] Definitely no	[ ] Probably no or unclear	[ ] Probably yes	[ ] Definitely yes
Clearly post-hoc or results inconsistent with hypothesized direction or biologically very implausible	*Vague hypothesis or hypothesized direction unclear*	*No prior protocol available but unequivocal statement of a priori hypothesis with correct direction of effect modification*	*Prior protocol available and includes correct specification of direction of effect modification, e.g., based on a biologic rationale*

Comment: We didn't hypothesize a priori in the protocol.

5: Does a test for interaction suggest that chance is an unlikely explanation of the apparent effect modification? (consider irrespective of number of effect modifiers)

[ ] Chance a very likely explanation	[ ] Chance a likely explanation or unclear	[**X**] Chance may not explain	[ ] Chance an unlikely explanation

Interaction or meta-regression p-value >0.05	*Interaction or meta-regression p-value ≤0.05 and >0.01, or no test of interaction reported and not computable*	*Interaction or meta-regression p-value ≤0.01 and >0.005*	*Interaction or meta-regression p-value ≤0.005*

Comment: The interaction P-value = 0.01.

6: Did the authors test only a small number of effect modifiers or consider the number in their statistical analysis?

[ ] Definitely no	[ ] Probably no or unclear	[**X**] Probably yes	[ ] Definitely yes
Explicitly exploratory analysis or large number of effect modifiers tested (e.g., greater than 10) and multiplicity not considered in analysis	*No mention of number or 4-10 effect modifiers tested and number not considered in analysis*	*No protocol available but unequivocal statement of 3 or fewer effect modifiers tested*	*Protocol available and 3 or fewer effect modifiers tested or number considered in analysis*

Comment: Three effect modifiers were tested in this review.

7: Did the authors use a random effects model? [ ] Not applicable

[ ] Definitely no	[ ] Probably no or unclear	[ ] Probably yes	[**X**] Definitely yes
Fixed (or common) effect or fixed effects model explicitly stated	*Probably fixed effect(s) model*	*Probably random (or mixed) effects*	*Random (or mixed) effects explicitly stated*

Comment:

8: If the effect modifier is a continuous variable, were arbitrary cut points avoided? [**X**] not applicable: not continuous

[ ] Definitely no	[ ] Probably no or unclear	[ ] Probably yes	[ ] Definitely yes
Analysis based on exploratory cut point(s), e.g., picking cut point associated with highest interaction p-value	*Analysis based on cut point(s) of unclear origin*	*Analysis based on pre-specified cut point(s), e.g., suggested by prior RCT*	*Analysis based on the full continuum, e.g., assuming a linear or logarithmic relationship*

9 Optional: Are there any additional considerations that may increase or decrease credibility? (manual section 3.9) [**X**] not applicable

	[ ] Yes, probably decrease	[ ] Yes, probably increase
	Biologically implausible	
	Expect similar severe critical	
	Opposite effects unlikely	

Comment:

10: How would you rate the overall credibility of the proposed effect modification?

The overall rating should be driven by the items that decrease credibility. The following provides a sensible strategy:

- All responses definitely or probably decrease credibility or unclear → very low
- Two or more responses definitely decrease credibility → maximum usually low even if all other responses satisfy credibility criteria
- One response definitely decreases credibility → maximum usually moderate even if all other responses satisfy credibility criteria
- Two responses probably decrease credibility → maximum usually moderate even if all other responses satisfy credibility criteria
- No response options definitely or probably decrease credibility → high very likely

Place a mark on the continuous line (or type "x" in editable version)

X

**Very low credibility**	**Low credibility**	**Moderate credibility**	**High credibility**
Very likely no effect modification<br>Use overall effect for each subgroup	Likely no effect modification<br>Use overall effect for each subgroup<br>but note remaining uncertainty	Likely effect modification<br>Use separate effects for each subgroup<br>but note remaining uncertainty	Very likely effect modification<br>Use separate effects for each subgroup

Comment:
